# Supplementary material for: Plant-Wide Target Metabolomics Provides a Novel Interpretation of the Changes in Chemical Components during Dendrobium officinale Traditional Processing
Source: Antioxidants (Basel). 2023 Nov 12;12(11):1995. doi: 10.3390/antiox12111995 (PMC10669339; doi:10.3390/antiox12111995)
Supplement: Supplementary file 1 [file antioxidants-12-01995-s001.zip › antioxidants-2656908-SI.pdf]

Supplementary data

Plant-Wide Target Metabolomics Provides a Novel interpretation of the Changes in Chemical Components during *Dendrobium officinale* traditional Processing

Pengfei Liu †, Bei Fan †, Yuwen Mu †, Litao Tong, Cong Lu, Long Li, Jiameng Liu, Jing Sun * and Fengzhong Wang *

Institute of Food Science and Technology, Chinese Academy of Agricultural Sciences, Key Laboratory of Agro-Products Processing, Ministry of Agriculture and Rural Affairs, Beijing 100193, China; liupengfei0424@zidd.ac.cn (P.L.); fanbei@caas.cn (B.F.); muyw@gsagr.cn (Y.M.); tonglitao@caas.cn (L.T.); lucong@caas.cn (C.L.); lilong02@caas.cn (L.L.); liujiameng@caas.cn (J.L.)

* Correspondence: sunjing01@caas.cn (J.S.); wangfengzhong@caas.cn (F.W.); Tel.: +86-10-62810295 (J.S.); +86-10-62817417 (F.W.)

† These authors contributed equally to this work.

No.	Content
**Figure S1.**	Scores plot generated from OPLS-DA model in each group. FA1 *vs.* FB2 groups (A), FB2 vs. FC3 group (B), FC3 *vs.* FD4 group (C), and FD4 *vs.* FE5 group (D).
**Figure S2.**	Chart generated from 200 times permutation test in each group. FA1 vs. FB2 groups (A), the FB2 vs. FC3 groups (B), the FC3 vs. FD4 group (C). and the FD4 vs. FE5 group (D).
**Figure S3.**	HCA map of differential components of in FA1 vs. FE5 group. Differences in phenolic acids (A), flavonoids (B), amino acids and their derivatives (C), and differences in lipids (D).
**Figure S4.**	KEGG pathway analysis of differential chemical compositions for four Class (phenolic acids, flavonoids, amino acids and their derivatives, lipids). KEGG pathway for amino acids and their derivatives (A), the lipids (B), the phenolic acids (C), the flavonoids (D). In the plot, each bubble (which represents a metabolic pathway) and abscissa indicate the size of the factors affecting the pathway (bigger bubbles represent bigger impacts). Bubble color indicates the p-value of the enrichment analysis. Lighter colors indicate lower enrichment.
**Table S1**	Identification results of all chemical compositions in DO

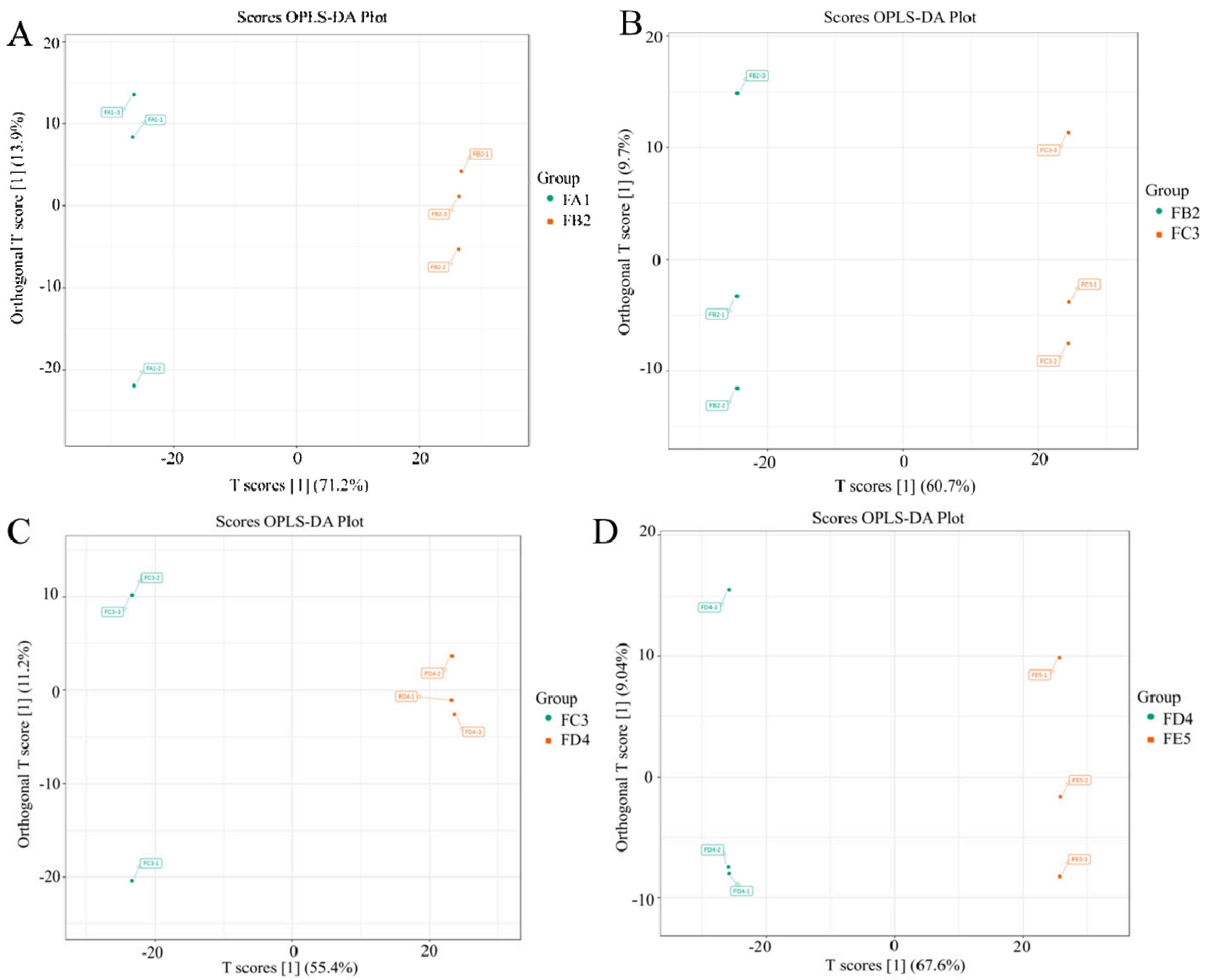

Figure S1. Scores plot generated from OPLS-DA model in each group. FA1 *vs.* FB2 groups (A), FB2 vs. FC3 group (B), FC3 *vs.* FD4 group (C), and FD4 *vs.* FE5 group (D).

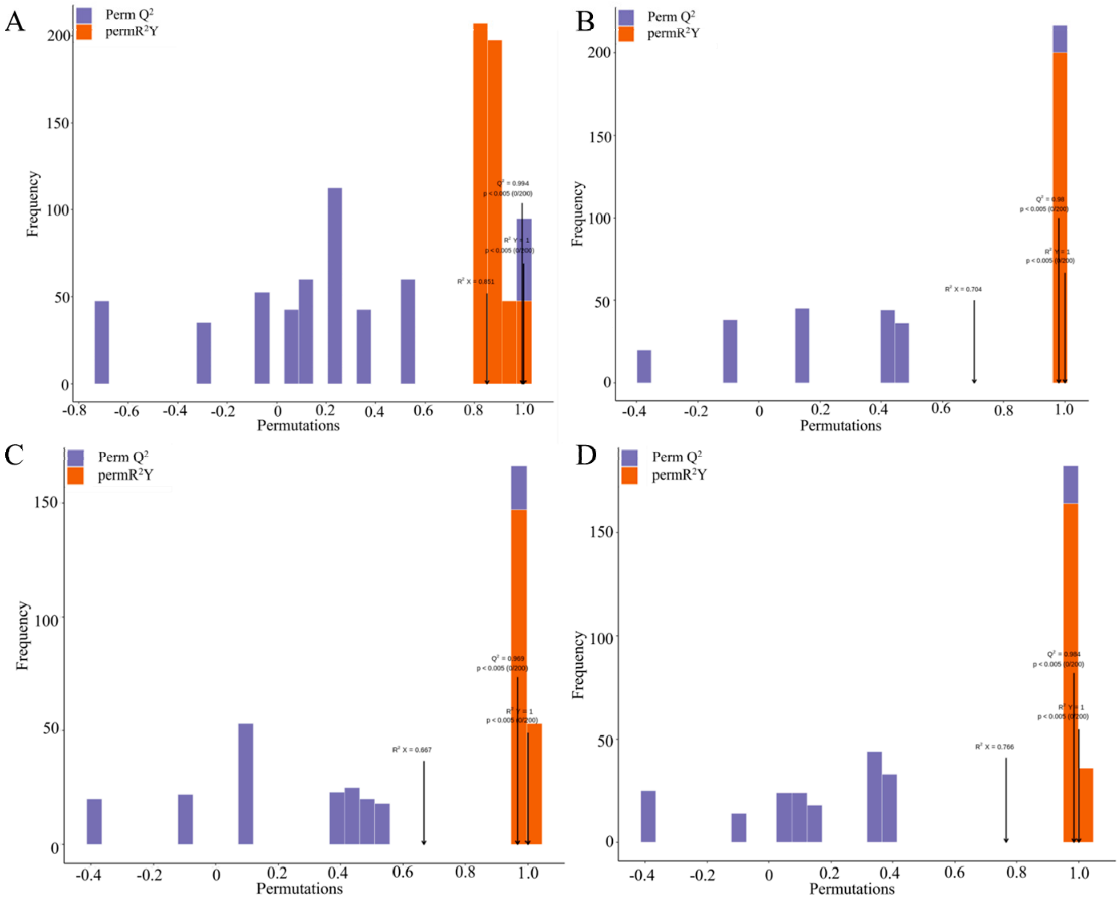

Figure S2. Chart generated from 200 times permutation test in each group. FA1 *vs.* FB2 group (A), FB2 *vs.* FC3 group (B), FC3 *vs.* FD4 group (C). and FD4 *vs.* FE5 group (D).

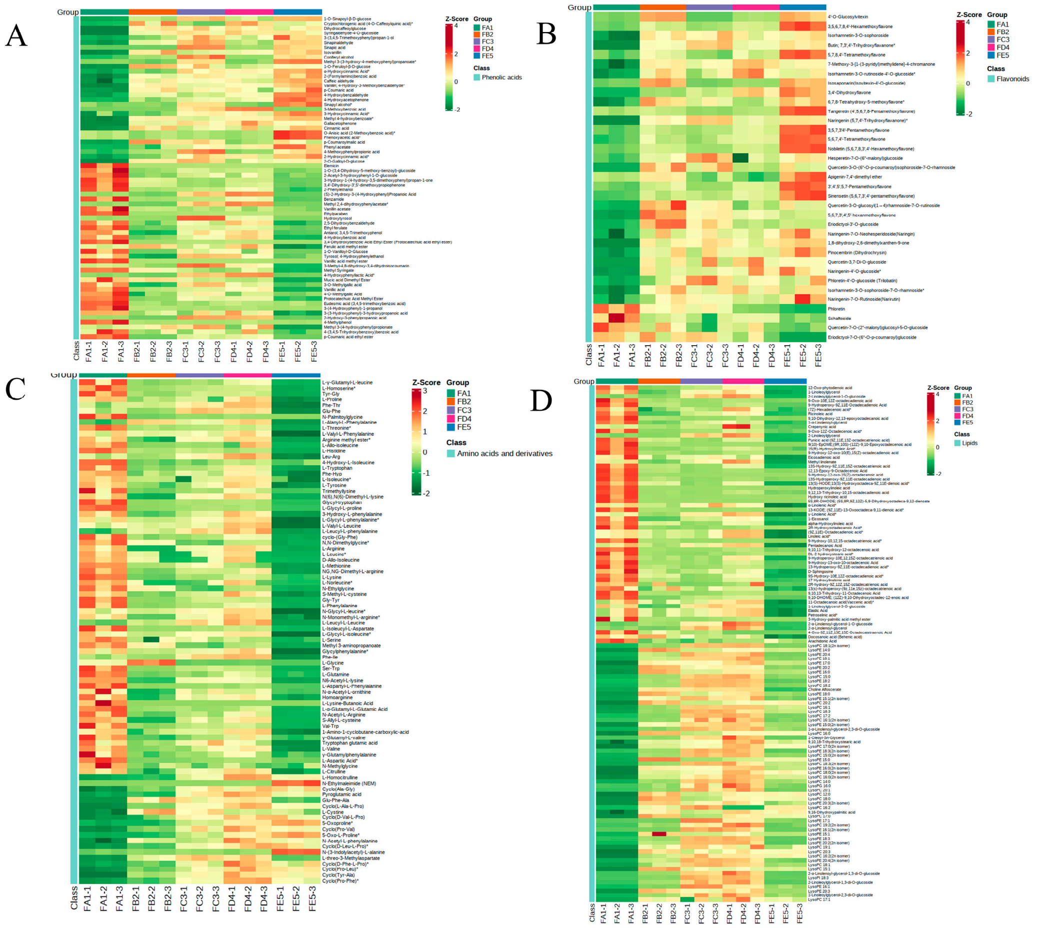

Figure S3. HCA map of differential components in FA1 *vs.* FE5 group. . Differences in phenolic acids (A), Differences in flavonoids (B), Differences in amino acids and their derivatives (C), and differences in lipids (D).

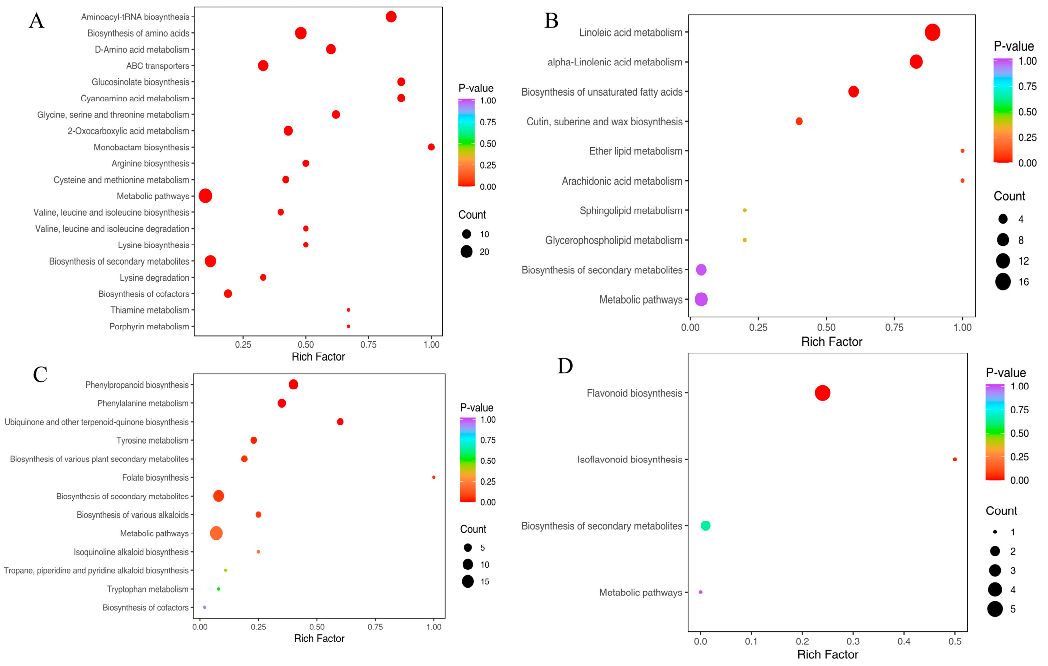

Figure S4. KEGG pathway analysis of differential chemical compositions for four Class (phenolic acids, flavonoids, amino acids and their derivatives, lipids). KEGG pathway for amino acids and their derivatives (A), the lipids (B), the phenolic acids (C), the flavonoids (D). In the plot, each bubble (which represents a metabolic pathway) and abscissa indicate the size of the factors affecting the pathway (bigger bubbles represent bigger impacts). Bubble color indicates the p-value of the enrichment analysis. Lighter colors indicate lower enrichment.

Table S1 Identification results of all chemical compositions in DO

Number	Compounds	Formula	Ionization model	Type	Peak area				
					FA1	FB2	FC3	FD4	FE5
1	L-γ-Glutamyl-L-leucine	$C_{11}H_{20}N_2O_5$	$[M+H]^+$	Amino acids and derivatives	605421	248228	267792	386586	143048
2	L-Homoserine*	$C_4H_9NO_3$	$[M+H]^+$	Amino acids and derivatives	3346953	1517704	1731007	1830747	772030
3	Cycloleucine	$C_6H_{11}NO_2$	$[M+H]^+$	Amino acids and derivatives	2856446	3171362	5106498	7111748	4978656
4	Tyr-Gly	$C_{11}H_{14}N_2O_4$	$[M+H]^+$	Amino acids and derivatives	848957	260028	351499	368940	39390
5	L-Proline	$C_5H_9NO_2$	$[M+H]^+$	Amino acids and derivatives	944566	686399	698029	916611	454889
6	Phe-Thr	$C_{13}H_{18}N_2O_4$	$[M+H]^+$	Amino acids and derivatives	543675	600176	810948	966098	76632
7	Glu-Phe	$C_{14}H_{18}N_2O_5$	$[M+H]^+$	Amino acids and derivatives	431328	318884	711703	646955	46754
8	N-Palmitoylglycine	$C_{18}H_{35}NO_3$	$[M-H]^-$	Amino acids and derivatives	54724	7320	10638	12193	10334
9	L-Homocitrulline	$C_7H_{15}N_3O_3$	$[M+H]^+$	Amino acids and derivatives	964950	1197199	2115667	3086824	2251761
10	L-Alanyl-L-Phenylalanine	$C_{12}H_{16}N_2O_3$	$[M+H]^+$	Amino acids and derivatives	519769	1495427	1068905	1474906	185627
11	Homoproline	$C_6H_{11}NO_2$	$[M+H]^+$	Amino acids and derivatives	4828622	4266581	4699032	9611612	9037646
12	L-Threonine*	$C_4H_9NO_3$	$[M+H]^+$	Amino acids and	3292135	1579953	1636023	1830986	728713

				derivatives					
13	L-Methionine Sulfoxide	$C_5H_{11}NO_3S$	$[M+H]^+$	Amino acids and derivatives	8217603	8123731	8049554	7942515	7306520
14	L-Valyl-L-Phenylalanine	$C_{14}H_{20}N_2O_3$	$[M+H]^+$	Amino acids and derivatives	2307551	1960542	2004553	2501121	184992
15	L-Glutamic acid	$C_5H_9NO_4$	$[M+H]^+$	Amino acids and derivatives	5584748	19577579	20344320	16671648	7604039
16	Arginine methyl ester*	$C_7H_{16}N_4O_2$	$[M+H]^+$	Amino acids and derivatives	3200870	1512266	2769214	3201359	1217086
17	L-Allo-isoleucine	$C_6H_{13}NO_2$	$[M+H]^+$	Amino acids and derivatives	65320163	36890056	38972515	52565836	28358880
18	L-Histidine	$C_6H_9N_3O_2$	$[M+H]^+$	Amino acids and derivatives	5171612	2162171	2425533	2246138	797787
19	Leu-Arg	$C_{12}H_{25}N_5O_3$	$[M+H]^+$	Amino acids and derivatives	797509	637710	1063931	1428458	53327
20	4-Hydroxy-L-Isoleucine	$C_6H_{13}NO_3$	$[M+H]^+$	Amino acids and derivatives	425507	140926	158581	204322	143365
21	N-Ethylmaleimide (NEM)	$C_6H_7NO_2$	$[M+H]^+$	Amino acids and derivatives	65362	346400	302226	523872	7624312
22	N-Acetyl-L-tyrosine	$C_{11}H_{13}NO_4$	$[M+H]^+$	Amino acids and derivatives	55529	24409	85814	120552	85484
23	L-Tryptophan	$C_{11}H_{12}N_2O_2$	$[M-H]^-$	Amino acids and derivatives	9010977	5179420	5811780	7190617	3766377
24	Phe-Hyp	$C_{14}H_{18}N_2O_4$	$[M+H]^+$	Amino acids and derivatives	43614	24514	29311	34387	11938
25	N-acetyl-beta-alanine	$C_5H_9NO_3$	$[M-H]^-$	Amino acids and derivatives	36291	19476	11047	24863	23345
26	L-Isoleucine*	$C_6H_{13}NO_2$	$[M+H]^+$	Amino acids and	3087661	1845265	1507195	2787288	606122

				derivatives					
27	L-Tyrosine	$C_9H_{11}NO_3$	$[M+H]^+$	Amino acids and derivatives	26545465	16802913	20948953	25533084	11493049
28	L-Cyclopentylglycine	$C_7H_{13}NO_2$	$[M+H]^+$	Amino acids and derivatives	943764	1314918	943457	861439	931774
29	Trimethyllysine	$C_9H_{20}N_2O_2$	$[M+H]^+$	Amino acids and derivatives	3652832	805615	1196915	1820041	574160
30	Cyclo(Ser-Pro)	$C_8H_{12}N_2O_3$	$[M+H]^+$	Amino acids and derivatives	178073	204325	246818	399456	233436
31	N(6),N(6)-Dimethyl-L-lysine	$C_8H_{18}N_2O_2$	$[M+H]^+$	Amino acids and derivatives	24934002	8950551	21495605	25002784	8720977
32	Glycyl-tryptophan	$C_{13}H_{15}N_3O_3$	$[M+H]^+$	Amino acids and derivatives	666562	104227	147766	155429	9
33	N-Acetyl-L-glutamic acid	$C_7H_{11}NO_5$	$[M-H]^-$	Amino acids and derivatives	140546	164145	136163	204125	192896
34	L-Glycyl-L-proline	$C_7H_{12}N_2O_3$	$[M+H]^+$	Amino acids and derivatives	1637573	258856	379381	369575	36825
35	3-Hydroxy-L-phenylalanine	$C_9H_{11}NO_3$	$[M+H]^+$	Amino acids and derivatives	27058260	15555670	20527223	25890451	11756447
36	L-Glycyl-L-phenylalanine*	$C_{11}H_{14}N_2O_3$	$[M+H]^+$	Amino acids and derivatives	2357287	1700480	2012014	2556282	210583
37	L-Valyl-L-Leucine	$C_9H_{11}NO_3$	$[M+H]^+$	Amino acids and derivatives	6064454	5676507	5818411	8502584	468001
38	L-Leucyl-L-phenylalanine	$C_{11}H_{14}N_2O_3$	$[M+H]^+$	Amino acids and derivatives	580573	1331661	1973922	2292058	128395
39	cyclo-(Gly-Phe)	$C_9H_{11}NO_3$	$[M+H]^+$	Amino acids and derivatives	31321428	17573250	18426224	22686489	12108940
40	Cyclo(Ala-Gly)	$C_{11}H_{14}N_2O_3$	$[M+H]^+$	Amino acids and	190563	382802	624596	660749	665730

				derivatives					
41	N,N-Dimethylglycine*	$C_9H_{11}NO_3$	$[M+H]^+$	Amino acids and derivatives	3394811	2514002	1612478	2089477	1253874
42	L-Prolyl-L-Phenylalanine	$C_{11}H_{14}N_2O_3$	$[M+H]^+$	Amino acids and derivatives	172557	261977	395417	523087	105208
43	L-Arginine	$C_9H_{11}NO_3$	$[M+H]^+$	Amino acids and derivatives	61311469	22045509	49949669	60723936	19914371
44	L-Leucine*	$C_{11}H_{14}N_2O_3$	$[M+H]^+$	Amino acids and derivatives	3076767	1307311	1472292	1998677	264850
45	L-Asparagine	$C_9H_{11}NO_3$	$[M+H]^+$	Amino acids and derivatives	12218275	11574965	20890185	19220753	7616296
46	Pyroglutamic acid	$C_{11}H_{14}N_2O_3$	$[M+H]^+$	Amino acids and derivatives	788835	985739	1881837	2241970	1637547
47	D-Allo-Isoleucine	$C_9H_{11}NO_3$	$[M+H]^+$	Amino acids and derivatives	3014786	1324233	938577	2047396	263660
48	L-Methionine	$C_{11}H_{14}N_2O_3$	$[M+H]^+$	Amino acids and derivatives	978824	396510	612959	358240	68666
49	NG,NG-Dimethyl-L-arginine	$C_9H_{11}NO_3$	$[M+H]^+$	Amino acids and derivatives	3162984	1318366	1810746	2220207	655631
50	L-Lysine	$C_{11}H_{14}N_2O_3$	$[M+H]^+$	Amino acids and derivatives	202208	42313	64986	88492	29043
51	L-Norleucine*	$C_9H_{11}NO_3$	$[M+H]^+$	Amino acids and derivatives	3096538	770060	951573	2024144	252454
52	N-Ethylglycine	$C_{11}H_{14}N_2O_3$	$[M+H]^+$	Amino acids and derivatives	3398826	2656302	1622696	2054871	1367160
53	S-Methyl-L-cysteine	$C_9H_{11}NO_3$	$[M+H]^+$	Amino acids and derivatives	1073017	634943	810863	1053320	403532
54	Gly-Tyr	$C_{11}H_{14}N_2O_3$	$[M+H]^+$	Amino acids and	855102.	225741	344416	350556	39435

				derivatives					
55	L-Phenylalanine	$C_9H_{11}NO_3$	$[M+H]^+$	Amino acids and derivatives	15610895	6329810	7532951	9672381	3578181
56	N-Glycyl-L-leucine*	$C_{11}H_{14}N_2O_3$	$[M+H]^+$	Amino acids and derivatives	3198130	2736537	3428032	3994282	336012
57	N-Monomethyl-L-arginine*	$C_9H_{11}NO_3$	$[M+H]^+$	Amino acids and derivatives	3142156	1734688	2735408	3368940	1085906
58	L-Leucyl-L-Leucine	$C_{11}H_{14}N_2O_3$	$[M+H]^+$	Amino acids and derivatives	1056368	1858366	3045657	3750223	189410
59	L-Homocystine	$C_9H_{11}NO_3$	$[M+H]^+$	Amino acids and derivatives	286798	576926	552160	549067	418160
60	L-Isoleucyl-L-Aspartate	$C_{11}H_{14}N_2O_3$	$[M+H]^+$	Amino acids and derivatives	3536472	902304	1014306	1286593	146823
61	Glu-Phe-Ala	$C_9H_{11}NO_3$	$[M+H]^+$	Amino acids and derivatives	9	100163	84530	95995	29406
62	N-Acetyl-L-leucine	$C_{11}H_{14}N_2O_3$	$[M+H]^+$	Amino acids and derivatives	433665	105735	163793	208818	290371
63	L-Glycyl-L-isoleucine*	$C_9H_{11}NO_3$	$[M+H]^+$	Amino acids and derivatives	3230880	2289262	2975624	3376743	283169
64	O-Acetylserine	$C_{11}H_{14}N_2O_3$	$[M+H]^+$	Amino acids and derivatives	581671	220781	257232	305598	322664
65	L-Serine	$C_9H_{11}NO_3$	$[M+H]^+$	Amino acids and derivatives	188786	100737	126034	115325	77651
66	Methyl 3-aminopropanoate	$C_{11}H_{14}N_2O_3$	$[M+H]^+$	Amino acids and derivatives	3173106	2477887	1553088	1937932	1192469
67	Glycylphenylalanine*	$C_9H_{11}NO_3$	$[M+H]^+$	Amino acids and derivatives	2329375	1574404	1892246	2402213	197199
68	Phe-Ile	$C_{11}H_{14}N_2O_3$	$[M+H]^+$	Amino acids and	336320	308426	638695	727644	21690

				derivatives					
69	Lys-Asp	$C_9H_{11}NO_3$	$[M+H]^+$	Amino acids and derivatives	521783	533792	620172	608763	660264
70	Cyclo(L-Ala-L-Pro)	$C_{11}H_{14}N_2O_3$	$[M+H]^+$	Amino acids and derivatives	30015	149269	267592	357560	261729
71	L-Cystine	$C_9H_{11}NO_3$	$[M+H]^+$	Amino acids and derivatives	55801	229727	200388	155001	145984
72	Asp-Lys	$C_{11}H_{14}N_2O_3$	$[M+H]^+$	Amino acids and derivatives	553619	575147	605569	621529	651812
73	γ-Glutamyltyrosine	$C_9H_{11}NO_3$	$[M+H]^+$	Amino acids and derivatives	74787	38532	59846	95905	38573
74	L-Glycine	$C_{11}H_{14}N_2O_3$	$[M+H]^+$	Amino acids and derivatives	103239	242407	7277	10698	9
75	Ser-Trp	$C_9H_{11}NO_3$	$[M+H]^+$	Amino acids and derivatives	553317	123726	207461	189799	9
76	Cyclo(Pro-Pro)	$C_{11}H_{14}N_2O_3$	$[M+H]^+$	Amino acids and derivatives	5970	17364	41210	40410	54018
77	N-Acetyl-L-Glutamine	$C_9H_{11}NO_3$	$[M-H]^-$	Amino acids and derivatives	906436	1066574	1097399	1409169	1196220
78	L-Glutamine	$C_{11}H_{14}N_2O_3$	$[M+H]^+$	Amino acids and derivatives	182146	43030	55664	86238	24495
79	N-Methyl-Trans-4-Hydroxy-L-Proline	$C_9H_{11}NO_3$	$[M+H]^+$	Amino acids and derivatives	181219	171707	157870	171797	238002
80	N6-Acetyl-L-lysine	$C_{11}H_{14}N_2O_3$	$[M+H]^+$	Amino acids and derivatives	978866	267860	634864	635385	155170
81	3-Methyl-L-Histidine*	$C_9H_{11}NO_3$	$[M+H]^+$	Amino acids and derivatives	118011	117130	113844	127192	122658
82	1-Methylhistidine*	$C_{11}H_{14}N_2O_3$	$[M+H]^+$	Amino acids and	118011	117130	113844	127192	122658

				derivatives					
83	L-Aspartyl-L-Phenylalanine	$C_9H_{11}NO_3$	$[M+H]^+$	Amino acids and derivatives	1683441	250883	310839	357560	63233
84	N-α-Acetyl-L-ornithine	$C_{11}H_{14}N_2O_3$	$[M-H]^-$	Amino acids and derivatives	686334	224010	502157	448407	271480
85	Homoarginine	$C_9H_{11}NO_3$	$[M+H]^+$	Amino acids and derivatives	507702	205077	376093	422719	160976
86	Cyclo(D-Val-L-Pro)	$C_{11}H_{14}N_2O_3$	$[M+H]^+$	Amino acids and derivatives	20631	48768	92003	109075	93932
87	L-Lysine-Butanoic Acid	$C_9H_{11}NO_3$	$[M+H]^+$	Amino acids and derivatives	11008590	168367	146371	223827	70853
88	L-α-Glutamyl-L-Glutamic Acid	$C_{11}H_{14}N_2O_3$	$[M+H]^+$	Amino acids and derivatives	333696	112998	114522	146529	38530
89	5-Oxoproline*	$C_9H_{11}NO_3$	$[M-H]^-$	Amino acids and derivatives	166542	205182	497766	720545	915317
90	N-Acetyl-L-glycine	$C_{11}H_{14}N_2O_3$	[M-H]-	Amino acids and derivatives	15262	8382	9	9	9652
91	N-Acetyl-L-Tryptophan	$C_9H_{11}NO_3$	$[M-H]^-$	Amino acids and derivatives	1510213	1433952	1883386	1340879	2288421
92	Cyclo(Pro-Val)	$C_{11}H_{14}N_2O_3$	$[M+H]^+$	Amino acids and derivatives	14080	37823	65227	81889	63948
93	S-(Methyl)glutathione	$C_9H_{11}NO_3$	$[M+H]^+$	Amino acids and derivatives	265032	119395	288560	232088	173781
94	Oxiglutatione	$C_{11}H_{14}N_2O_3$	$[M-H]^-$	Amino acids and derivatives	10895	20485	14238	13574	16380
95	N-Acetyl-L-Arginine	$C_9H_{11}NO_3$	$[M+H]^+$	Amino acids and derivatives	226454	66311	95754	136282	40240
96	S-Allyl-L-cysteine	$C_{11}H_{14}N_2O_3$	$[M+H]^+$	Amino acids and	33344	9	16436	26153	9

				derivatives					
97	Val-Trp	$C_9H_{11}NO_3$	$[M+H]^+$	Amino acids and derivatives	667065	173138	246842	266095	28502
98	3-Hydroxy-3-methylpentane-1, 5-dioic acid	$C_{11}H_{14}N_2O_3$	$[M-H]^-$	Amino acids and derivatives	917089	593128	1612293	2860918	714623
99	N-Acetyl-L-Aspartic Acid	$C_9H_{11}NO_3$	$[M-H]^-$	Amino acids and derivatives	141532	48711	77080	126162	161786
100	1-Amino-1-cyclobutane-carbo xylic-acid	$C_{11}H_{14}N_2O_3$	$[M+H]^+$	Amino acids and derivatives	911259	643918	755447	926166	431669
101	5-Oxo-L-Proline*	$C_9H_{11}NO_3$	$[M-H]^-$	Amino acids and derivatives	170522	230356	496592	745846	981504
102	N-Alpha-Acetyl-L-Asparagine	$C_{11}H_{14}N_2O_3$	$[M-H]^-$	Amino acids and derivatives	55130	125163	118735	85877	93379
103	L-Phenylalanyl-L-phenylalanin e	$C_9H_{11}NO_3$	$[M+H]^+$	Amino acids and derivatives	46168	56326	96455	89191	7187
104	γ-Glutamyl-L-valine	$C_{11}H_{14}N_2O_3$	$[M+H]^+$	Amino acids and derivatives	112609	40967	53838	77393	39945
105	N-Acetyl-L-phenylalanine	$C_9H_{11}NO_3$	$[M-H]^-$	Amino acids and derivatives	324349	800151	720774	1085946	835864
106	Tryptophan glutamic acid	$C_{11}H_{14}N_2O_3$	$[M-H]^-$	Amino acids and derivatives	18985	8280	16973	12646	9
107	Cyclo(D-Leu-L-Pro)*	$C_9H_{11}NO_3$	$[M+H]^+$	Amino acids and derivatives	28163	62365	128791	172918	153173
108	L-Valine	$C_{11}H_{14}N_2O_3$	$[M+H]^+$	Amino acids and derivatives	3806988	2322182	2731012	3200260	1517940
109	5-L-Glutamyl-L-amino acid	$C_9H_{11}NO_3$	$[M-H]^-$	Amino acids and derivatives	39131	30743	28261	51260	23631
110	γ-Glutamylphenylalanine	$C_{11}H_{14}N_2O_3$	$[M-H]^-$	Amino acids and	91816	35315	44444	57244	29686

				derivatives					
111	N-(3-Indolylacetyl)-L-alanine	$C_9H_{11}NO_3$	$[M+H]^+$	Amino acids and derivatives	7863	11279	14316	13441	74295
112	L-Tyrosine methyl ester	$C_{11}H_{14}N_2O_3$	$[M+H]^+$	Amino acids and derivatives	67259	44094	48007	63640	76219
113	L-threo-3-Methylaspartate	$C_9H_{11}NO_3$	$[M-H]^-$	Amino acids and derivatives	139743	572905	810633	772336	328328
114	N-Acetyl-L-threonine	$C_{11}H_{14}N_2O_3$	$[M-H]^-$	Amino acids and derivatives	82977	63213	53304	72934	71372
115	Cyclo(D-Phe-L-Pro)*	$C_9H_{11}NO_3$	$[M+H]^+$	Amino acids and derivatives	17466	41479	81314	128863	86552
116	N-Carbamoyl-L-aspartate	$C_{11}H_{14}N_2O_3$	$[M-H]^-$	Amino acids and derivatives	21916	26155	25889	28217	37832
117	Cyclo(Pro-Leu)*	$C_9H_{11}NO_3$	$[M+H]^+$	Amino acids and derivatives	31890	86564	139721	197278	173443
118	L-Aspartic Acid*	$C_{11}H_{14}N_2O_3$	$[M-H]^-$	Amino acids and derivatives	41748490	6805380	8714255	10901341	6029527
119	Cyclo(Tyr-Ala)	$C_9H_{11}NO_3$	$[M+H]^+$	Amino acids and derivatives	9	13956	20841	27450	22150
120	L-Alanyl-L-Alanine	$C_{11}H_{14}N_2O_3$	$[M-H]^-$	Amino acids and derivatives	15370	22753	22731	28016	4182
121	L-Citrulline	$C_9H_{11}NO_3$	$[M+H]^+$	Amino acids and derivatives	471464	158874	355376	459961	88722
122	Cyclo(Pro-Phe)*	$C_{11}H_{14}N_2O_3$	$[M+H]^+$	Amino acids and derivatives	10468	35378	82354	127063	79234
123	N-Methylglycine	$C_9H_{11}NO_3$	$[M-H]^-$	Amino acids and derivatives	31878	10868	10230	11230	9130
124	Confertifoline	$C_{11}H_{14}N_2O_3$	$[M-H]^-$	Terpenoids	1486337	1302606	1504205	1416946	1306415

125	polygodial	$C_9H_{11}NO_3$	$[M+H]^+$	Terpenoids	81680	71000	74991	68836	64140
126	Nootkatone	$C_{11}H_{14}N_2O_3$	$[M+H]^+$	Terpenoids	35157	16065	18816	26094	11080
127	Elemol	$C_9H_{11}NO_3$	$[M+H]^+$	Terpenoids	8383925	9895400	9603332	10214048	10181036
128	Secoxyloganin	$C_{11}H_{14}N_2O_3$	$[M+H]^+$	Terpenoids	56235	37793	37392	35830	29270
129	Dihydrovomifoliol-O-β-D-gluc oside	$C_9H_{11}NO_3$	$[M+H]^+$	Terpenoids	164870	107649	119095	138650	78040
130	6-Hydroxydendroxine	$C_{11}H_{14}N_2O_3$	$[M+H]^+$	Alkaloids	281905	207450	237761	134592	141766
131	N-Methyldendrobine	$C_9H_{11}NO_3$	$[M+H]^+$	Alkaloids	1996402	721532	476642	828021	189203
132	Loliolide	$C_{11}H_{14}N_2O_3$	$[M+H]^+$	Terpenoids	849052	2587919	1743012	3019353	2146262
133	8-Epiloganic acid	$C_9H_{11}NO_3$	$[M-H]^-$	Terpenoids	200004	359508	312449	299827	298755
134	Genipin	$C_{11}H_{14}N_2O_3$	$[M+H]^+$	Terpenoids	99180	94943	93669	78625	73025
135	Gardoside	$C_9H_{11}NO_3$	$[M-H]^-$	Terpenoids	21713	84502	80875	76534	52247
136	Dehydroxysecologanic acid	$C_{11}H_{14}N_2O_3$	$[M+H]^+$	Terpenoids	32268	220301	172680	169635	135523
137	Perillyl alcohol	$C_9H_{11}NO_3$	$[M+H]^+$	Terpenoids	279834	433495	374629	576280	331408
138	3,4-Dihydrocatalposide	$C_{11}H_{14}N_2O_3$	[M-H]-	Terpenoids	142921	53940	72011	96869	45604
139	Syringopicroside	$C_9H_{11}NO_3$	[M-H]-	Terpenoids	428986	511573	549885	529556	580040
140	Loganic acid	$C_{11}H_{14}N_2O_3$	[M-H]-	Terpenoids	30268	27826	21804	22039	24543
141	Vomifoliol	$C_9H_{11}NO_3$	$[M+H]^+$	Terpenoids	325345	222971	209902	240412	138001
142	6-O-Vanilloylajugol	$C_{11}H_{14}N_2O_3$	[M-H]-	Terpenoids	24285	22173	22371	23607	19619
143	6-O-Sinapoylajugol	$C_9H_{11}NO_3$	[M-H]-	Terpenoids	32840	44529	51403	49181	51609
144	Geniposide	$C_{11}H_{14}N_2O_3$	$[M+H]^+$	Terpenoids	348786	387721	390330	336039	280799
145	Deacetylasperulosidic acid	$C_9H_{11}NO_3$	[M-H]-	Terpenoids	5355	8309	8512	9377	7419
146	6-O-Trans-Caffeoyl Ajugol	$C_{11}H_{14}N_2O_3$	[M-H]-	Terpenoids	343957	282042	376326	428665	359005
147	7-Deoxyloganic acid	$C_9H_{11}NO_3$	[M-H]-	Terpenoids	31518	21660	24281	21044	26520
148	Sweroside	$C_{11}H_{14}N_2O_3$	$[M+H]^+$	Terpenoids	274992	259971	173854	240102	172222
149	Asperulosidic acid	$C_9H_{11}NO_3$	[M-H]-	Terpenoids	15084	15474	16133	22607	9
150	8-O-Acetylharpagide	$C_{11}H_{14}N_2O_3$	[M-H]-	Terpenoids	369103	475522	619943	529152	507814
151	3-Carbamyl-1-methylpyridiniu	$C_9H_{11}NO_3$	$[M+H]^+$	Alkaloids	6584048	11701835	6981285	7016431	4121239

	m;(1-Methylnicotinamide)								
152	3-Hydroxypyridine	$C_{11}H_{14}N_2O_3$	$[M+H]^+$	Alkaloids	17362	60973	92519	56599	360696
153	2-Picoline; 2-Methylpyridine	$C_9H_{11}NO_3$	$[M+H]^+$	Alkaloids	152960	166653	151822	172718	174378
154	1,4-Dihydro-1-Methyl-4-oxo-3-pyridinecarboxamide	$C_{11}H_{14}N_2O_3$	[M-H]-	Alkaloids	327431	94384	70497	65513	69069
155	3-pyridine-methanol-O-β-D-glucopyranosyl	$C_9H_{11}NO_3$	$[M+H]^+$	Alkaloids	153840	281329	211034	547479	308599
156	Nicotinic Acid Methyl Ester(Methyl Nicotinate)	$C_{11}H_{14}N_2O_3$	$[M+H]^+$	Alkaloids	14131	30889	57189	42466	222476
157	Quinolinic Acid	$C_9H_{11}NO_3$	[M-H]-	Alkaloids	54137	41599	28984	20883	22646
158	Methyl L-pyroglutamate	$C_{11}H_{14}N_2O_3$	$[M+H]^+$	Alkaloids	469924	991050	1685458	2718534	9850649
159	2-Phenylethylamine	$C_9H_{11}NO_3$	$[M+H]^+$	Alkaloids	4092228	1074433	1270405	1933845	440161
160	Phloretin	$C_{11}H_{14}N_2O_3$	[M-H]-	Flavonoids	566194	392924	369266	219343	155443
161	4'-Hydroxy-2,6-dimethoxydihydrochalcone; Cochinchinenin A	$C_9H_{11}NO_3$	$[M+H]^+$	Flavonoids	6891	23549	15240	11709	10738
162	Sieboldin	$C_{11}H_{14}N_2O_3$	[M-H]-	Flavonoids	54837	85975	113682	106173	77305
163	Dihydrocharcone-4'-O-glucoside	$C_9H_{11}NO_3$	[M-H]-	Flavonoids	42971	62808	124159	146083	74057
164	3-Hydroxyphloretin-4'-O-glucoside	$C_{11}H_{14}N_2O_3$	[M-H]-	Flavonoids	45600	33308	26147	37930	49718
165	2,4'-Dihydroxy-4,6-dimethoxydihydrochalcone	$C_9H_{11}NO_3$	$[M+H]^+$	Flavonoids	56599	46328	127124	74083	46161
166	Phloretin-2'-O-glucoside (Phlorizin)	$C_{11}H_{14}N_2O_3$	[M-H]-	Flavonoids	20124	14991	22904	14843	17806
167	Phloretin-4'-O-glucoside (Trilobatin)	$C_9H_{11}NO_3$	[M-H]-	Flavonoids	13323	32329	56925	37907	40550
168	4-Methyl-5-thiazoleethanol	$C_{11}H_{14}N_2O_3$	$[M+H]^+$	Others	40321	81733	71336	112644	148269

169	2-Decanol*	$C_9H_{11}NO_3$	[M-H]-	Others	2160469	2215124	2423818	2381935	2582759
170	1-Decanol*	$C_{11}H_{14}N_2O_3$	[M-H]-	Others	2113319	2237420	2325021	2351538	2542459
171	Eucommiol	$C_9H_{11}NO_3$	[M-H]-	Others	1410078	1268129	964017	1290067	850155
172	1,2,4-Trihydroxyanthraquinone	$C_{11}H_{14}N_2O_3$	[M-H]-	Quinones	20189	32912	35617	35079	18088
173	5,6-Dihydroxylucidin	$C_9H_{11}NO_3$	[M-H]-	Quinones	29761	12643	163125	83132	34469
174	Pseudopurpurin(1,2,4-trihydroxy-3-carboxyanthraquinone)	$C_{11}H_{14}N_2O_3$	[M-H]-	Quinones	68016	20678	434046	244949	73329
175	Chrysophanol-9-anthrone	$C_9H_{11}NO_3$	[M-H]-	Quinones	1024613	1115608	995346	1266376	1847317
176	Emodin	$C_{11}H_{14}N_2O_3$	[M-H]-	Quinones	25147	10537	14299	13002	9
177	Butin; 7,3',4'-Trihydroxyflavanone*	$C_9H_{11}NO_3$	$[M+H]^+$	Flavonoids	1532827	14477130	19118338	13647536	16822232
178	Hesperetin-7-O-neohesperidoside(Neohesperidin)*	$C_{11}H_{14}N_2O_3$	$[M+H]^+$	Flavonoids	373633	339355	449864	396543	425078
179	Hesperetin-7-O-rutinoside (Hesperidin)*	$C_9H_{11}NO_3$	$[M+H]^+$	Flavonoids	275458	316885	411324	331409	402252
180	Hesperetin-7-O-glucoside	$C_{11}H_{14}N_2O_3$	$[M+H]^+$	Flavonoids	169691	296162	354510	322873	212233
181	Naringenin (5,7,4'-Trihydroxyflavanone)*	$C_9H_{11}NO_3$	$[M+H]^+$	Flavonoids	1486811	14557002	18568503	12560140	16668150
182	Eriodictyol (5,7,3',4'-Tetrahydroxyflavanone)	$C_{11}H_{14}N_2O_3$	$[M+H]^+$	Flavonoids	38278	86579	71515	68447	64308
183	Naringenin-7-O-Rutinoside-4'-O-glucoside	$C_9H_{11}NO_3$	[M-H]-	Flavonoids	37844	56952	58671	48362	41088
184	Hesperetin-7-O-(6"-malonyl)glucoside	$C_{11}H_{14}N_2O_3$	[M-H]-	Flavonoids	240146	462995	700944	276853	548554
185	Hesperetin	$C_9H_{11}NO_3$	$[M+H]^+$	Flavonoids	28366	73961	65659	59637	54222
186	Eriodictyol-7-O-Rutinoside (Eriocitrin)	$C_{11}H_{14}N_2O_3$	$[M+H]^+$	Flavonoids	75719	48731	39962	60858	86790

187	Hesperetin-5-O-glucoside	$C_9H_{11}NO_3$	[M-H]-	Flavonoids	1682961	1146743	1838608	2516929	1760621
188	Eriodictyol-7-O-(6"-malonyl)g lucoside	$C_{11}H_{14}N_2O_3$	$[M+H]^+$	Flavonoids	11490	15599	23984	7613	6276
189	Cirsilineol (4',5-Dihydroxy-3',6,7-trimeth oxyflavone)	$C_9H_{11}NO_3$	[M-H]-	Flavonoids	23002	24194	20375	25324	19656
190	Eriodictyol-3'-O-glucoside	$C_{11}H_{14}N_2O_3$	[M-H]-	Flavonoids	367590	1427006	1083332	700753	747292
191	Homoeriodictyol	$C_9H_{11}NO_3$	$[M+H]^+$	Flavonoids	48856	104090	90452	85966	88553
192	Naringenin-7-O-Neohesperido side(Naringin)	$C_{11}H_{14}N_2O_3$	[M-H]-	Flavonoids	58556	104838	105948	101074	129376
193	Naringenin-7-O-glucoside (Prunin)	$C_9H_{11}NO_3$	[M-H]-	Flavonoids	14077	13245	75481	101855	16961
194	Pinocembrin (Dihydrochrysin)	$C_{11}H_{14}N_2O_3$	$[M+H]^+$	Flavonoids	2800	11510	10790	9919	15804
195	Naringenin-4'-O-glucoside*	$C_9H_{11}NO_3$	[M-H]-	Flavonoids	23927	48423	45899	55632	58436
196	Naringenin-7-O-Rutinoside(Na rirutin)	$C_{11}H_{14}N_2O_3$	$[M+H]^+$	Flavonoids	24885	39409	40496	36010	50894
197	Butin-7-O-glucoside*	$C_9H_{11}NO_3$	[M-H]-	Flavonoids	20698	29938	36493	39309	41540
198	Eriodictyol-7-O-(6"-O-p-coum aroyl)glucoside	$C_{11}H_{14}N_2O_3$	[M-H]-	Flavonoids	4118	3986	3940	2166	9
199	3,5,7-Trihydroxyflavanone (Pinobanksin)*	$C_9H_{11}NO_3$	[M-H]-	Flavonoids	3129725	2621134	2659502	2242491	2071668
200	Taxifolin(Dihydroquercetin)	$C_{11}H_{14}N_2O_3$	[M-H]-	Flavonoids	68373	54756	51330	50790	48768
201	Aromadendrin (Dihydrokaempferol)	$C_9H_{11}NO_3$	[M-H]-	Flavonoids	14752	20223	17611	16988	21058
202	Taxifolin-3'-O-glucoside	$C_{11}H_{14}N_2O_3$	[M-H]-	Flavonoids	47677	37344	38670	70868	68690
203	Kaurenoic Acid	$C_9H_{11}NO_3$	[M-H]-	Terpenoids	167644	100918	69036	57989	93047
204	Pimaric acid	$C_{11}H_{14}N_2O_3$	[M-H]-	Terpenoids	169308	101972	69729	54924	92953
205	Ferruginol	$C_9H_{11}NO_3$	[M-H]-	Terpenoids	638307	196506	157627	274338	162554

206	12-Hydroxyabietic Acid	$C_{11}H_{14}N_2O_3$	$[M+H]^+$	Terpenoids	97281	25473	54166	56369	29043
207	3'-O-methylbatatasin III	$C_9H_{11}NO_3$	$[M+H]^+$	Quinones	3272830	6397296	4050160	3911487	3027115
208	2,5-dihydroxy-3,4-dimethoxy-9,10-dihydrophenanthrene	$C_{11}H_{14}N_2O_3$	[M-H]-	Quinones	678797	1377671	1615926	849479	1518664
209	Ephemeranthol A	$C_9H_{11}NO_3$	$[M+H]^+$	Quinones	224212	236447	180745	134736	99206
210	2,5-dihydroxy-3,4-dimeth-oxy phenanthrene	$C_{11}H_{14}N_2O_3$	[M-H]-	Quinones	13500	27526	27479	25456	17355
211	Denbinobin	$C_9H_{11}NO_3$	$[M+H]^+$	Quinones	1892340	3999108	3882995	3647770	2465487
212	Ephemeranthol B; Flavanthridin	$C_{11}H_{14}N_2O_3$	$[M+H]^+$	Quinones	1081520	2269966	2764414	1385494	2453732
213	4,6-dimethoxyphenanthrene-2,3,7-triol	$C_9H_{11}NO_3$	[M-H]-	Quinones	574421	1937216	1399384	885899	980861
214	4,7-Dihydroxy-2-methoxy-9,10-dihydrophenanthrene; Lusianthridin	$C_{11}H_{14}N_2O_3$	$[M+H]^+$	Quinones	42380	97948	9	62751	45477
215	Hircinol	$C_9H_{11}NO_3$	$[M+H]^+$	Quinones	200638	885876	709516	454017	342466
216	2,3-dihydroxy-7-methoxy-9,10-dihydro-phenanthrene	$C_{11}H_{14}N_2O_3$	[M-H]-	Quinones	54382	85014	106371	83522	71169
217	4-Methoxy-9,10-dihydrophenanthrene-2,5-diphenol	$C_9H_{11}NO_3$	[M-H]-	Quinones	652905	2072014	1313565	1103658	1194608
218	4,6-dimethoxy-9,10-dihydrophenanthrene-2,3,7-triol	$C_{11}H_{14}N_2O_3$	[M-H]-	Quinones	183262	276254	150195	190915	151538
219	3,4-dihydroxy-2,7-dimethoxyphenanthrene	$C_9H_{11}NO_3$	[M-H]-	Quinones	36957	74499	47170	28451	19015
220	1,4-dihydroxy-7-methoxy-9,10-dihydro-phenanthrene	$C_{11}H_{14}N_2O_3$	[M-H]-	Quinones	13281	31994	26980	19937	18552
221	2,7-dihydroxy-3,4-dimethoxy-9,10-dihydrophenanthrene	$C_9H_{11}NO_3$	[M-H]-	Quinones	707469	1456774	1697374	865094	1481785

222	2,5-dihydroxy-4-methoxy-9,10-dihydrophenanthrene	$C_{11}H_{14}N_2O_3$	[M-H]-	Quinones	89411	292563	194369	152121	178294
223	4-methoxy-9,10-dihydrophenanthrene-1,2,7-triol	$C_9H_{11}NO_3$	[M-H]-	Quinones	547880	421068	694124	283550	384237
224	1,7-dihydroxy-2,5-dimethoxy-9,10-dihydrophenanthrene	$C_{11}H_{14}N_2O_3$	[M-H]-	Quinones	3778483	8128156	9828099	5026458	8935960
225	7-hydroxy-4-methoxyphenanthrene-2-O-glucoside	$C_9H_{11}NO_3$	[M-H]-	Quinones	21155	249461	1153527	445996	867205
226	1-p-Hydroxybenzyl-4-methoxy-9,10-dihydrophenanthrene-2,7-diol	$C_{11}H_{14}N_2O_3$	$[M+H]^+$	Quinones	26249	48602	26211	25212	23169
227	9,10-Dihydro-2-methoxy-4,5-phenanthrenediol	$C_9H_{11}NO_3$	[M-H]-	Quinones	177360	389990	491626	322723	382094
228	Cannabidihydrophenanthrene	$C_{11}H_{14}N_2O_3$	[M-H]-	Quinones	5777	13978	15546	10667	13995
229	2,4,7-trihydroxy-9,10-dihydrophenanthrene	$C_9H_{11}NO_3$	[M-H]-	Quinones	118585	205871	393314	251274	454217
230	1-(3'-methoxy-4'-hydroxybenzyl)-2,7-dihydroxy-4-methoxyphenanthrene	$C_{11}H_{14}N_2O_3$	[M-H]-	Quinones	780568	135779	489314	447322	31274
231	2,4-dihydroxy-7-methoxy-9,10-dihydrophenanthrene	$C_9H_{11}NO_3$	[M-H]-	Quinones	15711	46996	31448	26375	27986
232	2,7-Dihydroxy-4-methoxy-9,10-dihydrophenanthrene; Coelonin	$C_{11}H_{14}N_2O_3$	[M-H]-	Quinones	141954	575552	508532	333020	254051
233	3,4-dihydroxy-7-methoxy-9,10-dihydro-phenanthrene	$C_9H_{11}NO_3$	[M-H]-	Quinones	20490	38888	50045	39131	28799
234	2,7-Dihydroxy-3,4,6-trimethoxyphenanthrene	$C_{11}H_{14}N_2O_3$	$[M+H]^+$	Quinones	24698	44712	42803	41305	38426

235	2,7-Dihydroxy-3,5-dimethoxy-9,10-dihydrophenanthrene	$C_9H_{11}NO_3$	[M-H]-	Quinones	270173	511144	494545	404695	333687
236	2,5-dihydroxyl-3,4,6-trimethoxy-9,10-dihydrophenanthrene	$C_{11}H_{14}N_2O_3$	[M-H]-	Quinones	12508	18696	32349	14597	20062
237	2,7-Dihydroxy-1-(p-hydroxybenzyl)-4-methoxy-9,10-dihydrophenanthrene-4'-O-glucose	$C_9H_{11}NO_3$	[M-H]-	Quinones	22905	31675	33772	29048	30057
238	7-Hydroxy-5,6-dimethoxy-1,4-phenanthrenequinone	$C_{11}H_{14}N_2O_3$	$[M+H]^+$	Quinones	10495	15480	12619	13386	9601
239	2,7-dihydroxy-4-methoxyphenanthrene	$C_9H_{11}NO_3$	[M-H]-	Quinones	9327	2252	21717	9	4336
240	7-hydroxy-2,4-dimethoxyphenanthrene	$C_{11}H_{14}N_2O_3$	[M-H]-	Quinones	21425	9337	8201	13754	3730
241	N-Feruloyltyramine	$C_9H_{11}NO_3$	$[M+H]^+$	Alkaloids	11744909	12869451	16510852	9765508	13924845
242	Vanillylamine	$C_{11}H_{14}N_2O_3$	$[M+H]^+$	Alkaloids	77646	188506	456213	128577	33142
243	p-Coumaroyltyramine*	$C_9H_{11}NO_3$	$[M+H]^+$	Alkaloids	4109145	2907860	3381059	1873108	2411023
244	Cinnamoyltyramine	$C_{11}H_{14}N_2O_3$	$[M+H]^+$	Alkaloids	1195498	1786756	1810230	1200408	1108528
245	N-(2-Hydroxy-4-methoxyphenyl)acetamide	$C_9H_{11}NO_3$	$[M+H]^+$	Alkaloids	24531194	14557267	19001657	22834133	10659454
246	N-trans-ferulicacidacylp-hydroxyphenylethylamine	$C_{11}H_{14}N_2O_3$	$[M+H]^+$	Alkaloids	10999970	12915418	16418932	9734995	13741952
247	4-Hydroxymandelonitrile	$C_9H_{11}NO_3$	$[M+H]^+$	Alkaloids	1122583	440140	718757	378743	69623
248	N-Cis-Feruloyl-3'-O-methyldopamine*	$C_{11}H_{14}N_2O_3$	$[M+H]^+$	Alkaloids	1688958	2695499	3240311	1963980	2394778
249	N-Cis-Feruloyltyramine	$C_9H_{11}NO_3$	$[M+H]^+$	Alkaloids	10860128	12417422	16511003	9959959	13456591
250	N-Trans-Feruloyl-3'-O-methyl dopamine	$C_{11}H_{14}N_2O_3$	$[M+H]^+$	Alkaloids	1664429	2552744	3303050	1974858	2368159
251	N-Feruloyl-3-methoxytyramin	$C_9H_{11}NO_3$	$[M+H]^+$	Alkaloids	1459037	2337740	3113300	1754416	2240237

	e*								
252	N-(4-O-(Glucosyl)-E-feruloyl)-tyramine	$C_{11}H_{14}N_2O_3$	[M-H]-	Alkaloids	155160	464915	850567	340990	375326
253	Dihydro-N-feruloyltyramine	$C_9H_{11}NO_3$	$[M+H]^+$	Alkaloids	70171	84509	95519	60320	78080
254	N',N'',N'''-p-Coumaroyl-cinnamoyl-caffeoyl spermidine	$C_{11}H_{14}N_2O_3$	$[M+H]^+$	Alkaloids	154853	108341	98158	145258	54043
255	Feruloylspermidine	$C_9H_{11}NO_3$	$[M+H]^+$	Alkaloids	30225	98858	68008	112202	9
256	Grossamide	$C_{11}H_{14}N_2O_3$	$[M+H]^+$	Alkaloids	367534	331268	417508	345394	394892
257	N-Feruloyloctopamine	$C_9H_{11}NO_3$	$[M+H]^+$	Alkaloids	197722	186890	372589	191776	225844
258	Dopamine	$C_{11}H_{14}N_2O_3$	$[M+H]^+$	Alkaloids	173917	1264995	2669079	468286	128064
259	N-Feruloylputrescine	$C_9H_{11}NO_3$	$[M+H]^+$	Alkaloids	373600	479518	598025	439099	212802
260	Dihydrocaffeoylspermine	$C_{11}H_{14}N_2O_3$	$[M+H]^+$	Alkaloids	10349	11632	19490	54980	140605
261	2-Aminophenol	$C_9H_{11}NO_3$	$[M+H]^+$	Alkaloids	19852	9	58351	55182	9
262	Sinapine	$C_{11}H_{14}N_2O_3$	$[M+H]^+$	Alkaloids	9	35327	18202	39107	45883
263	p-Coumaroylferuloylcadaverine	$C_9H_{11}NO_3$	$[M+H]^+$	Alkaloids	24550	23412	9	9	9
264	p-Coumaroylputrescine	$C_{11}H_{14}N_2O_3$	$[M+H]^+$	Alkaloids	31923	133200	114938	107913	58618
265	Cimicifugamide	$C_9H_{11}NO_3$	$[M+H]^+$	Alkaloids	18849	20485	23012	9	18006
266	Caffeoylspermine	$C_{11}H_{14}N_2O_3$	$[M+H]^+$	Alkaloids	10501	7783	12158	14098	10658
267	Butyl isobutyl phthalate*	$C_9H_{11}NO_3$	$[M+H]^+$	Phenolic acids	26289540	28762609	29233177	28844964	30811867
268	1-O-Sinapoyl-β-D-glucose	$C_{11}H_{14}N_2O_3$	[M-H]-	Phenolic acids	144716	418930	742441	438473	767012
269	Glucosyringic Acid	$C_9H_{11}NO_3$	[M-H]-	Phenolic acids	1590585	3774901	3204224	2890176	2159941
270	Diisobutyl phthalate*	$C_{11}H_{14}N_2O_3$	$[M+H]^+$	Phenolic acids	25874646	28910843	29620951	28899812	31479420
271	Tachioside	$C_9H_{11}NO_3$	[M-H]-	Phenolic acids	1266960	2048146	2380908	1788063	2145974
272	Isotachioside	$C_{11}H_{14}N_2O_3$	[M-H]-	Phenolic acids	828186	1302817	1527859	1132340	1611417
273	4-Nitrophenol	$C_9H_{11}NO_3$	$[M+H]^+$	Phenolic acids	29804216	53335467	46132730	43159914	40833374
274	4-Hydroxycinnamic acid p-hydroxyphenethylamine*	$C_{11}H_{14}N_2O_3$	$[M+H]^+$	Phenolic acids	3811179	2958770	3440089	1926518	2525185

275	Elemicin	$C_9H_{11}NO_3$	$[M+H]^+$	Phenolic acids	1458080	13206	32591	33832	9
276	Cryptochlorogenic acid (4-O-Caffeoylquinic acid)*	$C_{11}H_{14}N_2O_3$	[M-H]-	Phenolic acids	6039	40580	12633	41925	24535
277	(S)-2-Phenyloxirane	$C_9H_{11}NO_3$	$[M+H]^+$	Phenolic acids	14456910	24484671	31875970	30445741	11012283
278	4-O-Glucosyl-3,4-dihydroxybe nzyl alcohol	$C_{11}H_{14}N_2O_3$	[M-H]-	Phenolic acids	105220	89309	81969	86805	80830
279	1-O-(3,4-Dihydroxy-5-methox y-benzoyl)-glucoside	$C_9H_{11}NO_3$	[M-H]-	Phenolic acids	498704	132224	202005	190371	111464
280	Phthalic anhydride	$C_{11}H_{14}N_2O_3$	$[M+H]^+$	Phenolic acids	1334515	1466428	1574868	1450814	1537547
281	4-Methoxybenzaldehyde	$C_9H_{11}NO_3$	$[M+H]^+$	Phenolic acids	419481	790718	1060673	535301	754408
282	Coniferin	$C_{11}H_{14}N_2O_3$	[M-H]-	Phenolic acids	34714	49309	58114	50568	49475
283	1-O-Gentisoyl-β-D-glucoside*	$C_9H_{11}NO_3$	[M-H]-	Phenolic acids	1817467	2433976	2269067	1967162	1617339
284	Dehydrodiconiferyl alcohol-gamma'-O-glucoside	$C_{11}H_{14}N_2O_3$	$[M+H]^+$	Phenolic acids	1627022	1843309	2169152	1376023	1452007
285	Dimethyl phthalate	$C_9H_{11}NO_3$	$[M+H]^+$	Phenolic acids	101006	141134	100388	103647	106279
286	Coniferaldehyde	$C_{11}H_{14}N_2O_3$	$[M+H]^+$	Phenolic acids	123386	181113	183953	190912	162680
287	2,3-Dihydroxy-1-(4-hydroxy-3 ,5-dimethoxyphenyl)propan-1-one	$C_9H_{11}NO_3$	$[M+H]^+$	Phenolic acids	162720	782192	568144	625941	259670
288	1-O-p-Coumaroyl-β-D-glucose *	$C_{11}H_{14}N_2O_3$	[M-H]-	Phenolic acids	130728	111871	102590	101822	156536
289	1-Naphthol*	$C_9H_{11}NO_3$	[M-H]-	Phenolic acids	7202	6007	16047	11661	9912
290	Arbutin	$C_{11}H_{14}N_2O_3$	[M-H]-	Phenolic acids	45518	78542	73803	81557	84254
291	Salicylic acid-2-O-glucoside	$C_9H_{11}NO_3$	[M-H]-	Phenolic acids	1027021	1203134	904069	972601	976742
292	2-Acetyl-3-hydroxyphenyl-1-O -glucoside	$C_{11}H_{14}N_2O_3$	[M-H]-	Phenolic acids	137195	47693	71337	67600	44963
293	Dihydrocaffeoylglucose	$C_9H_{11}NO_3$	[M-H]-	Phenolic acids	9	83420	83575	84568	71590
294	3,4,5-Trimethoxycinnamic	$C_{11}H_{14}N_2O_3$	[M-H]-	Phenolic acids	195165	60397	79006	110190	104421

	acid								
295	6-O-Caffeoyl-D-glucose*	$C_9H_{11}NO_3$	[M-H]-	Phenolic acids	168362	165633	203617	147647	137108
296	3-Hydroxy-1-(4-hydroxy-3,5-dimethoxyphenyl)propan-1-one	$C_{11}H_{14}N_2O_3$	$[M+H]^+$	Phenolic acids	4114645	1722598	1054715	1515913	676921
297	Bis(2-ethylhexyl)phthalate*	$C_9H_{11}NO_3$	$[M+H]^+$	Phenolic acids	2141492	2940632	2536725	2700449	2839738
298	Syringaldehyde-4-O-glucoside	$C_{11}H_{14}N_2O_3$	[M-H]-	Phenolic acids	9	75982	85407	93413	85692
299	3,4'-Dihydroxy-3',5'-dimethoxypropiophenone	$C_9H_{11}NO_3$	$[M+H]^+$	Phenolic acids	3544593	1417695	927032	1437714	616229
300	Vanilloloside	$C_{11}H_{14}N_2O_3$	[M-H]-	Phenolic acids	1369789	1770661	1589442	1495440	1132741
301	1-O-Salicyloyl-β-D-glucose	$C_9H_{11}NO_3$	[M-H]-	Phenolic acids	305223	996667	910235	974658	502576
302	4'-Hydroxypropiophenone	$C_{11}H_{14}N_2O_3$	[M-H]-	Phenolic acids	21904	8111	12192	11229	11056
303	Ferulic acid*	$C_9H_{11}NO_3$	[M-H]-	Phenolic acids	1177316	1552069	2874573	1967933	2064006
304	Isoferulic Acid*	$C_{11}H_{14}N_2O_3$	[M-H]-	Phenolic acids	1177316	1552069	2874573	1967933	2064006
305	Phloroglucinol; 1,3,5-Benzenetriol	$C_9H_{11}NO_3$	$[M+H]^+$	Phenolic acids	183481	643927	506584	486167	10033911
306	2-Phenylethanol	$C_{11}H_{14}N_2O_3$	$[M-H2O+H]^+$	Phenolic acids	2440490	781943	914224	1261362	369829
307	Isoeugenol	$C_9H_{11}NO_3$	$[M+H]^+$	Phenolic acids	52378	84316	64054	45796	47626
308	3,4-dihydroxyphenylethanol-β-D-glucopyranoside	$C_{11}H_{14}N_2O_3$	[M-H]-	Phenolic acids	46340	47862	47620	41640	33000
309	Protocatechuic acid-4-O-glucoside*	$C_9H_{11}NO_3$	[M-H]-	Phenolic acids	1431256	1729687	1560993	1399843	1148919
310	(S)-2-Hydroxy-3-(4-Hydroxyphenyl)Propanoic Acid	$C_{11}H_{14}N_2O_3$	[M-H]-	Phenolic acids	74039	18304	421873	552640	9
311	3-Aminosalicylic acid	$C_9H_{11}NO_3$	[M-H]-	Phenolic acids	208434	328013	219911	271627	267383
312	2-Hydroxyphenol-1-O-glucosyl(6→1)rhamnoside	$C_{11}H_{14}N_2O_3$	[M-H]-	Phenolic acids	185069	193914	105818	162492	220227
313	Digalloylglucose	$C_9H_{11}NO_3$	[M-H]-	Phenolic acids	22598	27045	37114	24503	19729
314	3-(3,4,5-Trimethoxyphenyl)pro	$C_{11}H_{14}N_2O_3$	$[M+H]^+$	Phenolic acids	538200	921064	1722127	1247935	1831345

	pan-1-ol								
315	2,3-Di-O-Galloyl-β-D-Glucose	$C_9H_{11}NO_3$	[M-H]-	Phenolic acids	37820	56958	59456	61176	48733
316	Syringic acid	$C_{11}H_{14}N_2O_3$	[M-H]-	Phenolic acids	10982746	4499367	8057462	6506910	7937008
317	Diisooctyl Phthalate*	$C_9H_{11}NO_3$	$[M+H]^+$	Phenolic acids	5080164	6739797	6074528	6066409	6716733
318	Benzamide	$C_{11}H_{14}N_2O_3$	$[M+H]^+$	Phenolic acids	4027004	1032865	1262878	1862348	438506
319	3-Hydroxy-5-Methylphenol-1-O-Glucoside	$C_9H_{11}NO_3$	[M-H]-	Phenolic acids	58433	78728	85439	79083	60387
320	Sinapinaldehyde	$C_{11}H_{14}N_2O_3$	[M-H]-	Phenolic acids	1068069	2642660	3731358	2198663	3429071
321	Disinapoyl glucoside	$C_9H_{11}NO_3$	[M-H]-	Phenolic acids	14889	20465	29155	20401	28531
322	4-Hydroxy-3,5-diisopropylbenzaldehyde	$C_{11}H_{14}N_2O_3$	$[M+H]^+$	Phenolic acids	14852	32348	30465	33596	24766
323	Methyl 2,4-dihydroxyphenylacetate*	$C_9H_{11}NO_3$	[M-H]-	Phenolic acids	74217	20729	401219	507109	33083
324	Benzyl-(2''-O-glucosyl)glucoside*	$C_{11}H_{14}N_2O_3$	[M-H]-	Phenolic acids	18729	24786	33692	30096	29672
325	Methyl sinapate	$C_9H_{11}NO_3$	[M-H]-	Phenolic acids	11992	6645	12546	5097	7718
326	β-Oxoacteoside	$C_{11}H_{14}N_2O_3$	[M-H]-	Phenolic acids	14495	22347	21307	25078	28894
327	Sinapic acid	$C_9H_{11}NO_3$	[M-H]-	Phenolic acids	195057	221763	1735315	417321	1321759
328	Isovanillin	$C_{11}H_{14}N_2O_3$	$[M+H]^+$	Phenolic acids	256661	1158971	827666	950681	1189674
329	Coniferyl alcohol	$C_9H_{11}NO_3$	[M-H]-	Phenolic acids	116985	357369	1173778	303627	829048
330	Dihydroxybenzoyl xyloside	$C_{11}H_{14}N_2O_3$	[M-H]-	Phenolic acids	263009	157636	209108	258544	154065
331	Caffeic acid	$C_9H_{11}NO_3$	[M-H]-	Phenolic acids	166275	400250	655716	567593	237135
332	Chlorogenic acid (3-O-Caffeoylquinic acid)*	$C_{11}H_{14}N_2O_3$	[M-H]-	Phenolic acids	13105	67495	29716	64350	26586
333	Vanillin acetate	$C_9H_{11}NO_3$	$[M+H]^+$	Phenolic acids	239037	78005	66270	75284	48650
334	Ethylparaben	$C_{11}H_{14}N_2O_3$	[M-H]-	Phenolic acids	300833	4385	17258	23842	9
335	3-hydroxyphenylacetic acid*	$C_9H_{11}NO_3$	[M-H]-	Phenolic acids	19820	14660	40602	32838	16532
336	Hydroxytyrosol	$C_{11}H_{14}N_2O_3$	[M-H]-	Phenolic acids	26402	17159	400418	123385	9

337	2,5-Dihydroxybenzaldehyde Methyl	$C_9H_{11}NO_3$	[M-H]-	Phenolic acids	6932213	3719124	4872380	6059787	2957694
338	3-(3-hydroxy-4-methoxyphenyl)propanoate*	$C_{11}H_{14}N_2O_3$	[M-H]-	Phenolic acids	125974	109068	310932	119220	419613
339	Trans-4-Hydroxycinnamic Acid Methyl Ester	$C_9H_{11}NO_3$	[M-H]-	Phenolic acids	64527	79120	67281	73982	40025
340	p-Coumaryl alcohol	$C_{11}H_{14}N_2O_3$	[M-H]-	Phenolic acids	7324	16771	17903	9958	20407
341	Ethyl ferulate	$C_9H_{11}NO_3$	[M-H]-	Phenolic acids	222655	24759	86989	91920	23913
342	Salicyl Alcohol	$C_{11}H_{14}N_2O_3$	[M-H]-	Phenolic acids	27795	36809	60970	68050	29409
343	3,4-Dihydroxybenzoic acid (Protocatechuic acid)*	$C_9H_{11}NO_3$	[M-H]-	Phenolic acids	10233772	4825598	12994744	10958969	7472841
344	Antiarol; 3,4,5-Trimethoxyphenol	$C_{11}H_{14}N_2O_3$	$[M+H]^+$	Phenolic acids	171679	50452	134075	149575	63208
345	1-O-Feruloyl-β-D-glucose	$C_9H_{11}NO_3$	[M-H]-	Phenolic acids	11699	142858	111284	78229	103722
346	4-Hydroxybenzoic acid	$C_{11}H_{14}N_2O_3$	[M-H]-	Phenolic acids	10929286	5909010	8090671	9798573	4696424
347	3,4-Dihydroxybenzoic Acid Ethyl Ester (Protocatechuic acid ethyl ester)	$C_9H_{11}NO_3$	[M-H]-	Phenolic acids	519621	9	33565	42307	9
348	α-Hydroxycinnamic Acid*	$C_{11}H_{14}N_2O_3$	[M-H]-	Phenolic acids	250701	399651	712027	671067	867807
349	2,6-Di-tert-butylphenol*	$C_9H_{11}NO_3$	[M-H]-	Phenolic acids	164368	198026	200869	186924	208564
350	Ferulic acid methyl ester	$C_{11}H_{14}N_2O_3$	[M-H]-	Phenolic acids	33176	47246	32006	46563	11419
351	2-Naphthol*	$C_9H_{11}NO_3$	[M-H]-	Phenolic acids	8452	8203	18337	12630	10149
352	2-(Formylamino)benzoic acid	$C_{11}H_{14}N_2O_3$	[M-H]-	Phenolic acids	496818	1229832	1337622	1214657	1585674
353	2,3-Dihydroxybenzoic Acid*	$C_9H_{11}NO_3$	[M-H]-	Phenolic acids	9105864	4309971	11775401	10107058	7020167
354	1-O-Vanilloyl-D-Glucose	$C_{11}H_{14}N_2O_3$	[M-H]-	Phenolic acids	39721	9	16944	12572	9
355	2-Amino-3-methoxybenzoic acid	$C_9H_{11}NO_3$	$[M+H]^+$	Phenolic acids	115619	63391	60359	58670	105319
356	Gallic acid-4-O-glucoside	$C_{11}H_{14}N_2O_3$	[M-H]-	Phenolic acids	44213	16945	34831	26663	23431

357	4-Hydroxyphenylacetic acid*	$C_9H_{11}NO_3$	[M-H]-	Phenolic acids	23585	17698	51581	46434	26433
358	Tyrosol; 4-Hydroxyphenylethanol	$C_{11}H_{14}N_2O_3$	[M-H]-	Phenolic acids	65222	16648	71326	58389	29118
359	Caffeic aldehyde	$C_9H_{11}NO_3$	$[M+H]^+$	Phenolic acids	171374	350417	347349	328176	428298
360	Gallic acid	$C_{11}H_{14}N_2O_3$	[M-H]-	Phenolic acids	253491	197114	684595	1521339	503946
361	2,5-Dihydroxybenzoic acid; Gentisic Acid*	$C_9H_{11}NO_3$	[M-H]-	Phenolic acids	4178242	2025337	5442725	4745255	3195617
362	1-O-Caffeoyl-β-D-glucose*	$C_{11}H_{14}N_2O_3$	[M-H]-	Phenolic acids	197457	165987	187959	138641	128938
363	Vanillic acid methyl ester	$C_9H_{11}NO_3$	[M-H]-	Phenolic acids	679936	35560	33976	36120	9157
364	Vanillic acid-4-O-glucoside	$C_{11}H_{14}N_2O_3$	[M-H]-	Phenolic acids	9	43049	49142	65639	43509
365	3-Methyl-4,8-dihydroxy-3,4-dihydroisocoumarin	$C_9H_{11}NO_3$	[M-H]-	Phenolic acids	76624	44400	220312	122996	9
366	Benzyl-(2"-O-xylosyl)glucoside	$C_{11}H_{14}N_2O_3$	[M-H]-	Phenolic acids	534116	629799	370398	507155	535873
367	Vanillin; 4-Hydroxy-3-Methoxybenzaldehyde*	$C_9H_{11}NO_3$	[M-H]-	Phenolic acids	2243830	10568435	9093065	9944159	13021884
368	p-Coumaric acid	$C_{11}H_{14}N_2O_3$	$[M+H]^+$	Phenolic acids	97539	244373	219113	207908	269252
369	Methyl Syringate	$C_9H_{11}NO_3$	[M-H]-	Phenolic acids	78510	49923	31771	37445	14611
370	4-Hydroxybenzaldehyde	$C_{11}H_{14}N_2O_3$	[M-H]-	Phenolic acids	3302999	7063659	6193319	7442008	10671741
371	4-Hydroxyphenyllactic Acid*	$C_9H_{11}NO_3$	[M-H]-	Phenolic acids	80089	25167	498166	628038	33857
372	4-Hydroxyacetophenone	$C_{11}H_{14}N_2O_3$	[M-H]-	Phenolic acids	112681	154765	186589	189326	273212
373	Mucic acid Dimethyl Ester	$C_9H_{11}NO_3$	[M-H]-	Phenolic acids	5028229	4275716	3135598	2885608	1453988
374	Salicylic acid	$C_{11}H_{14}N_2O_3$	[M-H]-	Phenolic acids	103414	121605	223313	154687	204623
375	3-O-Methylgallic acid	$C_9H_{11}NO_3$	[M-H]-	Phenolic acids	221830	40103	205964	135965	58300
376	Sinapyl alcohol*	$C_{11}H_{14}N_2O_3$	[M-H]-	Phenolic acids	125022	125857	307547	117762	422771
377	2,4-Di-Tert-Butylphenol*	$C_9H_{11}NO_3$	[M-H]-	Phenolic acids	142506	181876	180940	169474	192214
378	3-Methoxybenzoic acid	$C_{11}H_{14}N_2O_3$	[M-H]-	Phenolic acids	261437	444960	6651946	10032047	624581

379	2,3-Dimethoxybenzaldehyde	$C_9H_{11}NO_3$	$[M+H]^+$	Phenolic acids	65955	94575	81034	62534	104840
380	3-Hydroxycinnamic Acid*	$C_{11}H_{14}N_2O_3$	[M-H]-	Phenolic acids	360072	459743	755376	704391	991705
381	1-O-Cinnamoyl-4,6-(S)-HHDP -β-D-glucose	$C_9H_{11}NO_3$	$[M+H]^+$	Phenolic acids	47736	32306	32333	48593	44690
382	Vanillic acid	$C_{11}H_{14}N_2O_3$	[M-H]-	Phenolic acids	3362195	1814221	2095108	2433127	1086298
383	1,6-Di-O-caffeoyl-β-D-glucose	$C_9H_{11}NO_3$	[M-H]-	Phenolic acids	1099900	500879	918052	994146	719630
384	4-O-Methylgallic Acid	$C_{11}H_{14}N_2O_3$	[M-H]-	Phenolic acids	148350	60632	102535	78116	64102
385	5-Acetylsalicylic acid	$C_9H_{11}NO_3$	$[M+H]^+$	Phenolic acids	58263	28979	40458	45963	33809
386	4,5-O-Dicaffeoylquinic Acid Methyl Ester	$C_{11}H_{14}N_2O_3$	[M-H]-	Phenolic acids	12682	13199	7124	9552	17943
387	Syringaldehyde; 4-Hydroxy-3,5-Dimethoxyben zaldehyde	$C_9H_{11}NO_3$	[M-H]-	Phenolic acids	6691049	5893406	3940524	6431265	5123464
388	Methyl 4-hydroxybenzoate*	$C_{11}H_{14}N_2O_3$	[M-H]-	Phenolic acids	398052	1920155	1580652	1863961	2267792
389	Protocatechuic Acid Methyl Ester	$C_9H_{11}NO_3$	[M-H]-	Phenolic acids	546741	136963	265845	266792	62449
390	Eudesmic acid (3,4,5-trimethoxybenzoic acid)	$C_{11}H_{14}N_2O_3$	$[M+H]^+$	Phenolic acids	111371	14346	15714	21716	15945
391	Gallacetophenone	$C_9H_{11}NO_3$	$[M+H]^+$	Phenolic acids	9	110650	144802	171546	132722
392	Cinnamic acid	$C_{11}H_{14}N_2O_3$	[M-H]-	Phenolic acids	3932	25301	18740	20498	24802
393	p-Coumaroylcaffeoyltartaric acid	$C_9H_{11}NO_3$	[M-H]-	Phenolic acids	34028	46701	23204	34574	26234
394	O-Anisic acid (2-Methoxybenzoic acid)*	$C_{11}H_{14}N_2O_3$	[M-H]-	Phenolic acids	10868	7125	7977	7539	70136
395	Phenoxyacetic acid*	$C_9H_{11}NO_3$	[M-H]-	Phenolic acids	10868	7125	7977	7539	70136
396	p-Coumaroylmalic acid	$C_{11}H_{14}N_2O_3$	[M-H]-	Phenolic acids	4863	90043	75503	34478	59967
397	3-(4-Hydroxyphenyl)-1-propan ol	$C_9H_{11}NO_3$	[M-H]-	Phenolic acids	130357	9680	4200	6344	2595

398	Methyl gallate	$C_{11}H_{14}N_2O_3$	[M-H]-	Phenolic acids	41069	70674	171387	110731	56694
399	Phenyl acetate	$C_9H_{11}NO_3$	[M-H]-	Phenolic acids	123364	162963	221212	230596	309351
400	4-Methoxyphenylpropionic acid	$C_{11}H_{14}N_2O_3$	[M-H]-	Phenolic acids	34187	105515	328770	82186	232098
401	2-Hydroxycinnamic acid*	$C_9H_{11}NO_3$	[M-H]-	Phenolic acids	347456	560656	941203	839571	1080567
402	p-Coumaric acid-4-O-glucoside*	$C_{11}H_{14}N_2O_3$	[M-H]-	Phenolic acids	51683	33956	34068	35811	40104
403	Dihydroferulic Acid	$C_9H_{11}NO_3$	[M-H]-	Phenolic acids	59227	119033	157751	121568	101076
404	Propyl 4-hydroxybenzoate	$C_{11}H_{14}N_2O_3$	[M-H]-	Phenolic acids	11872	7498	9068	8940	12750
405	10-hydroxymajoroside	$C_9H_{11}NO_3$	[M-H]-	Phenolic acids	53966	39769	62166	155238	63034
406	3,4,5-Tricaffeoylquinic acid	$C_{11}H_{14}N_2O_3$	[M-H]-	Phenolic acids	27419	49219	29885	34166	18896
407	3,4-Dimethoxyphenyl acetic acid	$C_9H_{11}NO_3$	[M-H]-	Phenolic acids	21407	18574	39911	23212	19628
408	Ethyl Rosmarinate	$C_{11}H_{14}N_2O_3$	$[M+H]^+$	Phenolic acids	16438	13382	11004	10530	14287
409	3,4-Di-O-caffeoylquinic acid methyl ester	$C_9H_{11}NO_3$	$[M+H]^+$	Phenolic acids	23199	27709	22837	17600	18931
410	3-(3-Hydroxyphenyl)-3-hydroxypropanoic acid	$C_{11}H_{14}N_2O_3$	[M-H]-	Phenolic acids	51942	33390	36493	44611	18572
411	2-hydroxymethyl benzoic acid	$C_9H_{11}NO_3$	[M-H]-	Phenolic acids	10341	10050	24267	15459	6560
412	2-Hydroxy-3-phenylpropanoic acid	$C_{11}H_{14}N_2O_3$	[M-H]-	Phenolic acids	189265	74063	961835	851519	64956
413	Anthranilate-1-O-Sophoroside	$C_9H_{11}NO_3$	[M-H]-	Phenolic acids	723838	661377	923177	1163281	703294
414	2,6-Dimethoxybenzaldehyde*	$C_{11}H_{14}N_2O_3$	[M-H]-	Phenolic acids	196758	111590	948271	820780	128315
415	Maleoyl-caffeoylquinic acid	$C_9H_{11}NO_3$	[M-H]-	Phenolic acids	44728	45803	45376	46658	27798
416	Benzyl-β-gentiobioside*	$C_{11}H_{14}N_2O_3$	[M-H]-	Phenolic acids	17754	27902	30163	32050	24339
417	2-O-Galloyl-D-glucose	$C_9H_{11}NO_3$	[M-H]-	Phenolic acids	9	541864	660293	394327	512603
418	4-Methylphenol	$C_{11}H_{14}N_2O_3$	[M-H]-	Phenolic acids	73028	3324	3046	5192	1880
419	Methyl	$C_9H_{11}NO_3$	[M-H]-	Phenolic acids	9268	13908	18269	12868	9

	3-(4-hydroxyphenyl)propionate								
420	1-(4-Methoxyphenyl)-1-propanol	$C_{11}H_{14}N_2O_3$	[M-H]-	Phenolic acids	175836	235859	211272	274600	250181
421	4-(3,4,5-Trihydroxybenzoxy)benzoic acid	$C_9H_{11}NO_3$	[M-H]-	Phenolic acids	32512	18943	19652	22096	12530
422	Methyl caffeate	$C_{11}H_{14}N_2O_3$	[M-H]-	Phenolic acids	1060416	3296663	2050132	2357285	1406816
423	p-Coumaric acid ethyl ester	$C_9H_{11}NO_3$	$[M+H]^+$	Phenolic acids	32923	9	9558	12173	9
424	p-Coumaraldehyde	$C_{11}H_{14}N_2O_3$	[M-H]-	Phenolic acids	5900	9282	8792	11017	6031
425	1-Linoleoylglycerol	$C_9H_{11}NO_3$	$[M+H]^+$	Lipids	448084	218366	349163	382213	157271
426	2-Linoleoylglycerol-1-O-glucoside	$C_{11}H_{14}N_2O_3$	$[M+H]^+$	Lipids	52203	49060	166084	197518	10347
427	1-α-Linolenoyl-glycerol	$C_9H_{11}NO_3$	$[M+H]^+$	Lipids	687082	180768	328139	348434	118286
428	1-α-Linolenoyl-glycerol-2,3-di-O-glucoside	$C_{11}H_{14}N_2O_3$	$[M+H]^+$	Lipids	9	10106	38488	38864	11841
429	2-Linoleoylglycerol	$C_9H_{11}NO_3$	$[M+H]^+$	Lipids	269688	258944	881819	1048264	79677
430	1-Oleoyl-Sn-Glycerol	$C_{11}H_{14}N_2O_3$	$[M+H]^+$	Lipids	9	90659	439649	591301	30177
431	Monopalmitin	$C_9H_{11}NO_3$	$[M+H]^+$	Lipids	261241	205273	204860	165822	233608
432	LysoPG 16:0	$C_{11}H_{14}N_2O_3$	[M-H]-	Lipids	48707	979800	3672012	5531398	720426
433	1-α-Linolenoyl-glycerol-3-O-glucoside	$C_9H_{11}NO_3$	$[M+H]^+$	Lipids	33768	33616	69497	76887	33127
434	2-α-Linolenoyl-glycerol-1,3-di-O-glucoside	$C_{11}H_{14}N_2O_3$	$[M+H]^+$	Lipids	9	46339	190288	238130	13856
435	LysoPI 18:3	$C_9H_{11}NO_3$	[M-H]-	Lipids	1822	126249	847606	851298	59761
436	2-Linoleoylglycerol-1,3-di-O-glucoside	$C_{11}H_{14}N_2O_3$	$[M+H]^+$	Lipids	9	70564	274690	302337	15653
437	1-Linoleoylglycerol-3-O-glucoside	$C_9H_{11}NO_3$	$[M+H]^+$	Lipids	56216	27300	59303	56827	18683

438	2-α-Linolenoyl-glycerol-1-O-glucoside	$C_{11}H_{14}N_2O_3$	$[M+H]^+$	Lipids	36541	61670	143042	234471	14747
439	2-α-Linolenoyl-glycerol	$C_9H_{11}NO_3$	$[M+H]^+$	Lipids	224168	161888	843438	960462	49216
440	1-Linoleoylglycerol-2,3-di-O-glucoside	$C_{11}H_{14}N_2O_3$	$[M+H]^+$	Lipids	9	13352	40510	45340	12261
441	Xanthine	$C_9H_{11}NO_3$	[M-H]-	Nucleotides and derivatives	950566	94127	647187	682245	70338
442	Inosine	$C_{11}H_{14}N_2O_3$	$[M+H]^+$	Nucleotides and derivatives	371470	841800	826155	831683	590023
443	N7-Methylguanosine	$C_9H_{11}NO_3$	$[M+H]^+$	Nucleotides and derivatives	210100	621079	527603	705553	131354
444	Adenosine	$C_{11}H_{14}N_2O_3$	$[M+H]^+$	Nucleotides and derivatives	2426337	6060508	5360156	7182351	5694521
445	5-Methyluridine	$C_9H_{11}NO_3$	[M-H]-	Nucleotides and derivatives	74918	56902	52654	71508	77741
446	2'-O-Methyladenosine	$C_{11}H_{14}N_2O_3$	$[M+H]^+$	Nucleotides and derivatives	3076925	2499128	2716933	4945672	1353035
447	Thymidine	$C_9H_{11}NO_3$	$[M+H]^+$	Nucleotides and derivatives	6114262	8512329	5699676	9453529	4023859
448	2-Deoxyribose-1-phosphate	$C_{11}H_{14}N_2O_3$	[M-H]-	Nucleotides and derivatives	6328826	539452	6024535	12149325	302732
449	2'-Deoxyinosine-5'-monophosphate	$C_9H_{11}NO_3$	$[M+H]^+$	Nucleotides and derivatives	1485364	1461697	813469	391249	739655
450	2-(Dimethylamino)guanosine	$C_{11}H_{14}N_2O_3$	$[M+H]^+$	Nucleotides and derivatives	2030009	1495027	1797904	3270018	879929
451	2'-Deoxycytidine	$C_9H_{11}NO_3$	$[M+H]^+$	Nucleotides and derivatives	1744376	5672229	2739360	4517963	1818115
452	Uridine	$C_{11}H_{14}N_2O_3$	[M-H]-	Nucleotides and	2171346	3844744	1638156	2217430	1998235

				derivatives					
453	Guanine	$C_9H_{11}NO_3$	$[M+H]^+$	Nucleotides and derivatives	5716232	8684616	4338641	4192590	438090
454	2'-Deoxyguanosine	$C_{11}H_{14}N_2O_3$	$[M+H]^+$	Nucleotides and derivatives	4451711	13119724	8873926	14808246	5301587
455	2-Deoxyribose-5'-phosphate	$C_9H_{11}NO_3$	[M-H]-	Nucleotides and derivatives	360460	35426	296263	363898	14914
456	Guanosine 3',5'-cyclic monophosphate	$C_{11}H_{14}N_2O_3$	[M-H]-	Nucleotides and derivatives	300335	3941855	3353093	2215893	6321024
457	2'-Deoxyinosine	$C_9H_{11}NO_3$	$[M+H]^+$	Nucleotides and derivatives	104090	241555	282876	483156	9
458	Riboflavin 5'-Adenosine Diphosphate	$C_{11}H_{14}N_2O_3$	[M-H]-	Nucleotides and derivatives	51702	11088	8623	13219	11867
459	5-Aminoimidazole ribonucleotide	$C_9H_{11}NO_3$	$[M+H]^+$	Nucleotides and derivatives	45576	4031875	5121468	5596949	1506892
460	Hypoxanthine	$C_{11}H_{14}N_2O_3$	$[M+H]^+$	Nucleotides and derivatives	1220120	287220	652457	447640	27690
461	Vidarabine	$C_9H_{11}NO_3$	$[M+H]^+$	Nucleotides and derivatives	2472369	5953245	5574395	6612119	5630001
462	5'-Deoxy-5'-(methylthio)adenosine	$C_{11}H_{14}N_2O_3$	$[M+H]^+$	Nucleotides and derivatives	2138981	641736	2493164	1395159	842331
463	Cytarabine	$C_9H_{11}NO_3$	$[M+H]^+$	Nucleotides and derivatives	7336664	26175109	15165352	21523634	9577772
464	Cytidine 5'-monophosphate(Cytidylic acid)	$C_{11}H_{14}N_2O_3$	$[M+H]^+$	Nucleotides and derivatives	51189	415452	607294	666684	214360
465	5-Methylcytosine	$C_9H_{11}NO_3$	$[M+H]^+$	Nucleotides and derivatives	191123	401698	261576	464430	150763

466	1-beta-D-Arabinofuranosyluracil	$C_{11}H_{14}N_2O_3$	$[M+H]^+$	Nucleotides and derivatives	2010148	4220243	2659538	2868612	1528813
467	8-Hydroxyguanosine	$C_9H_{11}NO_3$	[M-H]-	Nucleotides and derivatives	17107	32756	56616	82004	9
468	Isoguanine	$C_{11}H_{14}N_2O_3$	$[M+H]^+$	Nucleotides and derivatives	1266934	2262758	1190280	1245755	233918
469	2'-Deoxyadenosine	$C_9H_{11}NO_3$	$[M+H]^+$	Nucleotides and derivatives	715391	2392983	1640996	3009952	1685596
470	2-Aminopurine	$C_{11}H_{14}N_2O_3$	$[M+H]^+$	Nucleotides and derivatives	595922	1544471	1231658	1746666	1129249
471	9-(Arabinosyl)hypoxanthine	$C_9H_{11}NO_3$	[M-H]-	Nucleotides and derivatives	683150	1366853	872485	1395229	305183
472	Adenine	$C_{11}H_{14}N_2O_3$	$[M+H]^+$	Nucleotides and derivatives	18589251	25439420	11204931	15439381	2967635
473	5-Methyl-2'-Deoxycytidine	$C_9H_{11}NO_3$	$[M+H]^+$	Nucleotides and derivatives	1703689	3551809	2660289	4078003	1413383
474	2'-Deoxyuridine	$C_{11}H_{14}N_2O_3$	[M-H]-	Nucleotides and derivatives	82176	15187	32631	66826	9344
475	6-O-methylguanine	$C_9H_{11}NO_3$	$[M+H]^+$	Nucleotides and derivatives	219488	134429	149395	163863	54431
476	β-Pseudouridine	$C_{11}H_{14}N_2O_3$	[M-H]-	Nucleotides and derivatives	348413	263784	263128	338428	213679
477	Isocytosine	$C_9H_{11}NO_3$	$[M+H]^+$	Nucleotides and derivatives	76163	64655	83472	137560	129757
478	Succinyladenosine	$C_{11}H_{14}N_2O_3$	$[M+H]^+$	Nucleotides and derivatives	3622002	662188	1639401	2616313	514717
479	N6-methyladenosine	$C_9H_{11}NO_3$	$[M+H]^+$	Nucleotides and derivatives	509612	1929552	1757278	2047595	2007363

480	8-Hydroxy-2-deoxyguanosine	$C_{11}H_{14}N_2O_3$	$[M+H]^+$	Nucleotides and derivatives	9254	11384	19750	35333	9
481	Xanthosine	$C_9H_{11}NO_3$	[M-H]-	Nucleotides and derivatives	1109939	695168	435447	694296	904291
482	Guanosine	$C_{11}H_{14}N_2O_3$	$[M+H]^+$	Nucleotides and derivatives	2372817	8298928	7010890	8789625	5173419
483	Guanosine 5'-monophosphate	$C_9H_{11}NO_3$	$[M+H]^+$	Nucleotides and derivatives	25453	411705	459231	418538	197716
484	8-Azaguanine	$C_{11}H_{14}N_2O_3$	$[M+H]^+$	Nucleotides and derivatives	1020912	741985	685372	594389	50085
485	Riboprine	$C_9H_{11}NO_3$	$[M+H]^+$	Nucleotides and derivatives	19518	24646	34633	39267	37587
486	Allopurinol	$C_{11}H_{14}N_2O_3$	$[M+H]^+$	Nucleotides and derivatives	1370904	289971	722025	495131	25491
487	Uridine-5'-Diphosphate-D-Xylose	$C_9H_{11}NO_3$	[M-H]-	Nucleotides and derivatives	4377	84242	60442	45042	19973
488	Cytosine	$C_{11}H_{14}N_2O_3$	$[M+H]^+$	Nucleotides and derivatives	1044471	5035435	2741628	3897709	1833203
489	Uridine 5'-diphospho-D-glucose	$C_9H_{11}NO_3$	[M-H]-	Nucleotides and derivatives	117377	964950	733635	602446	403369
490	Ribosyladenosine	$C_{11}H_{14}N_2O_3$	$[M+H]^+$	Nucleotides and derivatives	138641	135129	141551	135479	94735
491	Uridine 5'-diphospho-N-acetylglucosamine	$C_9H_{11}NO_3$	[M-H]-	Nucleotides and derivatives	76962	40010	53141	72617	51528
492	Uridine 5'-monophosphate	$C_{11}H_{14}N_2O_3$	[M-H]-	Nucleotides and derivatives	34230	298915	2921292	4379421	454333
493	Thymine	$C_9H_{11}NO_3$	$[M+H]^+$	Nucleotides and	139421	16837	196755	565873	20093

				derivatives					
494	Cyclic 3',5'-Adenylic acid	$C_{11}H_{14}N_2O_3$	[M-H]-	Nucleotides and derivatives	254414	759497	619729	716541	693070
495	Flavin Single Nucleotide(FMN)	$C_9H_{11}NO_3$	[M-H]-	Nucleotides and derivatives	34827	142748	115862	118262	64155
496	Cytidine	$C_{11}H_{14}N_2O_3$	$[M+H]^+$	Nucleotides and derivatives	10217531	42801103	25627985	32049594	16182329
497	Adenosine 5'-monophosphate	$C_9H_{11}NO_3$	$[M+H]^+$	Nucleotides and derivatives	16478	531642	747442	231566	806878
498	1-Methylxanthine	$C_{11}H_{14}N_2O_3$	$[M+H]^+$	Nucleotides and derivatives	25559	9	5967	13967	9
499	Adenosine 5'-diphosphate	$C_9H_{11}NO_3$	[M-H]-	Nucleotides and derivatives	2657	11109	24152	11092	48262
500	1-Methyladenosine	$C_{11}H_{14}N_2O_3$	[M-H]-	Nucleotides and derivatives	9	33355	27527	40139	45790
501	β-Nicotinamide mononucleotide	$C_9H_{11}NO_3$	$[M+H]^+$	Nucleotides and derivatives	9	27070	57744	23153	9
502	Uridine-5'-diphosphoglucuroni c acid	$C_{11}H_{14}N_2O_3$	[M-H]-	Nucleotides and derivatives	9	7125	8093	7055	4485
503	4'-O-Glucosylvitexin	$C_9H_{11}NO_3$	$[M+H]^+$	Flavonoids	49092	735498	9	144463	655805
504	3,5,6,7,8,4'-Hexamethoxyflavo ne	$C_{11}H_{14}N_2O_3$	$[M+H]^+$	Flavonoids	127707	285270	197940	281335	757014
505	Tricin (5,7,4'-Trihydroxy-3',5'-dimeth oxyflavone)	$C_9H_{11}NO_3$	$[M+H]^+$	Flavonoids	265353	205353	227225	244531	214673
506	Hispidulin (5,7,4'-Trihydroxy-6-methoxyf lavone)*	$C_{11}H_{14}N_2O_3$	$[M+H]^+$	Flavonoids	47732	81673	55824	53954	73951

507	5,7,8,4'-Tetramethoxyflavone	$C_9H_{11}NO_3$	$[M+H]^+$	Flavonoids	9073	22978	11207	20310	49481
508	Hispidulin-7-O-glucoside(Hom oplantaginin)*	$C_{11}H_{14}N_2O_3$	$[M+H]^+$	Flavonoids	950860	1222578	936352	911674	897280
509	Apigenin-5-O-glucoside	$C_9H_{11}NO_3$	$[M+H]^+$	Flavonoids	38374	38621	41519	37259	29842
510	5-Hydroxy-3,7,3',4'-tetramethoxyflavone (Retusin)	$C_{11}H_{14}N_2O_3$	$[M+H]^+$	Flavonoids	13359	6750	4844	3655	12360
511	Vitexin-2''-O-glucoside	$C_9H_{11}NO_3$	$[M+H]^+$	Flavonoids	4481498	4340175	3250809	3761823	2857024
512	Diosmetin-7-O-galactoside*	$C_{11}H_{14}N_2O_3$	$[M+H]^+$	Flavonoids	809694	1012675	727283	777358	811284
513	Isovitexin-2''-O-rhamnoside	$C_9H_{11}NO_3$	$[M+H]^+$	Flavonoids	438593	627979	524273	403823	421244
514	Orientin-7-O-glucoside	$C_{11}H_{14}N_2O_3$	$[M+H]^+$	Flavonoids	2077003	1298667	1592032	1781654	1873561
515	Acacetin	$C_9H_{11}NO_3$	$[M+H]^+$	Flavonoids	1932118	3989911	3665863	3767308	2399573
516	Isosaponarin(Isovitexin-4'-O-glucoside)	$C_{11}H_{14}N_2O_3$	$[M+H]^+$	Flavonoids	22225	319098	23208	56498	301007
517	Luteolin-6-C-(5''-glucuronyl)xyloside	$C_9H_{11}NO_3$	$[M+H]^+$	Flavonoids	213752	211056	194386	219274	204266
518	Chrysoeriol-6,8-di-C-glucoside	$C_{11}H_{14}N_2O_3$	$[M+H]^+$	Flavonoids	1232054	1443484	1103161	1009460	877054
519	3,5,7,2'-Tetrahydroxyflavone; Datiscetin	$C_9H_{11}NO_3$	[M-H]-	Flavonoids	24215	81773	63202	42077	44901
520	Luteolin-7-O-neohesperidoside (Lonicerin)*	$C_{11}H_{14}N_2O_3$	$[M+H]^+$	Flavonoids	3703597	1879224	1518329	2238603	2817032
521	3,4'-Dihydroxyflavone	$C_9H_{11}NO_3$	$[M+H]^+$	Flavonoids	9	9192	13697	27083	34503
522	Chrysoeriol-8-C-glucoside-7-O-(6''-feruloyl)glucoside	$C_{11}H_{14}N_2O_3$	$[M+H]^+$	Flavonoids	178183	161338	129276	138810	184903
523	6,7,8-Tetrahydroxy-5-methoxyflavone*	$C_9H_{11}NO_3$	$[M+H]^+$	Flavonoids	43219	89403	73624	64376	89159
524	Vitexin-2''-O-rhamnoside	$C_{11}H_{14}N_2O_3$	$[M+H]^+$	Flavonoids	593951	889292	739156	648210	581505
525	Diosmetin-7-O-glucoside*	$C_9H_{11}NO_3$	$[M+H]^+$	Flavonoids	928395	1111640	801078	856861	820568
526	Violanthin	$C_{11}H_{14}N_2O_3$	$[M+H]^+$	Flavonoids	6493811	9207636	7246576	6669843	6069286

527	Chrysoeriol-8-C-glucoside (Scoparin)	$C_9H_{11}NO_3$	$[M+H]^+$	Flavonoids	205627	281399	202478	191376	174133
528	5,6,7-Tetrahydroxy-8-methoxy flavone*	$C_{11}H_{14}N_2O_3$	$[M+H]^+$	Flavonoids	25462	38490	27338	25528	13996
529	Tangeretin (4',5,6,7,8-Pentamethoxyflavon e)	$C_9H_{11}NO_3$	$[M+H]^+$	Flavonoids	267176	316295	206709	279325	958161
530	Ayanin (3',5-Dihydroxy-3,4',7-Trimeth oxyflavone)	$C_{11}H_{14}N_2O_3$	$[M+H]^+$	Flavonoids	41003	31733	34200	33067	61009
531	3,5,7,3'4'-Pentamethoxyflavon e	$C_9H_{11}NO_3$	$[M+H]^+$	Flavonoids	3081	14559	11125	18117	52071
532	5-Hydroxy-6,7,8,3',4'-pentamet hoxyflavone	$C_{11}H_{14}N_2O_3$	$[M+H]^+$	Flavonoids	14402	11833	10706	12186	14503
533	5,6,7,4'-Tetramethoxyflavone	$C_9H_{11}NO_3$	$[M+H]^+$	Flavonoids	9193	26093	21566	29863	67383
534	Tricin-7-O-Glucoside	$C_{11}H_{14}N_2O_3$	$[M+H]^+$	Flavonoids	736611	841645	628726	691776	811795
535	Apigenin-6-C-(2''-xylosyl)gluc oside*	$C_9H_{11}NO_3$	$[M+H]^+$	Flavonoids	2303975	1357836	1139426	1428587	1484779
536	Apigenin-8-C-(2''-xylosyl)gluc oside*	$C_{11}H_{14}N_2O_3$	$[M+H]^+$	Flavonoids	2303975	1357836	1139426	1428587	1484779
537	Apigenin-6,8-di-C-glucoside (Vicenin-2)	$C_9H_{11}NO_3$	$[M+H]^+$	Flavonoids	23053409	21411518	18998534	18856316	16278507
538	5,7,3',5'-tetrahydroxy-6-methyl favanone	$C_{11}H_{14}N_2O_3$	$[M+H]^+$	Flavonoids	47233	82252	64507	60982	82300
539	Apigenin-6,8-di-C-arabinoside *	$C_9H_{11}NO_3$	$[M+H]^+$	Flavonoids	103955	70807	42146	91378	143480
540	Diosmetin (5,7,3'-Trihydroxy-4'-methoxyf	$C_{11}H_{14}N_2O_3$	$[M+H]^+$	Flavonoids	47974	75219	65382	51763	77568

	lavone)*								
541	Nobiletin (5,6,7,8,3',4'-Hexamethoxyflavone)	$C_9H_{11}NO_3$	$[M+H]^+$	Flavonoids	129247	295778	200009	280324	781473
542	Chrysoeriol-7-O-rutinoside	$C_{11}H_{14}N_2O_3$	[M-H]-	Flavonoids	3016881	2473923	2252607	2535229	2471669
543	Apigenin-6-C-(2''-glucuronyl)xyloside	$C_9H_{11}NO_3$	$[M+H]^+$	Flavonoids	206947	286121	226330	194645	206397
544	Apigenin-6-C-(2''-glucosyl)arabinoside	$C_{11}H_{14}N_2O_3$	$[M+H]^+$	Flavonoids	33320479	20970576	16235395	26121424	22763875
545	5-Hydroxy-3,7,4'-trimethoxyflavone	$C_9H_{11}NO_3$	$[M+H]^+$	Flavonoids	42724	43147	36556	10899	50582
546	Luteolin-6-C-(2''-glucuronyl)glucoside	$C_{11}H_{14}N_2O_3$	$[M+H]^+$	Flavonoids	446075	565397	398174	345489	324782
547	4',5,7-Trihydroxy-3',6-dimethoxyflavone (Jaceosidin)	$C_9H_{11}NO_3$	$[M+H]^+$	Flavonoids	137308	120103	123929	144919	99548
548	Apiin	$C_{11}H_{14}N_2O_3$	$[M+H]^+$	Flavonoids	108178	57174	9	70779	199599
549	Vitexin-2''-O-xyloside	$C_9H_{11}NO_3$	$[M+H]^+$	Flavonoids	979203	667835	506857	891075	700556
550	Isovitexin-2''-O-xyloside*	$C_{11}H_{14}N_2O_3$	$[M+H]^+$	Flavonoids	1262293	814066	663589	1096427	856804
551	4',5-Dihydroxy-3,3',7-Trimethoxyflavone; Pachypodol	$C_9H_{11}NO_3$	$[M+H]^+$	Flavonoids	46682	34413	36278	37917	59818
552	Isovitexin-7-O-glucoside(Saponarin)	$C_{11}H_{14}N_2O_3$	[M-H]-	Flavonoids	449476	428766	377290	407265	362999
553	Apigenin-4'-O-glucoside*	$C_9H_{11}NO_3$	$[M+H]^+$	Flavonoids	122592	106504	87899	95925	93698
554	Vitexin-7-O-(6''-feruloyl)glucoside	$C_{11}H_{14}N_2O_3$	$[M+H]^+$	Flavonoids	395234	250126	231423	280050	278803
555	Chrysoeriol-5-O-glucoside	$C_9H_{11}NO_3$	[M-H]-	Flavonoids	304408	234063	378872	512327	311824
556	Vitexin-7-O-(6''-p-coumaroyl)glucoside	$C_{11}H_{14}N_2O_3$	$[M+H]^+$	Flavonoids	41127	34790	26320	23482	29526

557	Luteolin-7-O-glucoside (Cynaroside)*	$C_9H_{11}NO_3$	$[M+H]^+$	Flavonoids	282815	214420	136962	234283	275805
558	Chrysoeriol-5,7-di-O-glucoside	$C_{11}H_{14}N_2O_3$	$[M+H]^+$	Flavonoids	26516	26234	22825	14513	17585
559	Luteolin (5,7,3',4'-Tetrahydroxyflavone)	$C_9H_{11}NO_3$	[M-H]-	Flavonoids	10391	19086	16066	16411	15746
560	Tricin-7-O-(6''-O-malonyl)glucoside	$C_{11}H_{14}N_2O_3$	$[M+H]^+$	Flavonoids	68591	73883	42126	42610	43014
561	Acacetin-7-O-rutinoside (Linarin)	$C_9H_{11}NO_3$	$[M+H]^+$	Flavonoids	6392	8472	9361	49192	6848
562	Isoschaftoside	$C_{11}H_{14}N_2O_3$	$[M+H]^+$	Flavonoids	4789189	3186333	2546585	4430727	3679074
563	Apigenin-8-C-(2''-glucosyl)arabinoside	$C_9H_{11}NO_3$	$[M+H]^+$	Flavonoids	8815607	5294750	4532083	6842746	5868450
564	Luteolin-8-C-arabinoside	$C_{11}H_{14}N_2O_3$	$[M+H]^+$	Flavonoids	97911	139175	170799	131414	139132
565	Luteolin-6,8-di-C-glucoside*	$C_9H_{11}NO_3$	$[M+H]^+$	Flavonoids	151943	150237	125815	117957	124924
566	Apigenin-7,4'-dimethyl ether	$C_{11}H_{14}N_2O_3$	$[M+H]^+$	Flavonoids	6516	10055	5796	4438	13820
567	Luteolin-3'-O-glucoside*	$C_9H_{11}NO_3$	$[M+H]^+$	Flavonoids	113956	271432	281157	220154	134084
568	Apigenin-8-C-Glucoside (Vitexin)*	$C_{11}H_{14}N_2O_3$	[M-H]-	Flavonoids	2246139	2206853	2330964	2629430	2312256
569	Luteolin-6-C-glucoside (Isoorientin)*	$C_9H_{11}NO_3$	$[M+H]^+$	Flavonoids	31218	50739	50346	31574	41383
570	Luteolin-7-O-rutinoside*	$C_{11}H_{14}N_2O_3$	$[M+H]^+$	Flavonoids	2805263	1344928	1110044	1858586	2146473
571	3',4',5',5,7-Pentamethoxyflavone	$C_9H_{11}NO_3$	$[M+H]^+$	Flavonoids	3725	13719	7022	14074	39333
572	Apigenin-7-O-glucoside(Cosmosiin)*	$C_{11}H_{14}N_2O_3$	$[M+H]^+$	Flavonoids	114268	106781	93722	95627	99536
573	Sinensetin (5,6,7,3',4'-pentamethoxyflavo	$C_9H_{11}NO_3$	$[M+H]^+$	Flavonoids	2828	17675	15431	21421	58701

	ne)								
574	Hispidulin-7-O-(6''-O-p-Coumaroyl)Glucoside	$C_{11}H_{14}N_2O_3$	$[M+H]^+$	Flavonoids	9180	5326	6177	24054	9412
575	5,7,8-Tetrahydroxy-6-methoxyflavone*	$C_9H_{11}NO_3$	$[M+H]^+$	Flavonoids	16170	32180	26131	21033	13903
576	Apigenin-6-C-glucoside (Isovitexin)*	$C_{11}H_{14}N_2O_3$	[M-H]-	Flavonoids	2018751	1823001	1956014	2263337	1987911
577	Schaftoside	$C_9H_{11}NO_3$	$[M+H]^+$	Flavonoids	63873	35941	30020	39918	27764
578	Isovitexin-2''-O-(6'''-p-coumaroyl)glucoside	$C_{11}H_{14}N_2O_3$	$[M+H]^+$	Flavonoids	24864	8564	7259	17478	13927
579	5,6,7,3',4',5'-hexanmethoxyflavone	$C_9H_{11}NO_3$	[M-H]-	Flavonoids	62519	369428	263362	231354	197975
580	Apigenin; 4',5,7-Trihydroxyflavone	$C_{11}H_{14}N_2O_3$	$[M+H]^+$	Flavonoids	22252	46481	44039	52017	40123
581	Vitexin-7-O-rutinoside	$C_9H_{11}NO_3$	[M-H]-	Flavonoids	73749	35233	26719	55548	58227
582	Acacetin-7-O-glucuronide	$C_{11}H_{14}N_2O_3$	$[M+H]^+$	Flavonoids	4315	8668	7729	9453	8723
583	Chrysoeriol-7-O-(6''-malonyl)glucoside	$C_9H_{11}NO_3$	$[M+H]^+$	Flavonoids	50916	36693	38570	27269	22042
584	Apigenin-6-C-arabinoside-8-C-xyloside*	$C_{11}H_{14}N_2O_3$	$[M+H]^+$	Flavonoids	97990	77764	41456	85726	120562
585	Apigenin-7-O-neohesperidoside (Rhoifolin)	$C_9H_{11}NO_3$	$[M+H]^+$	Flavonoids	17290	10828	7073	13488	9088
586	Luteolin-8-C-glucoside (Orientin)*	$C_{11}H_{14}N_2O_3$	$[M+H]^+$	Flavonoids	28915	51365	45702	47334	45386
587	5,7-Dihydroxy-3',4',5'-trimethoxyflavone	$C_9H_{11}NO_3$	$[M+H]^+$	Flavonoids	7978	21296	19959	17418	12735
588	Luteolin-4'-O-glucoside*	$C_{11}H_{14}N_2O_3$	$[M+H]^+$	Flavonoids	114436	67725	94148	136267	83530
589	3'-O-Methyltricetin-7-O-glucos	$C_9H_{11}NO_3$	$[M+H]^+$	Flavonoids	16031	52339	62388	30720	36242

	ide								
590	Eupatilin-7-O-glucoside	$C_{11}H_{14}N_2O_3$	$[M+H]^+$	Flavonoids	16846	17396	18252	20620	13320
591	Orientin-6-C-arabinoside	$C_9H_{11}NO_3$	$[M+H]^+$	Flavonoids	55020	12476	20815	29678	41407
592	Luteolin-7-O-(6''-malonyl)glucoside*	$C_{11}H_{14}N_2O_3$	$[M+H]^+$	Flavonoids	24308	15223	8233	15634	30616
593	Isorhamnetin-3-O-neohesperidoside*	$C_9H_{11}NO_3$	$[M+H]^+$	Flavonoids	1948977	1066405	739482	1273567	1441009
594	Quercetin-3-O-neohesperidoside*	$C_{11}H_{14}N_2O_3$	$[M+H]^+$	Flavonoids	1196087	770167	812964	1042536	1024003
595	Isorhamnetin-3-O-sophoroside	$C_9H_{11}NO_3$	$[M+H]^+$	Flavonoids	20595	67714	68977	61275	65853
596	Quercetin-3-O-sophoroside-7-O-rhamnoside	$C_{11}H_{14}N_2O_3$	$[M+H]^+$	Flavonoids	124918	124315	113205	149530	154242
597	Kaempferol-3-O-glucoside-7-O-rhamnoside*	$C_9H_{11}NO_3$	$[M+H]^+$	Flavonoids	3488998	1989139	1643796	2453512	2797512
598	Quercetin-3-O-glucoside-7-O-rhamnoside*	$C_{11}H_{14}N_2O_3$	$[M+H]^+$	Flavonoids	1177681	693923	791493	1009686	980401
599	Quercetin-3-O-sambubioside	$C_9H_{11}NO_3$	$[M+H]^+$	Flavonoids	35562	41680	28604	36297	33749
600	Isorhamnetin-3-O-rutinoside-4'-O-glucoside*	$C_{11}H_{14}N_2O_3$	$[M+H]^+$	Flavonoids	13210	33012	26697	38532	29436
601	Kaempferol-3-O-(2''-O-acetyl)glucuronide	$C_9H_{11}NO_3$	[M-H]-	Flavonoids	49563	114387	74551	110195	80832
602	Quercetin-7-O-(6''-malonyl)glucoside	$C_{11}H_{14}N_2O_3$	$[M+H]^+$	Flavonoids	123730	65221	65352	96273	103383
603	Kaempferol-6,8-di-C-glucoside*	$C_9H_{11}NO_3$	$[M+H]^+$	Flavonoids	154506	159020	138035	131446	120826
604	Quercetin-3-O-xyloside (Reynoutrin)*	$C_{11}H_{14}N_2O_3$	$[M+H]^+$	Flavonoids	353030	187925	269343	277913	293049
605	Kaempferol-3-O-neohesperido	$C_9H_{11}NO_3$	$[M+H]^+$	Flavonoids	3789776	1869923	1647296	2593936	2704893

	side*								
606	Isorhamnetin-3-O-Glucoside*	$C_{11}H_{14}N_2O_3$	$[M+H]^+$	Flavonoids	225804	191924	239533	278482	259069
607	Quercetin-3-O-rutinoside (Rutin)*	$C_9H_{11}NO_3$	$[M+H]^+$	Flavonoids	1172569	679240	808257	995554	989299
608	Quercetin-7-O-rutinoside*	$C_{11}H_{14}N_2O_3$	$[M+H]^+$	Flavonoids	1301184	725903	851572	1075808	1003833
609	Quercetin-3-O-glucoside (Isoquercitrin)*	$C_9H_{11}NO_3$	$[M+H]^+$	Flavonoids	3629768	1994033	3132896	3775017	2992534
610	Isorhamnetin-7-O-glucoside (Brassicin)*	$C_{11}H_{14}N_2O_3$	$[M+H]^+$	Flavonoids	249257	192790	229726	302107	261046
611	Isorhamnetin-3-O-rutinoside (Narcissin)*	$C_9H_{11}NO_3$	$[M+H]^+$	Flavonoids	1987851	1119427	815013	1212017	1507362
612	Kaempferol-3-O-rutinoside(Nicotiflorin)*	$C_{11}H_{14}N_2O_3$	$[M+H]^+$	Flavonoids	3292872	1668696	1511575	2336365	2879817
613	Quercetin-3-O-(6''-O-p-coumaroyl)sophoroside-7-O-rhamnoside	$C_9H_{11}NO_3$	$[M+H]^+$	Flavonoids	35462	132044	115435	75448	98119
614	Isorhamnetin; 3'-Methoxy-3,4',5,7-Tetrahydroxyflavone	$C_{11}H_{14}N_2O_3$	[M-H]-	Flavonoids	20524	15838	59594	39094	22104
615	Kaempferol-3-O-glucoside (Astragalin)*	$C_9H_{11}NO_3$	$[M+H]^+$	Flavonoids	118852	73203	112614	139736	87665
616	Isorhamnetin-3-O-(6''-acetylglucoside)	$C_{11}H_{14}N_2O_3$	[M-H]-	Flavonoids	18602	11852	6567	9148	14045
617	Quercetin	$C_9H_{11}NO_3$	$[M+H]^+$	Flavonoids	18299	7264	94467	46917	23283
618	Quercetin-4'-O-glucoside (Spiraeoside)*	$C_{11}H_{14}N_2O_3$	[M-H]-	Flavonoids	1780852	1207352	1926483	2455895	1943571
619	Quercetin-5-O-β-D-glucoside*	$C_9H_{11}NO_3$	$[M+H]^+$	Flavonoids	3368466	2081534	3094579	4023575	3116614
620	Kaempferol-3-O-glucorhamno	$C_{11}H_{14}N_2O_3$	$[M+H]^+$	Flavonoids	3500625	1946599	1506333	2518992	2574354

	side*								
621	Quercetin-3-O-rutinoside-7-O-glucoside	$C_9H_{11}NO_3$	$[M+H]^+$	Flavonoids	113230	107438	109810	135007	132776
622	Quercetin-3-O-(4''-O-glucosyl) rhamnoside*	$C_{11}H_{14}N_2O_3$	$[M+H]^+$	Flavonoids	1300928	791283	874306	1198855	1093412
623	Avicularin(Quercetin-3-O-α-L-arabinofuranoside)	$C_9H_{11}NO_3$	$[M+H]^+$	Flavonoids	342027	246225	267967	307105	280060
624	Quercetin-3-O-arabinoside*	$C_{11}H_{14}N_2O_3$	[M-H]-	Flavonoids	204668	144019	212056	229632	267360
625	3,5,6,7,8,3',4'-Heptamethoxyflavone	$C_9H_{11}NO_3$	$[M+H]^+$	Flavonoids	91783	21548	10609	26111	57840
626	Kaempferol-4'-O-glucoside*	$C_{11}H_{14}N_2O_3$	$[M+H]^+$	Flavonoids	113360	79153	87876	155494	101724
627	Kaempferol-7-O-glucoside*	$C_9H_{11}NO_3$	[M-H]-	Flavonoids	28207	29489	29546	41752	32036
628	Kaempferol-3-O-sophoroside	$C_{11}H_{14}N_2O_3$	[M-H]-	Flavonoids	10810	7176	15844	16853	10213
629	Quercetin-3-O-galactoside (Hyperin)*	$C_9H_{11}NO_3$	$[M+H]^+$	Flavonoids	3676112	2118795	3215191	4042526	2907756
630	Quercetin-3-O-(2''-O-rhamnosyl)galactoside	$C_{11}H_{14}N_2O_3$	$[M+H]^+$	Flavonoids	47261	149346	97426	64865	85247
631	Limocitrin-3-O-galactoside	$C_9H_{11}NO_3$	$[M+H]^+$	Flavonoids	58524	21318	27461	31431	45225
632	Kaempferol-3-O-sophoroside-7-O-rhamnoside*	$C_{11}H_{14}N_2O_3$	$[M+H]^+$	Flavonoids	5603	10053	9219	11921	12085
633	Quercetin-7-O-(2''-malonyl)glucosyl-5-O-glucoside	$C_9H_{11}NO_3$	$[M+H]^+$	Flavonoids	48182	31640	13820	24336	12313
634	Quercetin-3-O-glucosyl(1→4)rhamnoside-7-O-rutinoside	$C_{11}H_{14}N_2O_3$	$[M+H]^+$	Flavonoids	49258	153453	113187	79341	105970
635	Quercetagetin-4'-Methyl Ether	$C_9H_{11}NO_3$	[M-H]-	Flavonoids	5312	16065	7769	13530	8133
636	Isorhamnetin-3,7-O-diglucoside	$C_{11}H_{14}N_2O_3$	$[M+H]^+$	Flavonoids	13044	35702	33880	33968	28606
637	Kaempferol	$C_9H_{11}NO_3$	[M-H]-	Flavonoids	5965	9	17366	12634	9804

	(3,5,7,4'-Tetrahydroxyflavone)								
638	3,5,4'-Trihydroxy-7-methoxyflavone (Rhamnocitrin)*	$C_{11}H_{14}N_2O_3$	$[M+H]^+$	Flavonoids	42977	69929	55298	45341	62768
639	Quercetin-3-O-rhamnoside(Quercitrin)	$C_9H_{11}NO_3$	$[M+H]^+$	Flavonoids	46727	26392	33130	39493	50446
640	Quercetin-3-O-sophoroside (Baimaside)	$C_{11}H_{14}N_2O_3$	$[M+H]^+$	Flavonoids	109377	119209	120943	119340	77089
641	Kaempferol-3-O-neohesperidoside-7-O-glucoside*	$C_9H_{11}NO_3$	$[M+H]^+$	Flavonoids	5398	14738	7376	13012	11364
642	Kaempferol-3-O-(6''-malonyl) glucoside*	$C_{11}H_{14}N_2O_3$	$[M+H]^+$	Flavonoids	19970	14887	10487	8108	27929
643	Quercetin-3,7-Di-O-glucoside	$C_9H_{11}NO_3$	$[M+H]^+$	Flavonoids	9	23110	23086	29833	22584
644	Isorhamnetin-3-O-sophoroside-7-O-rhamnoside*	$C_{11}H_{14}N_2O_3$	$[M+H]^+$	Flavonoids	8009	35450	30058	33814	31600
645	Quercetin-7-O-glucoside*	$C_9H_{11}NO_3$	[M-H]-	Flavonoids	123146	209149	178881	186068	204704
646	Kaempferol-3,7-O-dirhamnoside (Kaempferitrin)	$C_{11}H_{14}N_2O_3$	$[M+H]^+$	Flavonoids	29995	39322	29410	20451	32469
647	Quercetin-3-O-(6''-O-acetyl)galactoside	$C_9H_{11}NO_3$	[M-H]-	Flavonoids	42980	19631	22574	35205	54396
648	Quercetin-3,3'-dimethyl ether	$C_{11}H_{14}N_2O_3$	[M-H]-	Flavonoids	7687	5858	9	3719	3100
649	Kaempferol-3-O-galactoside (Trifolin)*	$C_9H_{11}NO_3$	[M-H]-	Flavonoids	35361	22320	40424	37552	25363
650	Quercetin-3-O-(2'''-O-p-coumaroyl)sophoroside-7-O-glucoside	$C_{11}H_{14}N_2O_3$	$[M+H]^+$	Flavonoids	76182	31075	48528	57354	57444
651	Isorhamnetin-3-O-(6''-malonyl)glucoside	$C_9H_{11}NO_3$	$[M+H]^+$	Flavonoids	65534	31320	18372	30022	46074
652	Quercetin-3-O-rutinoside-7-O-	$C_{11}H_{14}N_2O_3$	$[M+H]^+$	Flavonoids	149287	71290	79306	100955	108114

	rhamnoside								
653	Kaempferol-3-O-glucuronide	$C_9H_{11}NO_3$	[M-H]-	Flavonoids	8471	9884	12237	12845	17257
654	Epicatechin gallate*	$C_{11}H_{14}N_2O_3$	[M-H]-	Flavonoids	6465	7886	14126	14674	31233
655	Catechin gallate*	$C_9H_{11}NO_3$	[M-H]-	Flavonoids	35694	39496	42535	46270	41326
656	5-Hydroxyquinoline	$C_{11}H_{14}N_2O_3$	$[M+H]^+$	Alkaloids	37634	34939	38519	24471	23702
657	2,4-Dihydroxyquinoline	$C_9H_{11}NO_3$	$[M+H]^+$	Alkaloids	9	27436	19179	19552	19989
658	4-Hydroxyquinoline	$C_{11}H_{14}N_2O_3$	[M-H]-	Alkaloids	15668	24027	22285	39209	32121
659	2,6-Dimethoxy-1,4-benzoquinone*	$C_9H_{11}NO_3$	$[M+H]^+$	Quinones	1473983	1695074	1453206	1313651	1041412
660	2,5-Dimethoxybenzoquinone*	$C_{11}H_{14}N_2O_3$	$[M+H]^+$	Quinones	137510	135029	123470	128876	92413
661	Choline Alfoscerate	$C_9H_{11}NO_3$	$[M+H]^+$	Lipids	119275	8055555	8375327	8534672	1441260
662	Medioresinol-4,4'-di-O-glucoside	$C_{11}H_{14}N_2O_3$	[M-H]-	Lignans and Coumarins	214516	1177731	1195294	982442	680596
663	Syringaresinol-4'-O-(6''-acetyl) glucoside	$C_9H_{11}NO_3$	[M-H]-	Lignans and Coumarins	4793327	6237978	7405098	5070065	3358198
664	4-Ketopinoresinol	$C_{11}H_{14}N_2O_3$	[M-H]-	Lignans and Coumarins	10340	35360	41866	45718	68094
665	Matairesinol-4'-O-glucoside (Matairesinoside)	$C_9H_{11}NO_3$	$[M+H]^+$	Lignans and Coumarins	1684533	1945057	2093226	1384214	1436743
666	Syringaresinol-4'-O-glucoside	$C_{11}H_{14}N_2O_3$	[M-H]-	Lignans and Coumarins	17115845	21682539	28178138	23456683	24411083
667	5'-Methoxyisolariciresinol-9'-O-glucoside	$C_9H_{11}NO_3$	[M-H]-	Lignans and Coumarins	1302106	953002	1771736	1353884	1299682
668	Dehydrodiconiferyl alcohol	$C_{11}H_{14}N_2O_3$	$[M+H]^+$	Lignans and Coumarins	118972	71317	78142	88423	51339
669	Matairesinol	$C_9H_{11}NO_3$	$[M+H]^+$	Lignans and Coumarins	294892	155035	174856	160010	127019
670	Dihydrodehydrodiconiferyl	$C_{11}H_{14}N_2O_3$	[M-H]-	Lignans and	728573	511788	795157	603369	536266

	alcohol-4-O-glucoside*			Coumarins					
671	Epipinoresinol*	$C_9H_{11}NO_3$	[M-H]-	Lignans and Coumarins	74499	104916	98039	95837	109188
672	Secoisolariciresinol 4-O-glucoside	$C_{11}H_{14}N_2O_3$	[M-H]-	Lignans and Coumarins	2489083	1976636	3207159	2312371	2727315
673	Pinoresinol*	$C_9H_{11}NO_3$	[M-H]-	Lignans and Coumarins	73333	105363	110269	97472	103462
674	Pinoresinol-4-O-glucoside	$C_{11}H_{14}N_2O_3$	[M-H]-	Lignans and Coumarins	97889	171622	160780	128074	168490
675	5'-Methoxymatairesinoside	$C_9H_{11}NO_3$	[M-H]-	Lignans and Coumarins	273688	353116	289487	411836	311243
676	Olivil-4'-O-glucoside	$C_{11}H_{14}N_2O_3$	[M-H]-	Lignans and Coumarins	257522	283274	315618	245981	177155
677	Isolariciresinol-9'-O-glucoside *	$C_9H_{11}NO_3$	[M-H]-	Lignans and Coumarins	780690	551756	783724	726597	721633
678	Olivil-4,4'-Di-O-glucoside	$C_{11}H_{14}N_2O_3$	[M-H]-	Lignans and Coumarins	129352	197484	231027	128508	118806
679	1-Hydroxypinoresinol-1-O-Glucoside	$C_9H_{11}NO_3$	[M-H]-	Lignans and Coumarins	156455	86840	90417	92664	99573
680	Isolariciresinol	$C_{11}H_{14}N_2O_3$	[M-H]-	Lignans and Coumarins	9365	6254	8402	6365	3821
681	Syringaresinol	$C_9H_{11}NO_3$	[M-H]-	Lignans and Coumarins	1032664	1125280	1321384	964089	931136
682	Lirioresinol A	$C_{11}H_{14}N_2O_3$	[M-H]-	Lignans and Coumarins	4335607	4607035	5688604	4090150	4061853
683	3,4-Methylenedioxy cinnamyl alcohol	$C_9H_{11}NO_3$	[M-H]-	Lignans and Coumarins	91864	106012	89184	105618	57026
684	Pinoresinol-4,4'-O-di-O-glucos	$C_{11}H_{14}N_2O_3$	[M-H]-	Lignans and	242841	769729	829657	694473	653283

	ide			Coumarins					
685	Medioresinol-4'-O-(6'''-acetyl) glucoside	$C_9H_{11}NO_3$	[M-H]-	Lignans and Coumarins	82050	112669	103442	76093	59570
686	Lyoniresinol	$C_{11}H_{14}N_2O_3$	[M-H]-	Lignans and Coumarins	23799	26907	44940	50237	50383
687	Pinoresinol-4,4'-O-β-D-bisglucopyranoside	$C_9H_{11}NO_3$	$[M+H]^+$	Lignans and Coumarins	14112	17417	14498	19777	8886
688	8-Hydroxy-α-conidendrin	$C_{11}H_{14}N_2O_3$	[M-H]-	Lignans and Coumarins	9608	13959	8433	17212	15809
689	Senkyunolide K	$C_9H_{11}NO_3$	$[M+H]^+$	Others	310306	3058	5506	5440	9
690	Senkyunolide M	$C_{11}H_{14}N_2O_3$	$[M+H]^+$	Others	3221204	3596126	3543731	3396420	3740775
691	Senkyunolide B	$C_9H_{11}NO_3$	$[M+H]^+$	Others	1113091	1341197	1512942	1365916	1490039
692	Hydroxydihydrobovolide	$C_{11}H_{14}N_2O_3$	[M-H]-	Others	158435	1039949	1298170	1254630	1233255
693	2-Piperidone	$C_9H_{11}NO_3$	$[M+H]^+$	Alkaloids	250507	201390	296743	502758	114226
694	Piperidine	$C_{11}H_{14}N_2O_3$	$[M+H]^+$	Alkaloids	6060462	3760998	4092326	5793546	2600560
695	N-Hydroxypipecolic acid	$C_9H_{11}NO_3$	$[M+H]^+$	Alkaloids	106997	207715	107630	130967	138696
696	6-Deoxyfagomine	$C_{11}H_{14}N_2O_3$	$[M+H]^+$	Alkaloids	8030751	5354835	5472800	7637945	3655819
697	2-Ethyl-2,6,6-trimethylpiperidin-4-one	$C_9H_{11}NO_3$	$[M+H]^+$	Alkaloids	135835	42844	38649	41003	71976
698	2,6-Dimethoxypydroquinone-1-O-glucoside	$C_{11}H_{14}N_2O_3$	$[M+H]^+$	Flavonoids	17658	239617	1030643	163354	619964
699	7-Methoxy-3-[1-(3-pyridyl)methylidene]-4-chromanone	$C_9H_{11}NO_3$	$[M+H]^+$	Flavonoids	2542872	6130873	5613399	6847851	5802467
700	1,3,6,8-Tetrahydroxy-2-methoxyxanthone	$C_{11}H_{14}N_2O_3$	[M-H]-	Flavonoids	15056	30928	24210	21182	20949
701	5-hydroxy-3-(2-hydroxy-4-methoxybenzyl)-7-methoxychroman-4-one	$C_9H_{11}NO_3$	$[M+H]^+$	Flavonoids	412284	1212725	890721	503604	722487

702	6,7-dihydroxy-1,3-dimethoxyxanthen-9-one	$C_{11}H_{14}N_2O_3$	[M-H]-	Flavonoids	275497	641196	579454	524100	469472
703	5-hydroxy-2-methoxyxanthen-9-one	$C_9H_{11}NO_3$	[M-H]-	Flavonoids	26618	40205	38123	37035	33687
704	1,8-dihydroxy-2,6-dimethylxanthen-9-one	$C_{11}H_{14}N_2O_3$	[M-H]-	Flavonoids	9	120380	138794	115834	154880
705	1-Glycosyloxy-2-hydroxy-4-methoxyxanthone	$C_9H_{11}NO_3$	$[M+H]^+$	Flavonoids	11650	10022	27666	13957	12114
706	Hexadecylsphingosine	$C_{11}H_{14}N_2O_3$	$[M+H]^+$	Lipids	2053506	2073292	1899120	1937796	2336614
707	3-Dehydrosphinganine	$C_9H_{11}NO_3$	$[M+H]^+$	Lipids	12571	10504	9201	12409	7028
708	4-Hydroxysphinganine	$C_{11}H_{14}N_2O_3$	$[M+H]^+$	Lipids	6278187	6131991	6393737	6765104	4394210
709	D-Sphingosine	$C_9H_{11}NO_3$	$[M+H]^+$	Lipids	29147	18268	18818	20271	11490
710	4-Guanidinobutanal	$C_{11}H_{14}N_2O_3$	$[M+H]^+$	Others	2509691	2775866	4684272	6224465	4317461
711	5-Hydroxymethylfurfural	$C_9H_{11}NO_3$	$[M+H]^+$	Others	9	609702	458509	480744	8578004
712	4-hydroxyphenyl acrylaldehyde	$C_{11}H_{14}N_2O_3$	$[M+H]^+$	Others	112895	208673	213154	176179	225508
713	4-Methylbenzaldehyde*	$C_9H_{11}NO_3$	[M-H]-	Others	89577	205824	258904	208189	315597
714	3-Methylbenzaldehyde*	$C_{11}H_{14}N_2O_3$	[M-H]-	Others	89577	205824	258904	208189	315597
715	2,4-Dihydroxybenzaldehyde	$C_9H_{11}NO_3$	[M-H]-	Others	16781	10932	17848	23865	17001
716	LysoPC 18:1(2n isomer)	$C_{11}H_{14}N_2O_3$	$[M+H]^+$	Lipids	371210	16255864	62769267	65140689	11650939
717	LysoPC 15:0	$C_9H_{11}NO_3$	$[M+H]^+$	Lipids	28508	5408207	16074329	16985838	4557463
718	LysoPC 18:2	$C_{11}H_{14}N_2O_3$	$[M+H]^+$	Lipids	7393	1010062	3094351	2973050	725434
719	LysoPC 19:0	$C_9H_{11}NO_3$	$[M+H]^+$	Lipids	9	71417	382468	935756	9
720	LysoPC 20:2	$C_{11}H_{14}N_2O_3$	$[M+H]^+$	Lipids	3636	940110	505372	637449	722750
721	LysoPC 16:1	$C_9H_{11}NO_3$	$[M+H]^+$	Lipids	13692	191007	455649	472686	259592
722	LysoPC 18:3	$C_{11}H_{14}N_2O_3$	$[M+H]^+$	Lipids	129688	13057240	38569072	32967621	17430833
723	LysoPC 17:2	$C_9H_{11}NO_3$	$[M+H]^+$	Lipids	5553	339572	1674651	1688105	269147
724	LysoPC 16:1(2n isomer)	$C_{11}H_{14}N_2O_3$	$[M+H]^+$	Lipids	20329	805419	3744970	4494340	560538

725	LysoPC 16:0	$C_9H_{11}NO_3$	$[M+H]^+$	Lipids	352663	56331655	28902349	39846809	39763410
726	LysoPC 17:0(2n isomer)	$C_{11}H_{14}N_2O_3$	$[M+H]^+$	Lipids	6969	748723	865304	1234033	919146
727	LysoPC 15:0(2n isomer)	$C_9H_{11}NO_3$	$[M+H]^+$	Lipids	34257	7141661	20774884	21077548	5938148
728	LysoPC 18:3(2n isomer)	$C_{11}H_{14}N_2O_3$	$[M+H]^+$	Lipids	4421	396841	1191036	1053271	544073
729	LysoPC 18:0(2n isomer)	$C_9H_{11}NO_3$	$[M+H]^+$	Lipids	16079	1297056	1481115	2072877	1623320
730	LysoPC 16:0(2n isomer)	$C_{11}H_{14}N_2O_3$	$[M+H]^+$	Lipids	76425	6459434	9284298	11552858	6702791
731	LysoPC 14:0	$C_9H_{11}NO_3$	$[M+H]^+$	Lipids	10174	2287437	6601680	6897830	2402939
732	LysoPC 20:1	$C_{11}H_{14}N_2O_3$	$[M+H]^+$	Lipids	1577	230272	1084398	1140198	127386
733	LysoPC 12:0	$C_9H_{11}NO_3$	$[M+H]^+$	Lipids	9	112175	64405	86542	88450
734	LysoPC 18:0	$C_{11}H_{14}N_2O_3$	$[M+H]^+$	Lipids	74835	13146382	6185108	9533805	8380151
735	LysoPC 16:2	$C_9H_{11}NO_3$	$[M+H]^+$	Lipids	9	31687	59493	9	42013
736	LysoPC 17:0	$C_{11}H_{14}N_2O_3$	$[M+H]^+$	Lipids	30943	6385439	2917805	5059462	4164460
737	LysoPC 19:2(2n isomer)	$C_9H_{11}NO_3$	$[M+H]^+$	Lipids	10630	232107	794343	1292537	104522
738	LysoPC 18:4	$C_{11}H_{14}N_2O_3$	$[M+H]^+$	Lipids	9	8305	74887	137195	8886
739	LysoPC 19:1	$C_9H_{11}NO_3$	$[M+H]^+$	Lipids	9	102440	141325	142190	80969
740	LysoPC 20:3	$C_{11}H_{14}N_2O_3$	$[M+H]^+$	Lipids	9	131805	90494	113603	106617
741	LysoPC 16:2(2n isomer)	$C_9H_{11}NO_3$	$[M+H]^+$	Lipids	9	30703	52830	47543	42599
742	LysoPC 19:2	$C_{11}H_{14}N_2O_3$	$[M+H]^+$	Lipids	4397	107679	295151	542171	57773
743	LysoPC 18:1	$C_9H_{11}NO_3$	$[M+H]^+$	Lipids	115492	6726432	13681827	12362320	5347228
744	LysoPC 15:1	$C_{11}H_{14}N_2O_3$	$[M+H]^+$	Lipids	9	481490	297325	297915	268059
745	LysoPC 17:1	$C_9H_{11}NO_3$	$[M+H]^+$	Lipids	4847	195233	129717	273170	161024
746	LysoPE 14:0	$C_{11}H_{14}N_2O_3$	$[M+H]^+$	Lipids	30395	4572335	2284072	3082417	2328347
747	LysoPE 20:4	$C_9H_{11}NO_3$	$[M+H]^+$	Lipids	9	8446	19820	19807	4345
748	LysoPE 18:1	$C_{11}H_{14}N_2O_3$	$[M+H]^+$	Lipids	130884	1389658	2419361	2677298	866398
749	LysoPE 18:1(2n isomer)	$C_9H_{11}NO_3$	$[M+H]^+$	Lipids	704036	2338587	14613614	17580089	741484
750	LysoPE 17:0	$C_{11}H_{14}N_2O_3$	$[M+H]^+$	Lipids	3065	197464	186583	239206	136848
751	LysoPE 20:2	$C_9H_{11}NO_3$	$[M+H]^+$	Lipids	9	207661	141656	154350	67783
752	LysoPE 16:0	$C_{11}H_{14}N_2O_3$	$[M+H]^+$	Lipids	504406	19538277	10930405	15310762	8832228

753	LysoPE 18:2	$C_9H_{11}NO_3$	$[M+H]^+$	Lipids	9111	967342	3236944	3167537	345345
754	LysoPE 18:0	$C_{11}H_{14}N_2O_3$	$[M+H]^+$	Lipids	21315	925651	525602	771513	351671
755	LysoPE 15:1(2n isomer)	$C_9H_{11}NO_3$	$[M+H]^+$	Lipids	9	19208	28818	27599	8383
756	LysoPE 15:0(2n isomer)	$C_{11}H_{14}N_2O_3$	$[M+H]^+$	Lipids	13324	1440020	1917345	1990628	881679
757	LysoPE 18:3(2n isomer)	$C_9H_{11}NO_3$	$[M+H]^+$	Lipids	58646	7359867	31504465	30376950	2415776
758	LysoPE 15:0	$C_{11}H_{14}N_2O_3$	$[M+H]^+$	Lipids	71666	6772449	2040992	2806701	3470200
759	LysoPE 16:0(2n isomer)	$C_9H_{11}NO_3$	$[M+H]^+$	Lipids	66447	3179601	2825686	3732832	2066117
760	LysoPE 20:3(2n isomer)	$C_{11}H_{14}N_2O_3$	$[M+H]^+$	Lipids	9	23310	16947	20496	10682
761	LysoPE 17:1	$C_9H_{11}NO_3$	$[M+H]^+$	Lipids	5376	33999	537527	315398	52369
762	LysoPE 16:1(2n isomer)	$C_{11}H_{14}N_2O_3$	$[M+H]^+$	Lipids	19544	98512	1079431	887452	49426
763	LysoPE 15:1	$C_9H_{11}NO_3$	$[M+H]^+$	Lipids	9	121027	47688	60625	41131
764	LysoPE 18:3	$C_{11}H_{14}N_2O_3$	$[M+H]^+$	Lipids	104531	7952277	34040836	33112155	2409776
765	LysoPE 20:2(2n isomer)	$C_9H_{11}NO_3$	$[M+H]^+$	Lipids	9	108777	299015	289297	32833
766	LysoPE 20:4(2n isomer)	$C_{11}H_{14}N_2O_3$	$[M+H]^+$	Lipids	9	9795	21412	19142	5141
767	LysoPE 18:4	$C_9H_{11}NO_3$	$[M+H]^+$	Lipids	9	9	33807	38438	9
768	LysoPE 16:1	$C_{11}H_{14}N_2O_3$	$[M+H]^+$	Lipids	9998	50917	257313	172033	66509
769	LysoPE 20:3	$C_9H_{11}NO_3$	$[M+H]^+$	Lipids	9	23023	15887	20884	9705
770	3-Hydroxyurs-12-en-28-oic acid (Ursolic acid)	$C_{11}H_{14}N_2O_3$	[M-H]-	Terpenoids	73062	24218	22570	28326	31128
771	12,13-Dihydroursolic acid	$C_9H_{11}NO_3$	$[M+H]^+$	Terpenoids	21990	39089	37312	40989	39275
772	3-(Acetyloxy)-13,15-dihydroxyoleanan-12-one (Rubiprasin A)(iso-01)	$C_{11}H_{14}N_2O_3$	$[M+H]^+$	Terpenoids	17501	45640	20967	43010	31292
773	24,30-Dihydroxy-12(13)-ene-lupeol*	$C_9H_{11}NO_3$	[M-H]-	Terpenoids	337862	114660	213338	423100	85197
774	2,3,23-Trihydroxyolean-12-ene-28,29-dioic acid 29-methyl ester (Phytolaccagenin)	$C_{11}H_{14}N_2O_3$	[M-H]-	Terpenoids	52821	108698	77243	77803	76876

775	9,19-Cyclolanost-24-en-3-ol (Cycloartenol)	$C_9H_{11}NO_3$	$[M+H]^+$	Terpenoids	13796	11939	12566	12493	12689
776	2,19-Dihydroxy-3-oxours-12-en-28-oic acid	$C_{11}H_{14}N_2O_3$	[M-H]-	Terpenoids	110346	146919	301260	328282	180637
777	2,3,16,21-Tetrahydroxyolean-12-en-28-oic acid (Platycogenic acid C)	$C_9H_{11}NO_3$	[M-H]-	Terpenoids	239916	80970	997892	1149909	103823
778	3-Hydroxylup-20(29)-en-28-oic acid (Betulinic acid)	$C_{11}H_{14}N_2O_3$	$[M+H]^+$	Terpenoids	22841	22560	37267	39667	20902
779	3-Hydroxy-11-oxours-12-en-28-oic acid (11-Keto-ursolic acid)	$C_9H_{11}NO_3$	$[M+H]^+$	Terpenoids	34461	26067	30019	34781	25452
780	19-Hydroxy-2-(hydroxymethyl)-A(1)-norursa-2,12-dien-28-oic acid (Hyptadienic acid)	$C_{11}H_{14}N_2O_3$	[M-H]-	Terpenoids	22540	23084	21943	24882	33396
781	3-Hydroxyolean-12-en-28-oic acid (Oleanolic acid)*	$C_9H_{11}NO_3$	[M-H]-	Terpenoids	68951	49054	60279	71913	37601
782	2,3,6,23-Tetrahydroxyurs-12-en-28-oic acid (Madecassic acid)	$C_{11}H_{14}N_2O_3$	[M-H]-	Terpenoids	9951	16452	13976	13474	11356
783	Lup-20(29)-en-3-one (Lupenone)	$C_9H_{11}NO_3$	$[M+H]^+$	Terpenoids	42165	34929	44415	45771	29567
784	3,25-Epoxy-3-hydroxyolean-18-en-28-oic acid (Semimoronic acid)	$C_{11}H_{14}N_2O_3$	[M-H]-	Terpenoids	25640	162020	42512	69239	44070
785	Urs-12(13)-en-3-one-28-oic acid*	$C_9H_{11}NO_3$	[M-H]-	Terpenoids	14305	15077	47432	66213	7739
786	3-Oxolup-20(29)-en-28-oic	$C_{11}H_{14}N_2O_3$	[M-H]-	Terpenoids	21639	25200	44174	50802	14954

	acid (Betulonic acid)*								
787	Oleanolic acid-3-O-xylosyl(1→3)glucuronide	$C_9H_{11}NO_3$	[M-H]-	Terpenoids	9	9	61137	73778	9
788	6-Demethoxycapillarisin	$C_{11}H_{14}N_2O_3$	$[M+H]^+$	Others	131235	191752	106002	120051	58101
789	6,7-dimethoxy-2-[2-(4'-hydroxy-3'-methoxyphenyl)ethyl]chromone	$C_9H_{11}NO_3$	$[M+H]^+$	Others	612183	363300	308093	393575	252076
790	Capillarisin	$C_{11}H_{14}N_2O_3$	$[M+H]^+$	Others	18125	7367	43920	27729	21636
791	6,7-dimethoxy-2-[2-phenylethyl]chromone	$C_9H_{11}NO_3$	$[M+H]^+$	Others	29056	25077	26440	38439	45799
792	3,5,7,4'-Tetrahydroxy-Coumaronochromone	$C_{11}H_{14}N_2O_3$	$[M+H]^+$	Others	165778	95425	102106	86593	122551
793	L-Tyramine	$C_9H_{11}NO_3$	$[M+H]^+$	Alkaloids	2861643	4857068	6042149	5352855	1899300
794	N-Acetylcadaverine	$C_{11}H_{14}N_2O_3$	$[M+H]^+$	Alkaloids	722001	739980	698775	738911	690905
795	Histidinol	$C_9H_{11}NO_3$	$[M+H]^+$	Alkaloids	23745299	23656103	24234559	25603944	25308036
796	Agmatine	$C_{11}H_{14}N_2O_3$	$[M+H]^+$	Alkaloids	508623	302729	346962	463767	137711
797	Cadaverine	$C_9H_{11}NO_3$	$[M+H]^+$	Alkaloids	25025205	922425	2253149	1078738	267855
798	octadecadienoic acid amide	$C_{11}H_{14}N_2O_3$	$[M+H]^+$	Alkaloids	824144	1061680	2945901	1391631	592930
799	N-Acetylputrescine	$C_9H_{11}NO_3$	$[M+H]^+$	Alkaloids	490848	298513	330099	437654	116406
800	4,5,6-Trihydroxy-2-cyclohexen-1-ylideneacetonitrile	$C_{11}H_{14}N_2O_3$	$[M+H]^+$	Alkaloids	458003	191203	212686	299244	111703
801	Diethanolamine	$C_9H_{11}NO_3$	$[M+H]^+$	Alkaloids	218416	121385	121705	132457	72685
802	N-(4-oxopentyl)-acetamide	$C_{11}H_{14}N_2O_3$	$[M+H]^+$	Alkaloids	2062242	2484507	1820018	2017658	2496296
803	N-Benzylmethylene isomethylamine	$C_9H_{11}NO_3$	$[M+H]^+$	Alkaloids	8372393	3528322	3900367	5357435	1732641
804	N-benzylformamide	$C_{11}H_{14}N_2O_3$	$[M+H]^+$	Alkaloids	1286402	739081	947490	1180580	467692
805	N-(4-hydroxyphenethyl)cinna	$C_9H_{11}NO_3$	$[M+H]^+$	Alkaloids	1263822	1776788	1756357	1204566	1023415

	mamide								
806	N-benzoyl-2-aminoethyl-β-D-glucopyranoside	$C_{11}H_{14}N_2O_3$	$[M+H]^+$	Alkaloids	1286810	846867	1603392	2322999	6041195
807	N-(4-Aminobutyl)benzamide	$C_9H_{11}NO_3$	$[M+H]^+$	Alkaloids	127585	141344	251852	190813	183543
808	Choline	$C_{11}H_{14}N_2O_3$	[M]+	Alkaloids	28638902	24020326	14735449	18738154	11903582
809	Betaine	$C_9H_{11}NO_3$	$[M+H]^+$	Alkaloids	36785302	5098086	3583731	4372855	2589667
810	3-amino-2-naphthoic acid	$C_{11}H_{14}N_2O_3$	$[M+H]^+$	Alkaloids	7918329	4015147	4220738	5471667	2666672
811	N-Oleoylethanolamine	$C_9H_{11}NO_3$	$[M+H]^+$	Alkaloids	843118	219203	203662	223221	207262
812	Imidazole-4-Acetic Acid*	$C_{11}H_{14}N_2O_3$	$[M+H]^+$	Alkaloids	355209	369099	326037	278142	504356
813	N-Isopentenyl-6-hydroxydendroxinium	$C_9H_{11}NO_3$	[M]+	Alkaloids	43961	55477	40341	51112	44448
814	10-Formyltetrahydrofolic Acid	$C_{11}H_{14}N_2O_3$	$[M+H]^+$	Alkaloids	317441	273802	246104	298532	175722
815	Nicotianamine	$C_9H_{11}NO_3$	$[M+H]^+$	Alkaloids	264561	292214	190900	162584	84440
816	Dendrobin A	$C_{11}H_{14}N_2O_3$	$[M+H]^+$	Alkaloids	5013534	8628759	8046617	7513748	6437899
817	Spermine	$C_9H_{11}NO_3$	$[M+H]^+$	Alkaloids	5187317	6949988	9163109	5246914	6504102
818	L-Azetidine-2-carboxylic acid	$C_{11}H_{14}N_2O_3$	$[M+H]^+$	Alkaloids	299384	248939	241844	227741	125186
819	m-Aminophenylacetylene	$C_9H_{11}NO_3$	$[M+H]^+$	Alkaloids	97318	52665	64880	79781	38914
820	Spermidine	$C_{11}H_{14}N_2O_3$	$[M+H]^+$	Alkaloids	12486	13381	15711	9	9
821	Imidazol-1-yl-acetic acid*	$C_9H_{11}NO_3$	$[M+H]^+$	Alkaloids	505349	477601	377921	453821	404129
822	O-Phosphorylethanolamine	$C_{11}H_{14}N_2O_3$	[M-H]-	Alkaloids	1868	28947	54873	58416	50285
823	DL-2-Aminoadipic acid	$C_9H_{11}NO_3$	$[M+H]^+$	Alkaloids	793043	543073	533748	691877	669264
824	2(3H)-Benzothiazolone	$C_{11}H_{14}N_2O_3$	[M-H]-	Alkaloids	14509	16544	19335	14851	16217
825	(E)-Cinnamamide	$C_9H_{11}NO_3$	$[M+H]^+$	Alkaloids	9	17379	27305	18183	19483
826	2-Benzoxazolinone	$C_{11}H_{14}N_2O_3$	$[M+H]^+$	Alkaloids	9	11362	15246	15021	64244
827	Lumichrome	$C_9H_{11}NO_3$	$[M+H]^+$	Alkaloids	31210	36139	29412	40123	19906
828	3-Dehydro-L-Threonic Acid*	$C_{11}H_{14}N_2O_3$	[M-H]-	Others	82944140	124615512	92293778	104044935	126848596
829	D-Mannose*	$C_9H_{11}NO_3$	[M-H]-	Others	25332971	40548940	38046145	34851309	39354400

830	D-Glucosamine	$C_{11}H_{14}N_2O_3$	$[M+H]^+$	Others	3065754	3802284	3176561	3600843	3077281
831	D-Sucrose*	$C_9H_{11}NO_3$	[M-H]-	Others	632794	2506363	2500975	2305366	3703231
832	D-Galactaric acid*	$C_{11}H_{14}N_2O_3$	[M-H]-	Others	1167054	777204	680833	747384	607997
833	N-trans-ferulic tyramine	$C_9H_{11}NO_3$	$[M+H]^+$	Others	11112085	12756380	16272710	8999255	13020371
834	D-Panthenol	$C_{11}H_{14}N_2O_3$	$[M+H]^+$	Others	1306607	174679	125645	74606	102804
835	D-Ribose	$C_9H_{11}NO_3$	[M-H]-	Others	451156	325823	497729	667002	364232
836	D-Glucose 6-phosphate*	$C_{11}H_{14}N_2O_3$	[M-H]-	Others	791768	3143226	3212522	3210091	3092288
837	D-Fructose 6-Phosphate*	$C_9H_{11}NO_3$	[M-H]-	Others	795040	2618093	2706718	2384406	2107586
838	sorbose	$C_{11}H_{14}N_2O_3$	[M-H]-	Others	23977177	37730702	36830080	33259643	37091583
839	D-Maltotetraose	$C_9H_{11}NO_3$	[M-H]-	Others	113135	351215	185965	217353	141431
840	D-Threose	$C_{11}H_{14}N_2O_3$	$[M+H]^+$	Others	354345	615800	758999	758997	269617
841	Ribitol*	$C_9H_{11}NO_3$	[M-H]-	Others	568355	151956	219304	142712	87015
842	Xylitol*	$C_{11}H_{14}N_2O_3$	[M-H]-	Others	568355	151956	219304	142712	87015
843	D-Trehalose*	$C_9H_{11}NO_3$	[M-H]-	Others	679407	1592317	1494817	1387408	1755767
844	2,3-Dihydroxypropanal	$C_{11}H_{14}N_2O_3$	[M-H]-	Others	39710	27905	305166	343719	28532
845	1,6-anhydro-β-D-glucose	$C_9H_{11}NO_3$	[M-H]-	Others	1421460	1403577	1191871	1068921	1073986
846	Glucose-1-phosphate*	$C_{11}H_{14}N_2O_3$	[M-H]-	Others	844724	3322262	3251556	3383177	3050557
847	D-Galactose*	$C_9H_{11}NO_3$	[M-H]-	Others	13626786	26914859	23162525	21361402	26633789
848	Pantothenol	$C_{11}H_{14}N_2O_3$	[M-H]-	Others	95239	16504	9693	10260	13726
849	Trehalose 6-phosphate	$C_9H_{11}NO_3$	[M-H]-	Others	106263	252366	228787	224643	317652
850	D-Sorbitol	$C_{11}H_{14}N_2O_3$	[M-H]-	Others	1913803	411419	3961135	3627399	299595
851	1,5-Anhydro-D-glucitol	$C_9H_{11}NO_3$	[M-H]-	Others	290181	234977	210770	224406	219752
852	Isomaltulose*	$C_{11}H_{14}N_2O_3$	[M-H]-	Others	1252285	3260935	2814051	2751753	3068876
853	N-Acetyl-D-glucosamine-1-phosphate	$C_9H_{11}NO_3$	[M-H]-	Others	75618	43438	63843	47281	35255
854	Maltotriose	$C_{11}H_{14}N_2O_3$	$[M+Na]^+$	Others	107965	225989	114161	116628	122657
855	D-Fructose*	$C_9H_{11}NO_3$	[M-H]-	Others	24022843	36228222	33867029	31186326	36339381
856	3-Phospho-D-glyceric acid	$C_{11}H_{14}N_2O_3$	[M-H]-	Others	115972	173041	169499	144291	48623

857	Maltitol	$C_9H_{11}NO_3$	[M-H]-	Others	111703	114928	117297	110119	104753
858	Glucarate O-Phosphoric acid	$C_{11}H_{14}N_2O_3$	[M-H]-	Others	2513154	588905	1236199	782216	565448
859	D-Glucosamine 1-phosphate	$C_9H_{11}NO_3$	[M-H]-	Others	13709	40311	89300	115090	12033
860	N-Acetyl-D-galactosamine	$C_{11}H_{14}N_2O_3$	$[M+H]^+$	Others	223056	147042	171448	269681	230837
861	Inositol*	$C_9H_{11}NO_3$	[M-H]-	Others	23976122	36620181	35030553	31456380	35330439
862	D-Galacturonic acid*	$C_{11}H_{14}N_2O_3$	[M-H]-	Others	2731714	1490487	7284116	3776316	534142
863	D-Xylonic acid	$C_9H_{11}NO_3$	[M-H]-	Others	19757263	29534633	33052559	31003428	35883487
864	D-Glucose*	$C_{11}H_{14}N_2O_3$	[M-H]-	Others	25828681	38168304	36066324	31593651	36695004
865	Stachyose	$C_9H_{11}NO_3$	[M-H]-	Others	32235	182893	110596	125194	75237
866	Galactinol	$C_{11}H_{14}N_2O_3$	[M-H]-	Others	1222368	4486249	5157653	4196414	7230391
867	D-Arabitol*	$C_9H_{11}NO_3$	[M-H]-	Others	2200594	149868	320439	491259	84805
868	Sedoheptulose	$C_{11}H_{14}N_2O_3$	[M-H]-	Others	1084767	681028	578809	622364	462179
869	DL-Xylose	$C_9H_{11}NO_3$	[M-H]-	Others	383766	490909	485607	473304	512907
870	D-Mannitol*	$C_{11}H_{14}N_2O_3$	[M-H]-	Others	641091	110036	1391631	1291294	75926
871	D-Sedoheptuiose 7-phosphate	$C_9H_{11}NO_3$	[M-H]-	Others	1239109	358713	913205	626833	534233
872	L-Fucitol	$C_{11}H_{14}N_2O_3$	[M-H]-	Others	2407388	4734974	5298522	4921029	5268780
873	D-Melezitose	$C_9H_{11}NO_3$	[M-H]-	Others	25698	77098	91033	71651	115228
874	D-Saccharic acid*	$C_{11}H_{14}N_2O_3$	[M-H]-	Others	1200477	830571	705921	755134	648771
875	D-Glucoronic acid*	$C_9H_{11}NO_3$	[M-H]-	Others	2970755	1710430	8083666	3709063	553114
876	N-Acetyl-D-mannosamine	$C_{11}H_{14}N_2O_3$	$[M+H]^+$	Others	205530	126106	94927	78613	37938
877	Gluconic acid	$C_9H_{11}NO_3$	[M-H]-	Others	18599832	4620462	4167175	6332270	2721121
878	Melibiose	$C_{11}H_{14}N_2O_3$	[M-H]-	Others	796098	1232773	1166940	1090614	1475192
879	D-Threonic Acid	$C_9H_{11}NO_3$	[M-H]-	Others	2267691	2577698	2025456	2111806	2818881
880	D-Erythrose-4-phosphate	$C_{11}H_{14}N_2O_3$	[M-H]-	Others	23816	28834	95603	140955	20964
881	Dulcitol*	$C_9H_{11}NO_3$	[M-H]-	Others	2284114	377644	4637352	4634736	267094
882	D-Panose*	$C_{11}H_{14}N_2O_3$	[M-H]-	Others	220515	268566	246156	274056	169787
883	D-Glucurono-6,3-lactone	$C_9H_{11}NO_3$	[M-H]-	Others	86880	119293	133073	138991	115375
884	Raffinose*	$C_{11}H_{14}N_2O_3$	[M-H]-	Others	168675	201576	171991	180300	162623

885	D-Cellobiose	$C_9H_{11}NO_3$	[M-H]-	Others	381489	452445	366207	353664	229609
886	Manninotriose	$C_{11}H_{14}N_2O_3$	[M-H]-	Others	155576	119948	108980	117493	38285
887	D-Maltose*	$C_9H_{11}NO_3$	[M-H]-	Others	1175860	3032176	2667334	2396845	2844736
888	D-Glucose 1,6-bisphosphate	$C_{11}H_{14}N_2O_3$	[M-H]-	Others	5802	10371	10873	14033	12414
889	D-Arabinose	$C_9H_{11}NO_3$	[M-H]-	Others	411667	546601	574493	480197	575691
890	D-Pinitol	$C_{11}H_{14}N_2O_3$	[M-H]-	Others	3615966	2383662	7415562	9520922	982329
891	D-Arabinono-1,4-lactone*	$C_9H_{11}NO_3$	[M-H]-	Others	479748	264057	259428	323475	285657
892	Rutinose	$C_{11}H_{14}N_2O_3$	[M-H]-	Others	273386	222541	213816	229707	120313
893	Sucrose-6-phosphate	$C_9H_{11}NO_3$	[M-H]-	Others	63133	128601	142087	147603	212029
894	Lactobiose	$C_{11}H_{14}N_2O_3$	[M-H]-	Others	88259	532857	614258	420814	1269946
895	Verbascose	$C_9H_{11}NO_3$	[M-H]-	Others	14083	154338	84649	125831	64540
896	Sorbitol-6-phosphate	$C_{11}H_{14}N_2O_3$	[M-H]-	Others	26546	30196	49115	56682	34427
897	Blumenol C glucoside; Byzantionoside B	$C_9H_{11}NO_3$	$[M+H]^+$	Terpenoids	69185	61112	54296	64599	56409
898	Dehydrololiolide	$C_{11}H_{14}N_2O_3$	[M-H]-	Terpenoids	1392650	4661853	3911913	3395320	2411768
899	Blumenol C	$C_9H_{11}NO_3$	$[M+H]^+$	Terpenoids	41902	37279	32340	39537	9
900	α-Cyperone	$C_{11}H_{14}N_2O_3$	$[M+H]^+$	Others	32851	32017	34503	32580	38605
901	Dengibsin	$C_9H_{11}NO_3$	[M-H]-	Others	603660	1001403	1909923	1405629	2079862
902	Cis-Jasmone	$C_{11}H_{14}N_2O_3$	[M-H]-	Others	2183	15760	6842	5644	6571
903	Roseoside	$C_9H_{11}NO_3$	[M-H]-	Others	79395	89746	216742	104146	9
904	Acetosyringone	$C_{11}H_{14}N_2O_3$	[M-H]-	Others	22582	21673	16781	26253	24383
905	Orotic acid (Vitamin B13)	$C_9H_{11}NO_3$	[M-H]-	Others	471448	161507	291430	296751	396248
906	Pyridoxal	$C_{11}H_{14}N_2O_3$	$[M+H]^+$	Others	107945	51725	40988	34522	98456
907	Pyridoxine	$C_9H_{11}NO_3$	$[M+H]^+$	Others	4315621	780860	550282	723148	459129
908	Isonicotinic acid	$C_{11}H_{14}N_2O_3$	$[M+H]^+$	Others	1337842	383153	451551	508797	239618
909	Nicotinamide	$C_9H_{11}NO_3$	$[M+H]^+$	Others	502722	6462863	6006734	7265063	7961674
910	Nicotinic acid (Vitamin B3)*	$C_{11}H_{14}N_2O_3$	$[M+H]^+$	Others	2454194	556119	694257	668680	354491
911	Pyridoxine-5'-O-glucoside	$C_9H_{11}NO_3$	$[M+H]^+$	Others	145266	453539	351560	321110	321281

912	Riboflavin (Vitamin B2)	$C_{11}H_{14}N_2O_3$	$[M+H]^+$	Others	650606	777298	605355	809097	594298
913	Menatetrenone (Vitamin K2)	$C_9H_{11}NO_3$	$[M+H]^+$	Others	97617	145858	117174	140421	209993
914	Nicotinate D-ribonucleoside	$C_{11}H_{14}N_2O_3$	[M]+	Others	4192336	4276257	2612119	3732774	720266
915	4-Pyridoxic acid	$C_9H_{11}NO_3$	$[M+H]^+$	Others	77595	50312	33935	53510	37309
916	D-Pantothenic Acid	$C_{11}H_{14}N_2O_3$	[M-H]-	Others	1499740	1567709	1452262	1553594	1002127
917	Erythorbic Acid; Isoascorbic Acid	$C_9H_{11}NO_3$	[M-H]-	Others	528708	1089797	1154178	1405867	1778952
918	4-Oxoretinol	$C_{11}H_{14}N_2O_3$	$[M+H]^+$	Others	10153	6426	3422	5379	5766
919	Dehydroascorbic acid	$C_9H_{11}NO_3$	[M-H]-	Others	729154	2150834	2364585	1548339	1746690
920	Delta-Tocopherol	$C_{11}H_{14}N_2O_3$	$[M+H]^+$	Others	4970	10644	9594	11386	31948
921	N-(beta-D-Glucosyl)nicotinate	$C_9H_{11}NO_3$	$[M+H]^+$	Others	61454	55585	52641	56968	41576
922	Daphnin*	$C_{11}H_{14}N_2O_3$	[M-H]-	Lignans and Coumarins	125899	148544	195682	229514	88937
923	Scopoletin-7-O-glucoside (Scopolin)	$C_9H_{11}NO_3$	$[M+H]^+$	Lignans and Coumarins	55497	37915	26421	40181	23654
924	Esculin (6,7-Dihydroxycoumarin-6-O-glucoside)*	$C_{11}H_{14}N_2O_3$	[M-H]-	Lignans and Coumarins	152843	142215	207123	244751	93270
925	Isofraxidin-7-O-glucoside	$C_9H_{11}NO_3$	$[M+H]^+$	Lignans and Coumarins	29712	48949	64054	38069	69750
926	Oxypeucedanin	$C_{11}H_{14}N_2O_3$	$[M+H]^+$	Lignans and Coumarins	17487	39685	39190	38250	26802
927	Osthole	$C_9H_{11}NO_3$	$[M+H]^+$	Lignans and Coumarins	9168	11803	14905	9112	14827
928	Esculetin-7-O-glucoside*	$C_{11}H_{14}N_2O_3$	[M-H]-	Lignans and Coumarins	127195	136817	163953	223951	68625
929	Fraxidin-8-O-glucoside	$C_9H_{11}NO_3$	$[M+H]^+$	Lignans and Coumarins	32265	65174	70764	42109	62244

930	6,7-Dihydroxy-4-methylcoumarin	$C_{11}H_{14}N_2O_3$	$[M+H]^+$	Lignans and Coumarins	395349	468087	431470	675969	460299
931	Nodakenin	$C_9H_{11}NO_3$	[M-H]-	Lignans and Coumarins	1030058	1430060	1265406	1183635	1147998
932	Esculetin (6,7-Dihydroxycoumarin)	$C_{11}H_{14}N_2O_3$	[M-H]-	Lignans and Coumarins	276711	490170	794944	705532	223152
933	Fraxetin (7,8-Dihydroxy-6-methoxycoumarin)	$C_9H_{11}NO_3$	$[M+H]^+$	Lignans and Coumarins	129035	181744	185142	157801	167685
934	isofraxidin	$C_{11}H_{14}N_2O_3$	$[M+H]^+$	Lignans and Coumarins	30869	36927	24113	22470	17908
935	7,8-Dihydroxy-4-phenylcoumarin	$C_9H_{11}NO_3$	$[M+H]^+$	Lignans and Coumarins	9899	8014	11000	30904	34347
936	4-Hydroxycoumarin	$C_{11}H_{14}N_2O_3$	[M-H]-	Lignans and Coumarins	24978	69612	86539	79723	122332
937	Ayapin	$C_9H_{11}NO_3$	[M-H]-	Lignans and Coumarins	913814	795881	695973	860641	561446
938	5,7-dimethoxy-8-hydroxycoumarin	$C_{11}H_{14}N_2O_3$	$[M+H]^+$	Lignans and Coumarins	17145	18006	30279	15384	15428
939	Daphnetin	$C_9H_{11}NO_3$	[M-H]-	Lignans and Coumarins	33449	72044	106773	109394	27399
940	Isoquinoline	$C_{11}H_{14}N_2O_3$	$[M+H]^+$	Alkaloids	141173	121084	129675	152138	65941
941	1-Methoxy-indole-3-acetamide	$C_9H_{11}NO_3$	$[M+H]^+$	Alkaloids	32157124	17955815	18545135	21738941	12086011
942	1-Acetyl-β-carboline	$C_{11}H_{14}N_2O_3$	$[M+H]^+$	Alkaloids	17010	27273	46322	86564	148739
943	Methoxyindoleacetic acid	$C_9H_{11}NO_3$	$[M+H]^+$	Alkaloids	613795	365189	357220	451654	250423
944	3-Indoleacrylic acid	$C_{11}H_{14}N_2O_3$	$[M+H]^+$	Alkaloids	10074834	5211230	5415459	6705959	3435553
945	Indole-3-carboxaldehyde	$C_9H_{11}NO_3$	$[M+H]^+$	Alkaloids	114623	181796	125786	238000	198843
946	Tryptamine	$C_{11}H_{14}N_2O_3$	$[M+H]^+$	Alkaloids	134350	81994	139223	96613	108205

947	1-Methoxycarbonyl-β-Carboline-N-Oxide	$C_9H_{11}NO_3$	$[M+H]^+$	Alkaloids	40281	81862	64226	56990	39629
948	N-Acetyl-5-hydroxytryptamine	$C_{11}H_{14}N_2O_3$	$[M+H]^+$	Alkaloids	37825	27714	25384	19832	28710
949	Indole	$C_9H_{11}NO_3$	$[M+H]^+$	Alkaloids	48640	97571	83663	84068	57344
950	N-Feruloyltryptamine	$C_{11}H_{14}N_2O_3$	$[M+H]^+$	Alkaloids	26814	62487	49443	68649	38185
951	Indole-3-carboxylic acid*	$C_9H_{11}NO_3$	[M-H]-	Alkaloids	477124	209579	231906	393152	174577
952	Indole-5-carboxylic acid*	$C_{11}H_{14}N_2O_3$	[M-H]-	Alkaloids	274851	149623	151089	221380	118529
953	3-Indoleacetonitrile	$C_9H_{11}NO_3$	$[M+H]^+$	Alkaloids	232302	201923	178441	263729	160947
954	Acetryptine	$C_{11}H_{14}N_2O_3$	$[M+H]^+$	Alkaloids	60978	16716	22787	22767	13788
955	Indole-3-acetic acid (IAA)	$C_9H_{11}NO_3$	$[M+H]^+$	Alkaloids	29734	11835	17197	20328	13308
956	12-Oxo-phytodienoic acid	$C_{11}H_{14}N_2O_3$	[M-H]-	Lipids	291344	75915	111902	181270	20054
957	9-Oxo-10E,12Z-octadecadienoic acid	$C_9H_{11}NO_3$	[M-H]-	Lipids	1200754	205193	147538	234899	79847
958	9-Hydroperoxy-9Z,11E-Octadecadienoic Acid	$C_{11}H_{14}N_2O_3$	[M-H]-	Lipids	2183978	560878	394764	635824	151688
959	(7Z)-Hexadecenoic acid*	$C_9H_{11}NO_3$	[M-H]-	Lipids	66171	25437	38381	66580	22977
960	Ricinoleic acid	$C_{11}H_{14}N_2O_3$	[M-H]-	Lipids	395230	102694	202213	266988	86433
961	9,10-Dihydroxy-12,13-epoxyoctadecanoic acid	$C_9H_{11}NO_3$	[M-H]-	Lipids	312387	73894	56651	68737	47616
962	Crepenynic acid	$C_{11}H_{14}N_2O_3$	[M-H]-	Lipids	889079	645259	795699	1815427	353807
963	9-Oxo-12Z-Octadecenoic acid*	$C_9H_{11}NO_3$	$[M+H]^+$	Lipids	2793235	554509	733194	1046756	386495
964	Oleamide (9-Octadecenamide)	$C_{11}H_{14}N_2O_3$	$[M+H]^+$	Lipids	104231	166582	401683	211489	91626
965	Punicic acid (9Z,11E,13Z-octadecatrienoic acid)	$C_9H_{11}NO_3$	$[M+H]^+$	Lipids	30173373	12710050	8457982	13805181	3281365
966	9(10)-EpOME;(9R,10S)-(12Z)	$C_{11}H_{14}N_2O_3$	[M-H]-	Lipids	5143610	888942	556282	700602	428265

	-9,10-Epoxyoctadecenoic acid								
967	15(R)-Hydroxylinoleic Acid*	$C_9H_{11}NO_3$	$[M+H]^+$	Lipids	2658237	550540	711228	1030379	351423
968	Octadeca-11E,13E,15Z-trienoic acid	$C_{11}H_{14}N_2O_3$	$[M+H]^+$	Lipids	31973740	35069916	36180410	35824262	37462654
969	9,10,18-Trihydroxystearic acid	$C_9H_{11}NO_3$	[M-H]-	Lipids	15840	41403	44739	58763	40404
970	9-Hydroxy-12-oxo-10(E),15(Z)-octadecadienoic acid	$C_{11}H_{14}N_2O_3$	[M-H]-	Lipids	289001	61741	42690	71669	35092
971	12,13-DHOME; (9Z)-12,13-Dihydroxyoctadec-9-enoic acid	$C_9H_{11}NO_3$	[M-H]-	Lipids	155708	181879	168421	184306	144396
972	Eicosadienoic acid	$C_{11}H_{14}N_2O_3$	[M-H]-	Lipids	24708216	16214336	18890163	17172712	6620497
973	3-Hydroxyoctadecanoic Acid	$C_9H_{11}NO_3$	[M-H]-	Lipids	67607	50884	57275	75259	50772
974	Methyl linolenate	$C_{11}H_{14}N_2O_3$	$[M+H]^+$	Lipids	2758991	977515	2479599	3811079	69959
975	13S-Hydroxy-9Z,11E,15Z-octadecatrienoic acid	$C_9H_{11}NO_3$	[M-H]-	Lipids	2338445	805406	600335	1130715	189665
976	12-Hydroxyoctadecanoic acid	$C_{11}H_{14}N_2O_3$	[M-H]-	Lipids	59413	42495	29812	26234	45493
977	12,13-Epoxy-9-Octadecenoic Acid	$C_9H_{11}NO_3$	[M-H]-	Lipids	2225408	465761	291868	402831	237856
978	9-Hydroxy-12-oxo-15(Z)-octadecenoic acid	$C_{11}H_{14}N_2O_3$	[M-H]-	Lipids	3428805	162358	140035	176201	148309
979	Palmitaldehyde	$C_9H_{11}NO_3$	[M-H]-	Lipids	46008744	52744653	50363524	54424020	54467745
980	13S-Hydroperoxy-9Z,11E-octadecadienoic acid	$C_{11}H_{14}N_2O_3$	[M-H]-	Lipids	5285378	1291969	822806	1115174	714804
981	13(S)-HODE;13(S)-Hydroxyoctadeca-9Z,11E-dienoic acid*	$C_9H_{11}NO_3$	[M-H]-	Lipids	18149916	9820037	6364908	10510419	2399589
982	Hydroperoxylinoleic acid	$C_{11}H_{14}N_2O_3$	[M-H]-	Lipids	3613552	174495	132814	189591	156634
983	9,12,13-Trihydroxy-10,15-octadecadienoic acid	$C_9H_{11}NO_3$	[M-H]-	Lipids	689679	109105	92729	162818	46302

984	Hydroxy ricinoleic acid	$C_{11}H_{14}N_2O_3$	[M-H]-	Lipids	5494271	395381	278057	366672	322948
985	9,16-Dihydroxypalmitic acid	$C_9H_{11}NO_3$	[M-H]-	Lipids	998131	2269233	1879301	2445489	2229508
986	5S,8R-DiHODE; (5S,8R,9Z,12Z)-5,8-Dihydroxy octadeca-9,12-dienoate	$C_{11}H_{14}N_2O_3$	[M-H]-	Lipids	2566789	71362	45736	35204	35215
987	α-Linolenic Acid*	$C_9H_{11}NO_3$	[M-H]-	Lipids	42914263	33777205	43972933	41929568	19241020
988	1-Octadecanol	$C_{11}H_{14}N_2O_3$	[M-H]-	Lipids	40767	39754	39245	39708	40870
989	Palmitoleic Acid*	$C_9H_{11}NO_3$	[M-H]-	Lipids	153825	86758	98186	133031	77709
990	13-KODE; (9Z,11E)-13-Oxooctadeca-9,11 -dienoic acid*	$C_{11}H_{14}N_2O_3$	[M-H]-	Lipids	1011178	117769	61950	90546	41945
991	γ-Linolenic Acid*	$C_9H_{11}NO_3$	[M-H]-	Lipids	47028651	35392292	50574292	57705634	20243273
992	Undecylic Acid	$C_{11}H_{14}N_2O_3$	[M-H]-	Lipids	508920	896389	630573	717551	764610
993	7S,8S-DiHODE; (9Z,12Z)-(7S,8S)-Dihydroxyoc tadeca-9,12-dienoic acid*	$C_9H_{11}NO_3$	[M-H]-	Lipids	233383	231943	209893	280647	262514
994	Dibutyl sebacate	$C_{11}H_{14}N_2O_3$	$[M+H]^+$	Lipids	19681	12589	11431	17183	8736
995	1-Eicosanol	$C_9H_{11}NO_3$	[M-H]-	Lipids	383312	103801	193806	252117	87174
996	alpha-Hydroxylinoleic acid	$C_{11}H_{14}N_2O_3$	[M-H]-	Lipids	18043020	9319752	6316310	10323691	2254827
997	2R-Hydroxyoctadecanoic Acid*	$C_9H_{11}NO_3$	[M-H]-	Lipids	12883416	2388183	2516790	3143243	3035510
998	(9Z,11E)-Octadecadienoic acid*	$C_{11}H_{14}N_2O_3$	[M-H]-	Lipids	303054	256262	259142	340223	148431
999	Linoleic acid*	$C_9H_{11}NO_3$	[M-H]-	Lipids	303054	256262	259142	340223	148431
1000	9-Hydroxy-10,12,15-octadecat rienoic acid*	$C_{11}H_{14}N_2O_3$	[M-H]-	Lipids	947883	111144	60773	90045	41920
1001	Pentadecanoic Acid	$C_9H_{11}NO_3$	[M-H]-	Lipids	15454616	12157821	14810174	17076330	7080331
1002	9,10,11-Trihydroxy-12-octadec	$C_{11}H_{14}N_2O_3$	[M-H]-	Lipids	700158	160570	145677	198719	111013

	enoic acid								
1003	DL-2-hydroxystearic acid*	$C_9H_{11}NO_3$	[M-H]-	Lipids	13142382	2969181	2795804	3404858	3015789
1004	9-Hydroperoxy-10E,12,15Z-octadecatrienoic acid	$C_{11}H_{14}N_2O_3$	[M-H]-	Lipids	531728	216714	195427	385073	63266
1005	Hexadecanedioic acid	$C_9H_{11}NO_3$	[M-H]-	Lipids	241025	191840	170850	189766	216389
1006	9-Hydroxy-13-oxo-10-octadecenoic Acid	$C_{11}H_{14}N_2O_3$	[M-H]-	Lipids	1044922	381813	361930	494588	248786
1007	13-Hydroperoxy-9Z,11E-octadecadienoic acid*	$C_9H_{11}NO_3$	[M-H]-	Lipids	292907	77924	54549	89564	20462
1008	9S-Hydroxy-10E,12Z-octadecadienoic acid*	$C_{11}H_{14}N_2O_3$	[M-H]-	Lipids	18501345	9777353	6448379	10959500	2360562
1009	17-Hydroxylinolenic acid	$C_9H_{11}NO_3$	[M-H]-	Lipids	5565524	2065899	1519987	2614876	825136
1010	13-methylmyristic acid	$C_{11}H_{14}N_2O_3$	[M-H]-	Lipids	438592	286638	352836	506486	342288
1011	2R-hydroxy-9Z,12Z,15Z-octadecatrienoic acid	$C_9H_{11}NO_3$	[M-H]-	Lipids	1388665	652998	285323	877816	179079
1012	13(s)-hydroperoxy-(9z,11e,15z)-octadecatrienoic acid	$C_{11}H_{14}N_2O_3$	[M-H]-	Lipids	1447284	955924	891067	913270	414525
1013	9,10,13-Trihydroxy-11-Octadecenoic Acid	$C_9H_{11}NO_3$	[M-H]-	Lipids	5904261	1644962	1281892	1808278	1020157
1014	Tridecanedioic acid	$C_{11}H_{14}N_2O_3$	[M-H]-	Lipids	133952	142986	120861	128254	166648
1015	9,10-DHOME; (12Z)-9,10-Dihydroxyoctadec-12-enoic acid	$C_9H_{11}NO_3$	[M-H]-	Lipids	30300	1874	6885	9535	9
1016	11-Octadecanoic acid(Vaccenic acid)*	$C_{11}H_{14}N_2O_3$	[M-H]-	Lipids	62203053	52072690	55531449	67648637	28591159
1017	1,14-Tetradecanedioic Acid	$C_9H_{11}NO_3$	[M-H]-	Lipids	33876	41485	38355	43933	45149
1018	Cis-4,7,10,13,16,19-Docosahexaenoic Acid	$C_{11}H_{14}N_2O_3$	[M-H]-	Lipids	9801	8297	9762	12781	6019

1019	Heptadecanoic acid	$C_9H_{11}NO_3$	[M-H]-	Lipids	902629	584131	726096	711171	656888
1020	Dodecanoic acid (Lauric acid)	$C_{11}H_{14}N_2O_3$	[M-H]-	Lipids	140679	135853	154771	174468	101238
1021	Elaidic Acid	$C_9H_{11}NO_3$	[M-H]-	Lipids	61117010	51474096	55235026	67556542	27273848
1022	Petroselinic acid*	$C_{11}H_{14}N_2O_3$	[M-H]-	Lipids	61117010	51474096	55235026	67556542	27273848
1023	1-Monomyristin	$C_9H_{11}NO_3$	$[M+H]^+$	Lipids	503991	330793	224345	237180	296390
1024	Undecanedioic acid	$C_{11}H_{14}N_2O_3$	[M-H]-	Lipids	528209	350901	270759	427284	269896
1025	Tridecanoic Acid	$C_9H_{11}NO_3$	[M-H]-	Lipids	8109	8280	8688	10258	5121
1026	3-Hydroxy-palmitic acid methyl ester	$C_{11}H_{14}N_2O_3$	[M-H]-	Lipids	1878729	474612	408045	580676	384625
1027	Dodecanedioic aicd	$C_9H_{11}NO_3$	[M-H]-	Lipids	3646	4483	5049	4560	4993
1028	Cis-10-Pentadecenoic Acid(C15:1)	$C_{11}H_{14}N_2O_3$	[M-H]-	Lipids	741053	898654	897064	976870	1352643
1029	Stearic Acid	$C_9H_{11}NO_3$	[M-H]-	Lipids	2454340	3389474	2945055	3013322	3547743
1030	Palmitic acid	$C_{11}H_{14}N_2O_3$	[M-H]-	Lipids	138996	138218	166897	184158	129577
1031	16-Methylheptadecanoic acid	$C_9H_{11}NO_3$	[M-H]-	Lipids	2141296	1795851	2901793	3053407	3495758
1032	N-(2-Hydroxyethyl)eicosapentaenoic acid	$C_{11}H_{14}N_2O_3$	$[M+H]^+$	Lipids	19012	7981	8740	8858	17869
1033	10,16-Dihydroxypalmitic acid	$C_9H_{11}NO_3$	[M-H]-	Lipids	38970	80401	65148	77552	68685
1034	4-Oxo-9Z,11Z,13E,15E-Octadecatetraenoic Acid	$C_{11}H_{14}N_2O_3$	$[M+H]^+$	Lipids	33256	9618	5343	9	9
1035	Docosanoic acid (Behenic acid)	$C_9H_{11}NO_3$	[M-H]-	Lipids	57428	59090	47048	47284	26178
1036	Arachidonic Acid	$C_{11}H_{14}N_2O_3$	[M-H]-	Lipids	50116	19340	18861	22342	25011
1037	10-Undecenoic acid	$C_9H_{11}NO_3$	[M-H]-	Lipids	26414	25458	22052	29036	25456
1038	Arachidic acid	$C_{11}H_{14}N_2O_3$	[M-H]-	Lipids	5860806	8691944	5776959	5100281	4441356
1039	13-Hydroxy-9Z,11E-octadecadienoic acid	$C_9H_{11}NO_3$	$[M+H]^+$	Lipids	4879	1978	17878	27068	9
1040	Dibutyl phthalate	$C_{11}H_{14}N_2O_3$	$[M+H]^+$	Organic acids	24541876	26760353	27369190	26562642	27983012

1041	6-Aminocaproic acid	$C_9H_{11}NO_3$	$[M+H]^+$	Organic acids	1115538	709347	697520	1186685	494959
1042	2-Aminoisobutyric acid*	$C_{11}H_{14}N_2O_3$	$[M+H]^+$	Organic acids	3314180	2580579	1610561	1968365	1188642
1043	Methylmalonic acid*	$C_9H_{11}NO_3$	[M-H]-	Organic acids	62652744	9645805	42085408	57612252	10153761
1044	L-Malic acid*	$C_{11}H_{14}N_2O_3$	[M-H]-	Organic acids	3580105	4705866	3541145	3809937	4504682
1045	γ-Aminobutyric acid	$C_9H_{11}NO_3$	$[M+H]^+$	Organic acids	16531498	9613042	9270526	12356928	4191699
1046	Citric Acid	$C_{11}H_{14}N_2O_3$	[M-H]-	Organic acids	134798734	66658123	92942285	103801687	96922529
1047	Methanesulfonic acid	$C_9H_{11}NO_3$	[M-H]-	Organic acids	309441	265482	232132	194677	196375
1048	2-Hydroxyethylphosphonic acid	$C_{11}H_{14}N_2O_3$	[M-H]-	Organic acids	947463	9	175280	159690	9
1049	2,3-Dihydroxy-3-Methylbutanoic Acid	$C_9H_{11}NO_3$	[M-H]-	Organic acids	46731490	73981609	55007277	57362247	73544470
1050	Trans-Citridic acid	$C_{11}H_{14}N_2O_3$	$[M+H]^+$	Organic acids	1339594	2125095	1996785	1821548	1839757
1051	2-Methylglutaric acid*	$C_9H_{11}NO_3$	[M-H]-	Organic acids	539203	472067	581161	723872	355969
1052	Adipic Acid*	$C_{11}H_{14}N_2O_3$	[M-H]-	Organic acids	539203	472067	581161	723872	355969
1053	4-Acetamidobutyric acid	$C_9H_{11}NO_3$	$[M+H]^+$	Organic acids	10673912	930349	1134768	2112412	1266515
1054	2-Hydroxyisobutyric acid*	$C_{11}H_{14}N_2O_3$	[M-H]-	Organic acids	1047692	71446	148317	166540	35998
1055	2-Hydroxybutyric Acid*	$C_9H_{11}NO_3$	[M-H]-	Organic acids	1047692	71446	148317	166540	35998
1056	L-Pipecolic Acid	$C_{11}H_{14}N_2O_3$	$[M+H]^+$	Organic acids	794758	1026139	1906934	2274192	1707594
1057	Dihydrojasmonic acid	$C_9H_{11}NO_3$	[M-H]-	Organic acids	53224	33887	48670	54758	44661
1058	Aminomalonic acid	$C_{11}H_{14}N_2O_3$	[M-H]-	Organic acids	1372629	230320	1575294	1749026	275366
1059	Triethyl citrate	$C_9H_{11}NO_3$	$[M+H]^+$	Organic acids	159475	194605	191711	155795	168391
1060	5-Acetamidopentanoic Acid	$C_{11}H_{14}N_2O_3$	[M-H]-	Organic acids	110455	109725	100654	137138	106447
1061	2,6-Diaminooimelic acid	$C_9H_{11}NO_3$	$[M+H]^+$	Organic acids	3041489	496880	805943	705904	189762
1062	Shikimic acid	$C_{11}H_{14}N_2O_3$	[M-H]-	Organic acids	1603234	3642180	3425927	2374018	2869662
1063	Methylenesuccinic acid	$C_9H_{11}NO_3$	[M-H]-	Organic acids	105332	97896	112213	107726	75079
1064	Oxalic acid	$C_{11}H_{14}N_2O_3$	[M-H]-	Organic acids	121280	93773	1013275	1179547	50695
1065	1-Pyrroline-4-hydroxy-2-carbo	$C_9H_{11}NO_3$	[M-H]-	Organic acids	146610	262137	361359	512675	735707

	xylic acid								
1066	2-Acetyl-2-Hydroxybutanoic Acid	$C_{11}H_{14}N_2O_3$	[M-H]-	Organic acids	55228	35458	38901	60290	33980
1067	Fumaric acid*	$C_9H_{11}NO_3$	[M-H]-	Organic acids	1525083	990512	1549171	2228471	918273
1068	Maleic acid*	$C_{11}H_{14}N_2O_3$	[M-H]-	Organic acids	1525083	990512	1549171	2228471	918273
1069	2-Hydroxyhexadecanoic acid	$C_9H_{11}NO_3$	[M-H]-	Organic acids	3674619	1816018	1483966	1876019	1807384
1070	2-Picolinic acid*	$C_{11}H_{14}N_2O_3$	[M-H]-	Organic acids	1139385	334511	216170	215511	210423
1071	4-Guanidinobutyric acid	$C_9H_{11}NO_3$	$[M+H]^+$	Organic acids	1891782	1141347	1525641	2517039	1194699
1072	Hydroxypyruvic acid*	$C_{11}H_{14}N_2O_3$	[M-H]-	Organic acids	1175371	965046	721137	864052	809065
1073	L-Lactic Acid	$C_9H_{11}NO_3$	[M-H]-	Organic acids	11857	12838	89543	107809	13212
1074	2-Phosphoglycolate	$C_{11}H_{14}N_2O_3$	[M-H]-	Organic acids	36713	29262	34740	40013	38395
1075	Malonic acid	$C_9H_{11}NO_3$	[M-H]-	Organic acids	8992150	8717363	6466860	6031589	6516898
1076	Abscisic acid	$C_{11}H_{14}N_2O_3$	[M-H]-	Organic acids	67990	110545	55287	66698	62833
1077	Tranexamic Acid	$C_9H_{11}NO_3$	$[M+H]^+$	Organic acids	47214	32487	55714	67454	40728
1078	3-Hydroxybutyric acid	$C_{11}H_{14}N_2O_3$	[M-H]-	Organic acids	7355477	6888187	5219210	5605477	5327856
1079	Succinic anhydride	$C_9H_{11}NO_3$	$[M+H]^+$	Organic acids	653162	176063	617501	586977	143442
1080	Tartronate semialdehyde*	$C_{11}H_{14}N_2O_3$	[M-H]-	Organic acids	826889	660741	611256	701017	562018
1081	L-Tartaric acid	$C_9H_{11}NO_3$	[M-H]-	Organic acids	285591	186900	191928	184976	217734
1082	2-Furoic acid	$C_{11}H_{14}N_2O_3$	[M-H]-	Organic acids	317334	1613437	1883678	1559697	3967425
1083	DL-Glyceric Acid	$C_9H_{11}NO_3$	[M-H]-	Organic acids	1194703	383698	339245	447017	769091
1084	cis-Citral	$C_{11}H_{14}N_2O_3$	$[M+H]^+$	Organic acids	76551	94846	93832	110146	102491
1085	2,4-Dichlorophenoxyacetic Acid	$C_9H_{11}NO_3$	[M-H]-	Organic acids	15591	14174	12405	16843	16408
1086	1-Aminocyclopropane-1-carboxylic acid	$C_{11}H_{14}N_2O_3$	$[M+H]^+$	Organic acids	236428	213425	213033	191023	100888
1087	Glutaric acid*	$C_9H_{11}NO_3$	[M-H]-	Organic acids	1230067	267449	289453	443401	315826
1088	α-Ketoglutaric acid	$C_{11}H_{14}N_2O_3$	[M-H]-	Organic acids	90867	663906	458758	573354	293457
1089	β-Hydroxyisovaleric acid	$C_9H_{11}NO_3$	[M-H]-	Organic acids	1551198	562243	670307	935755	683864

1090	Muconic acid	$C_{11}H_{14}N_2O_3$	[M-H]-	Organic acids	367464	344069	274637	209960	480305
1091	Succinic acid*	$C_9H_{11}NO_3$	[M-H]-	Organic acids	21357314	9	33456794	41555634	7743322
1092	2-Methylsuccinic acid*	$C_{11}H_{14}N_2O_3$	[M-H]-	Organic acids	364749	377389	347601	357334	626066
1093	2-Benzylsuccinic Acid	$C_9H_{11}NO_3$	[M-H]-	Organic acids	16406	32462	47744	29700	51143
1094	δ-Guanidinovaleric acid	$C_{11}H_{14}N_2O_3$	$[M+H]^+$	Organic acids	78035	72638	67905	70603	55816
1095	3-Methyl-2-Oxobutanoic acid	$C_9H_{11}NO_3$	[M-H]-	Organic acids	228149	203282	382918	333954	207019
1096	L-Citramalic acid	$C_{11}H_{14}N_2O_3$	[M-H]-	Organic acids	1249090	209243	452342	661071	469764
1097	3-Dehydroshikimic acid	$C_9H_{11}NO_3$	[M-H]-	Organic acids	2155013	408547	364284	378976	70823
1098	Isocitric Acid	$C_{11}H_{14}N_2O_3$	[M-H]-	Organic acids	29329143	10988066	26875713	23350538	24607036
1099	6-Hydroxyhexanoic acid	$C_9H_{11}NO_3$	[M-H]-	Organic acids	640762	1375842	1037786	1229801	826338
1100	Citraconic acid	$C_{11}H_{14}N_2O_3$	[M-H]-	Organic acids	2015735	157212	183057	253254	773244
1101	1-Methylpiperidine-2-carboxylic acid	$C_9H_{11}NO_3$	$[M+H]^+$	Organic acids	2013311	2621039	1881054	2129292	2779190
1102	9-Oxononanoic acid	$C_{11}H_{14}N_2O_3$	[M-H]-	Organic acids	50933	32401	25521	36492	38993
1103	Benzoylformic acid	$C_9H_{11}NO_3$	[M-H]-	Organic acids	126039	112216	9	167645	57010
1104	Ethylmalonic acid*	$C_{11}H_{14}N_2O_3$	[M-H]-	Organic acids	132249	26236	35536	51060	34675
1105	2-Propylglutaric acid	$C_9H_{11}NO_3$	[M-H]-	Organic acids	187112	250417	279042	197897	9
1106	Succinic semialdehyde	$C_{11}H_{14}N_2O_3$	[M-H]-	Organic acids	64848	89237	80705	74886	87781
1107	2-Hydroxy-4-methylpentanoic acid	$C_9H_{11}NO_3$	[M-H]-	Organic acids	163735	53117	786783	830551	60674
1108	Mevalonic acid	$C_{11}H_{14}N_2O_3$	[M-H]-	Organic acids	427510	979684	1650372	1815049	15027658
1109	Argininosuccinic acid	$C_9H_{11}NO_3$	[M-H]-	Organic acids	61126	20002	37122	35404	19449
1110	3-Ureidopropionic Acid	$C_{11}H_{14}N_2O_3$	[M-H]-	Organic acids	717592	650532	1284359	1306590	736772
1111	Azelaic acid	$C_9H_{11}NO_3$	[M-H]-	Organic acids	14631037	12896126	10391405	13733655	9239306
1112	Pimelic acid*	$C_{11}H_{14}N_2O_3$	[M-H]-	Organic acids	81961	49629	37497	64058	35069
1113	Quinic Acid	$C_9H_{11}NO_3$	[M-H]-	Organic acids	11236740	3764564	9616506	7181017	9243557
1114	3-Guanidinopropionic acid	$C_{11}H_{14}N_2O_3$	[M-H]-	Organic acids	323719	46329	10880	56020	22256
1115	2-Aminoethanesulfonic acid	$C_9H_{11}NO_3$	[M-H]-	Organic acids	20899	9105	11977	11133	9

1116	2-Propylmalic Acid*	$C_{11}H_{14}N_2O_3$	[M-H]-	Organic acids	5707814	256365	1339562	2465959	203765
1117	3-Isopropylmalic Acid*	$C_9H_{11}NO_3$	[M-H]-	Organic acids	5707814	256365	1339562	2465959	203765
1118	2-Hydroxyisocaproic acid	$C_{11}H_{14}N_2O_3$	[M-H]-	Organic acids	1067270	432900	5096360	5305469	474289
1119	2-Hydroxyphenylacetic acid	$C_9H_{11}NO_3$	[M-H]-	Organic acids	1716210	67951	65779	72931	49170
1120	DL-Glyceraldehyde-3-phosphate	$C_{11}H_{14}N_2O_3$	[M-H]-	Organic acids	14120	6907	49509	107351	9
1121	3-Hydroxyglutaric acid*	$C_9H_{11}NO_3$	[M-H]-	Organic acids	479748	264057	259428	323475	285657
1122	Iminodiacetic acid*	$C_{11}H_{14}N_2O_3$	[M-H]-	Organic acids	4601672	892450	1210329	1480197	823980
1123	Suberic Acid	$C_9H_{11}NO_3$	[M-H]-	Organic acids	237289	310844	243718	287099	192144
1124	2-Isopropylmalic Acid	$C_{11}H_{14}N_2O_3$	[M-H]-	Organic acids	5493951	274243	1250169	2406190	200311
1125	4-Oxopentanoic Acid	$C_9H_{11}NO_3$	[M-H]-	Organic acids	17916	25208	40400	41198	23381
1126	2-Propylsuccinic acid*	$C_{11}H_{14}N_2O_3$	[M-H]-	Organic acids	80846	48073	40477	59392	33872
1127	2-Hydroxy-2-methyl-3-oxobutanoic acid	$C_9H_{11}NO_3$	[M-H]-	Organic acids	53014	45444	40365	67387	64225
1128	4,8-Dihydroxyquinoline-2-carboxylic acid	$C_{11}H_{14}N_2O_3$	[M-H]-	Organic acids	127220	79715	123307	115768	85713
1129	DL-3-Phenyllactic acid*	$C_9H_{11}NO_3$	[M-H]-	Organic acids	154493	93012	734859	700400	75746
1130	Sebacic acid	$C_{11}H_{14}N_2O_3$	[M-H]-	Organic acids	24806	5104	4879	8238	4489
1131	Pyrrole-2-carboxylic acid	$C_9H_{11}NO_3$	[M-H]-	Organic acids	25902	39048	48413	39499	174500
1132	Dimethylmalonic acid*	$C_{11}H_{14}N_2O_3$	[M-H]-	Organic acids	53258	36060	35674	41853	64348
1133	5-Aminovaleric acid	$C_9H_{11}NO_3$	$[M+H]^+$	Organic acids	261904	102755	83832	110805	46827
1134	Jasmonic acid	$C_{11}H_{14}N_2O_3$	[M-H]-	Organic acids	39483	21873	26368	25497	29519
1135	2,2-Dimethylsuccinic acid	$C_9H_{11}NO_3$	[M-H]-	Organic acids	336581	151512	169736	172176	65142
1136	D-Lactic Acid	$C_{11}H_{14}N_2O_3$	[M-H]-	Organic acids	29909	47503	39822	31636	39038
1137	Phenylpyruvic acid	$C_9H_{11}NO_3$	[M-H]-	Organic acids	43405	9	48578	45769	39502
1138	Tristin(3,4',5-Trihydroxy-3'-methoxybibenzyl)	$C_{11}H_{14}N_2O_3$	[M-H]-	Others	163491	223030	377914	238449	199641
1139	3,4'-Dihydroxy-5-methoxybibe	$C_9H_{11}NO_3$	$[M+H]^+$	Others	1346679	2706914	2216514	1996244	1283071

	nzyl								
1140	4-Hydroxy-3,5,3',5'-tetramethoxybibenzyl	$C_{11}H_{14}N_2O_3$	$[M+H]^+$	Others	2887762	4321308	6279432	3254787	4232506
1141	Dendrocandin C	$C_9H_{11}NO_3$	[M-H]-	Others	1194238	2010728	3197473	1201164	2087967
1142	3-hydroxy-5-methoxybibenzyl	$C_{11}H_{14}N_2O_3$	[M-H]-	Others	369956	666690	481937	560548	636125
1143	Crepidatin	$C_9H_{11}NO_3$	$[M+H]^+$	Others	987358	1534180	2288913	1042119	1465833
1144	4,4'-Dihydroxy-3,5-dimethoxy bibenzyl*	$C_{11}H_{14}N_2O_3$	$[M+H]^+$	Others	3379949	2812618	3797429	1805879	2741937
1145	3-hydroxy-5,3'-dimethoxybibenzyl	$C_9H_{11}NO_3$	[M-H]-	Others	1144204	2330063	1596103	1459701	953899
1146	3,4'-dihydroxy-3',5-dimethoxy bibenzyl*	$C_{11}H_{14}N_2O_3$	$[M+H]^+$	Others	2161745	1867919	2471647	1231292	1719729
1147	Batatasin III*	$C_9H_{11}NO_3$	$[M+H]^+$	Others	142237	213943	185366	200525	192883
1148	Dendrocandin A; 3,4-Dihydroxy-3',4',5-trimethoxybibenzyl*	$C_{11}H_{14}N_2O_3$	$[M+H]^+$	Others	4061098	8153360	8675945	5518564	7048759
1149	3'-Hydroxy-3,4,5'-trimethoxybibenzyl*	$C_9H_{11}NO_3$	$[M+H]^+$	Others	7028805	13071625	12549723	10646399	8993421
1150	3,4'-dihydroxy-4,5-dimethoxybibenzyl	$C_{11}H_{14}N_2O_3$	$[M+H]^+$	Others	12546418	18300838	17705702	13849972	14219290
1151	Batatasin IV*	$C_9H_{11}NO_3$	$[M+H]^+$	Others	217564	376037	325736	274750	240591
1152	3,4-Dihydroxy-5,4'-dimethoxy bibenzyl	$C_{11}H_{14}N_2O_3$	$[M+H]^+$	Others	29980307	25918629	33235960	18281085	26710565
1153	3-O-Methylgigantol*	$C_9H_{11}NO_3$	$[M+H]^+$	Others	7246341	11681440	12768564	10045543	8896612
1154	Dihydropinosylvin methyl ether	$C_{11}H_{14}N_2O_3$	$[M+H]^+$	Others	555964	838970	605801	643713	845077
1155	Moscatilin (4,4'-Dihydroxy-3,3',5-Trimeth	$C_9H_{11}NO_3$	$[[M+H]^+$	Others	4087906	8472020	8245805	5871753	7279199

	oxybibenzyl)								
1156	3',4-Dihydroxy-3,5'-dimethoxy bibenzyl*	$C_{11}H_{14}N_2O_3$	$[M+H]^+$	Others	6933076	11745526	12177950	10454748	8990897
1157	E-3,4,5'-Trihydroxy-3'-glucosy lstilbene	$C_9H_{11}NO_3$	[M-H]-	Others	58794	52617	212418	185945	9
1158	chrysotoxine	$C_{11}H_{14}N_2O_3$	$[M+H]^+$	Others	751806	1147913	1656644	806878	1102876
1159	5-Hydroxy-3,4'-dimethoxybibe nzyl*	$C_9H_{11}NO_3$	[M-H]-	Others	1458740	2980638	2020879	1854880	1177600
1160	3,3'-Dihydroxy-5-methoxybibe nzyl	$C_{11}H_{14}N_2O_3$	[M-H]-	Others	960790	1591173	1738628	1934631	1801314
1161	Gigantol(3,3'-Dihydroxy-5,4'-d imethoxybibenzyl)*	$C_9H_{11}NO_3$	$[M+H]^+$	Others	6614748	12135452	11067425	9423297	8630916
1162	Dendrocandin D; 3,4,4'-Trihydroxy-5-methoxy-a lpha-ethoxybibenzyl*	$C_{11}H_{14}N_2O_3$	$[M+H]^+$	Others	4153726	8192507	8468913	5690998	7027227
1163	4',5-Dihydroxy-3,3'-dimethoxy bibenzyl*	$C_9H_{11}NO_3$	$[M+H]^+$	Others	6388583	12104259	11370165	10551937	8496571
1164	Dendrocandin F	$C_{11}H_{14}N_2O_3$	[M-H]-	Others	120884	247999	98331	78651	67117
1165	3,3',5-Trihydroxybibenzyl*	$C_9H_{11}NO_3$	[M-H]-	Others	1827409	3169107	2428711	2160796	2036607
1166	3'-Hydroxy-3,4,5-trimethoxybi benzyl	$C_{11}H_{14}N_2O_3$	$[M+H]^+$	Others	3585200	5055529	4265462	3013810	3363921
1167	4-hydroxy-3,3',5-trimethoxylbi benzyl	$C_9H_{11}NO_3$	$[M+H]^+$	Others	1533736	2036788	1793832	1265942	1309168
1168	3,4,4'-Trihydroxy-5-methoxybi benzyl	$C_{11}H_{14}N_2O_3$	[M-H]-	Others	4337219	9000787	9361111	7051415	7345211
1169	Erianin; 3'-Hydroxy-3,4,4',5-tetrametho xybibenzyl	$C_9H_{11}NO_3$	$[M+H]^+$	Others	88063	158617	155873	96216	110603

1170	Resveratrol	$C_{11}H_{14}N_2O_3$	[M-H]-	Others	186242	345813	610193	448979	729318
1171	4'-Hydroxy-3,5-dimethoxybibenzyl*	$C_9H_{11}NO_3$	[M-H]-	Others	1652476	3369712	2271516	2022693	1399212
1172	Dendrocandin B	$C_{11}H_{14}N_2O_3$	[M-H]-	Others	2043276	3872869	2504617	2216735	2214110
1173	Dendrocandin E; 3,3',4,4'-Tetrahydroxy-5-methoxybibenzyl	$C_9H_{11}NO_3$	[M-H]-	Others	294538	166745	408618	178538	362675
1174	Dihydroresveratrol*	$C_{11}H_{14}N_2O_3$	[M-H]-	Others	2077249	3682332	2798389	2372121	2313591
1175	3,4'-Dihydroxy-5-methoxystilbene	$C_9H_{11}NO_3$	[M-H]-	Others	1041691	1347787	1184034	1234321	589794
1176	Nobilin A	$C_{11}H_{14}N_2O_3$	[M-H]-	Others	3143853	7175878	6011652	5034472	2874871
1177	Dihydropinosylvin	$C_9H_{11}NO_3$	[M-H]-	Others	123493	270248	280065	208593	268395
1178	Dendrocandin G	$C_{11}H_{14}N_2O_3$	[M-H]-	Others	117062	249133	170854	138764	71621
1179	4'-Methoxyresveratrol	$C_9H_{11}NO_3$	[M-H]-	Others	1109966	1349241	1228832	1209003	612916
1180	Trigonopol B	$C_{11}H_{14}N_2O_3$	[M-H]-	Others	689521	1124526	952776	886526	866821
1181	Pterostilbene	$C_9H_{11}NO_3$	[M-H]-	Others	16071	20257	16165	13830	9667
1182	Dendrocandin V	$C_{11}H_{14}N_2O_3$	[M-H]-	Others	45029	123989	99859	61620	90920
